# Isolated α-turn and incipient γ-helix†
†Electronic supplementary information (ESI) available: General information, experimental procedures, compounds characterization, crystallographic data, ^1^H, ^13^C and 2D NMR spectra, FT-IR absorption spectra. CCDC 1906513, 1906514, 1906515, 1906516 and 1906517. For ESI and crystallographic data in CIF or other electronic format see DOI: 10.1039/c9sc01683j


**DOI:** 10.1039/c9sc01683j

**Published:** 2019-06-10

**Authors:** Fatemeh M. Mir, Marco Crisma, Claudio Toniolo, William D. Lubell

**Affiliations:** a Département de Chimie , Université de Montréal , C. P. 6128, Succursale Centre-Ville , Montréal , Québec , Canada H3C 3J7 . Email: william.lubell@umontreal.ca; b Department of Chemistry , University of Padova and Institute of Biomolecular Chemistry , Padova Unit , CNR , 35131 Padova , Italy

## Abstract

The unique abilities of homo-oligo-adamantyl peptides to adopt α- and γ-turn conformations are demonstrated by X-ray diffraction, and NMR and FT-IR absorption spectroscopies. Assembled by an Ugi multiple component reaction strategy, *N*^α^-formyl-adamantyl tripeptide iso-propyl and *tert*-butyl amides are respectively found to adopt an isolated α-turn and an incipient γ-helix conformation by X-ray diffraction crystallography. The shortest example of a single α-turn with ideal geometry is observed in the crystalline state. In solution both peptides predominantly assume γ-helical structures.

## Abstract 2

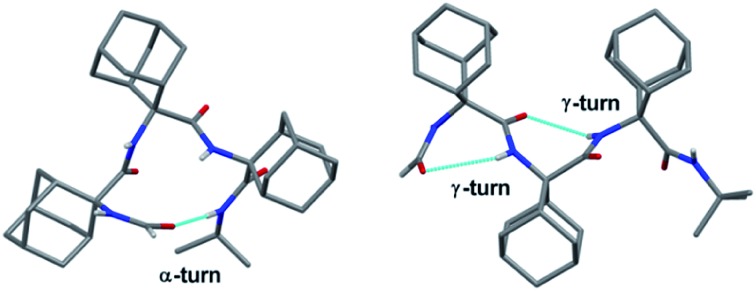
The unique abilities of homo-oligo-adamantyl peptides to adopt α- and γ-turn conformations are demonstrated by X-ray diffraction, and NMR and FT-IR absorption spectroscopies.

## Introduction

Helices constitute the most abundant peptide and protein secondary structures in nature and play vital roles in protein–protein recognition.^1^ Composed of repeating turn motifs, helices are stabilized to a large extent by backbone intramolecular C=O···H–N hydrogen bonds and minimization of side-chain steric interactions.^2^ The most common natural helical structure is the α-helix,2*a* which features 13-membered intramolecular hydrogen bonds between the C=O and NH groups, respectively, of residues *i* and *i* + 4 (α-turn^3^ or C_13_). On the other hand, a single α-turn in short linear peptides (≤5 amino acid residues), unambiguously authenticated by X-ray diffraction analysis, is rare,^4^ due likely to the entropic penalty of forming such an intramolecular hydrogen bond. Conversely, single 7-membered intramolecularly hydrogen-bonded (*i* + 2 → *i* or C_7_) γ-turn structures5*a* have been identified in a few natural and model peptides, but the fully developed γ-helix, featuring repeating γ-turns, has yet to be observed.5*b*

C^α,α^-Disubstituted glycines are known to be able to constrain peptides to adopt distinct secondary structures.^6^,^7^ The backbone *φ* and *ψ* torsion angles adopted by these residues are typically contingent on the substituent size and nature. Specifically, C^α,α^-dimethylglycine (α-aminoisobutyric acid, Aib) and C^α^-methylated analogs of proteinogenic amino acids favor folded backbone conformations in the 3_10_-/α-helical region (*φ* = ±60 ± 20°, *ψ* = ±30 ± 20°),^6^,^8^,^9^ which are similarly adopted by peptides composed of 1-aminocycloalkane-1-carboxylic acid residues with four or more carbon atoms in the ring moiety.6b,^7^,^9^ Homo-peptides from the aforementioned residues almost invariably give rise to 3_10_-helices,6*b* and only exceptionally to α-helices.^10^ These conclusions have been corroborated by a crystal-state investigation on a peptide featuring a single guest Gly residue placed in the middle of a host (Aib)_16_ homo-oligomer.^11^ On the other hand, a few highly sterically hindered residues, including 2-aminoadamantane-2-carboxylic acid (Adm) have been shown by X-ray diffraction analyses on model peptides to exhibit propensity for γ-turn conformations.5b,^12^,^13^ Finally, residues with two ethyl, *n*-propyl, phenyl or benzyl substituents adopt predominantly fully-extended (C_5_) conformations (*φ* ≅ 180°, *ψ* ≅ 180°).6b,^7^,^14^

Although homo-oligomers of Aib have been commonly prepared and studied,^6^,^8^ few examples of long oligopeptides composed of multiple residues with larger side chains have been reported due to the challenge of coupling sterically bulky α-amino acids, particularly at their α-amino functionality.^15^ In this connection, it is worth pointing out that the sterically hindered Adm residue has been shown to react with protein amino acids (Gly, Leu, Phe) in acceptable yields (45–75%) by classical C-activating methods [*i.e*., *N*^α^-unprotected *N*-carboxyanhydride, *N*^α^-acetyl/trifluoroacetyl 5(4*H*)oxazolones and EDC (*N*-ethyl-*N*′-3-(dimethylaminopropyl)carbodiimide)/HOAt (7-aza-1-hydroxy-1,2,3-benzotriazole)] to provide simple dipeptides with *N*-terminal Adm residues.^12^,^16^ The synthesis of dipeptides with C-terminal Adm residues is however more challenging and has been achieved (65–90% yield) using reactive acyl chloride derivatives, combined usually with an azide (–N_3_) precursor of the α-amino group,^12^ according to the procedure of Meldal,^17^ but coupling yields drop precipitously (<10%) upon homo-peptide assembly to the tripeptide level, preventing further main-chain elongation.^12^

Among the potentially useful, alternative methods for synthesizing C^α,α^-disubstituted glycines with very bulky side chains, the Ugi reactions of adamantan-2-one has previously delivered -Adm-Gly- dipeptides, albeit in modest yields.18*a* Furthermore, HCO-Aib-Aib-OMe [OMe, (methoxy)] has been assembled in 57% yield by the reaction of methyl 2-isocyano-2-methylpropanoate, acetone and ammonium formate.18*b* To the best of our knowledge, the construction of longer main chains of severely sterically bulky amino acids by the Ugi reaction has yet to be reported. Exploring the Ugi conditions, we present here access to the HCO-(Adm)_3_-NHR tripeptides **1** and **2** (R = *i*Pr and *t*Bu, respectively). A concomitant combination of X-ray diffraction crystallography and spectroscopic methods has demonstrated that the Adm residue can favor the elusive single 13-membered intramolecularly hydrogen-bonded α-turn, as well as the almost completely developed γ-helix conformations.

## Results and discussion

### Synthesis

**Scheme 1:**
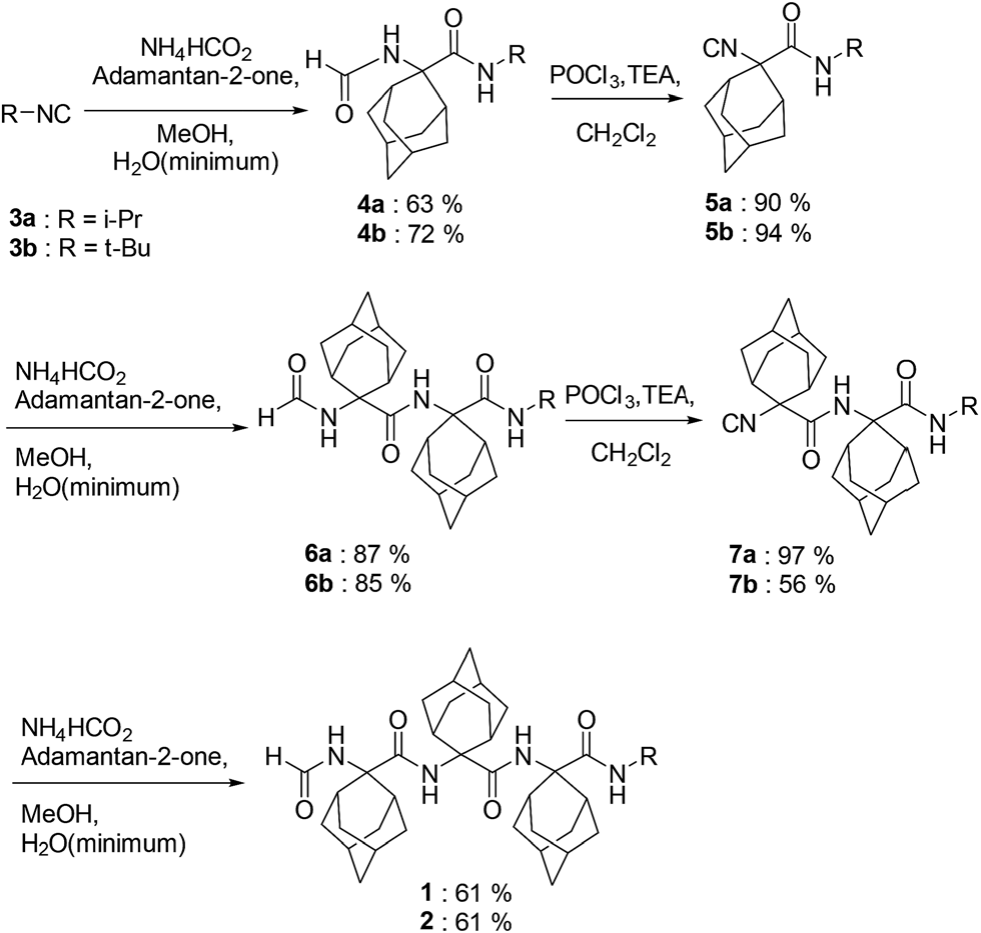
Syntheses of HCO-(Adm)_3_-NHR peptides **1** and **2**.

The synthesis of *N*^α^-formyl-Adm homo-tripeptides **1** and **2** began respectively by reacting adamantan-2-one with iso-propyl and *tert*-butyl isocyanides **3** in methanol (MeOH) with ammonium formate dissolved in the minimum amount of water (Ugi conditions, Scheme 1). After heating at 70 °C for 20 hours, evaporation of the volatiles and chromatography on silica gel afforded the formamides **4**. Isocyanides **5** were prepared on treatment of **4** with POCl_3_ and triethylamine in dichloromethane (DCM) between –5 to –10 °C for 1–2 hours. After aqueous workup and column chromatography, isocyanides **5** were isolated as solids in ≥90% yields. Exposure of isocyanides **5** to similar Ugi conditions as those described above gave homo-dipeptides **6** as solids in ≥85% yields. Subsequently, formamide to isocyanide conversion went smoothly using the POCl_3_ conditions to afford isocyanides **7**. Finally, the desired homo-tripeptides **1** and **2** were synthesized using the Ugi approach. However, the reaction was relatively sluggish, and required heating for 2 days in the presence of excess ammonium formate to provide solid products after chromatography in about 60% yields.

### Conformational analysis of tripeptides 1 and 2

#### X-ray diffraction analysis

**Fig. 1:**
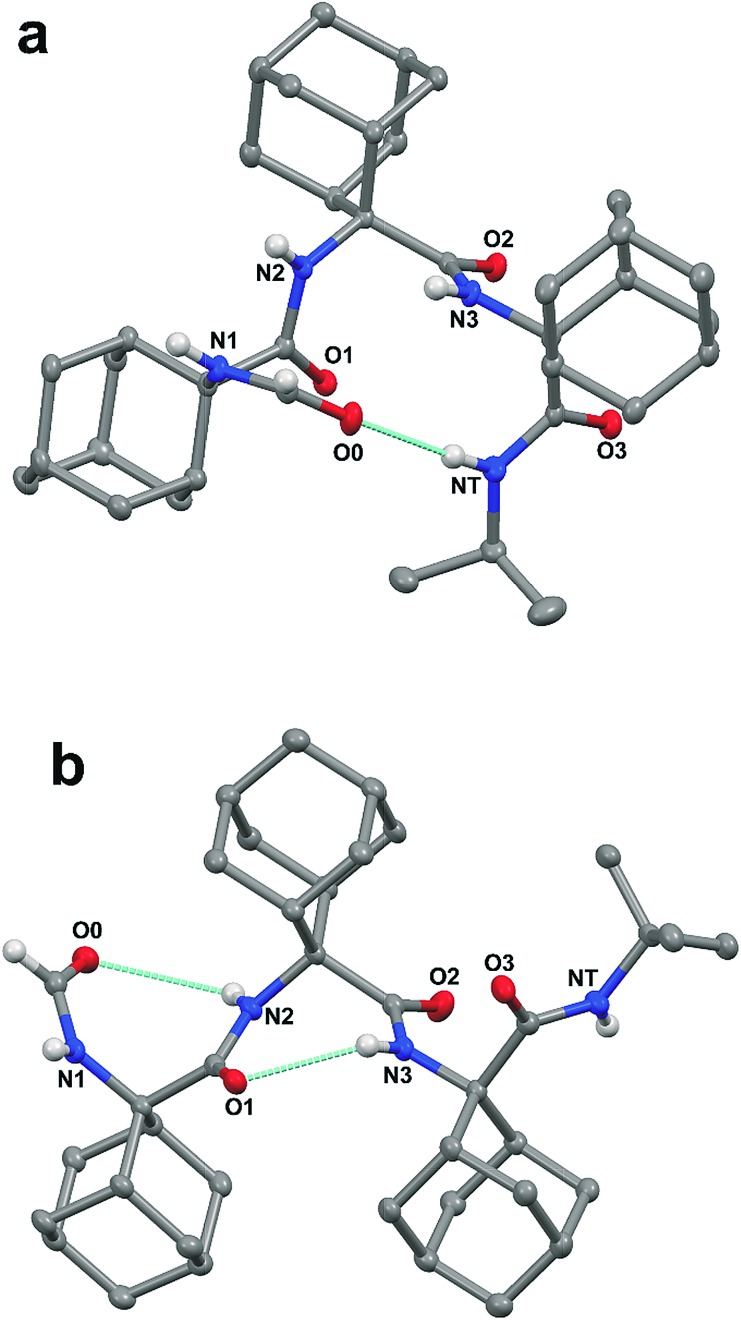
X-ray diffraction crystal structures of the HCO-(Adm)_3_-NHR peptides **1** (a), crystallized from acetone, and **2** (b), crystallized from acetone/EtOAc (ethyl acetate). Most of the H-atoms are omitted for clarity. Intramolecular hydrogen bonds are indicated by dashed lines.

The three-dimensional structures of the Adm homo-tripeptides **1** and **2** were determined by single crystal X-ray diffraction (Fig. 1) and their backbone torsion angles (Table 1) were compared with those of ideal secondary structures. A single 13-membered intramolecularly hydrogen-bonded α-turn is observed in the asymmetric unit of **1** (R = *i*Pr) (Fig. 1a and ESI†). This intramolecular hydrogen bond takes place between the formamide carbonyl oxygen and the C-terminal amide NH group [N···O and H···O separations 3.095(3) Å and 2.22 Å, respectively; N–H···O angle 177.6°].

**Table 1:** Backbone torsion angles (°) of the HCO-(Adm)_3_-NHR peptides **1** (R = *i*Pr) and **2** (R = *t*Bu) from X-ray diffraction analyses

Entry	*φ*_1_	*ψ*_1_	*φ*_2_	*ψ*_2_	*φ*_3_	*ψ*_3_
**1** From acetone	–52.4	–49.0	–59.3	–46.5	–59.7	–59.6
**1** From acetone/CHCl_3_	–52.8	–48.6	–59.6	–46.0	–59.5	–60.3
**1** From acetone/EtOAc/CHCl_3_	–52.8	–48.8	–59.5	–46.0	–59.6	–60.2
**2** From acetone/EtOAc	82.1	–84.1	74.3	–82.3	80.3	–113.2

In addition, the formamide carbonyl oxygen is at a distance of 3.117(3) Å from the nitrogen atom of Adm(3), suitable for forming a 10-membered intramolecular hydrogen bond (β-turn),^19^ but the H···O separation (2.61 Å) and N–H···O angle (117.4°) would be slightly outside the commonly accepted limits (≤2.50 Å and ≥ 120°) for the occurrence of a hydrogen bond.^20^ In the quest for possible polymorphs, peptide **1** was also crystallized from solvents of varying polarity (EtOAc, acetone, CHCl_3_) within the limits imposed by solubility. Crystals suitable for X-ray diffraction analysis were obtained from three different systems; however, all gave the identical α-turn structure, within experimental error (Table 1).

As stated above, crystallography of single α-turns is very unusual.^4^ In most of the examples, the backbone torsion angles are significantly distorted in comparison with those observed in regular α-helices. Conversely, in the crystal structure of **1**, the average *φ* and *ψ* torsion angles over the three Adm residues (–57°, –52°) differ only by 6° and 10°, respectively, from the canonical values (–63°, –42°) based on statistical analysis of α-helices in crystalline peptides.2*c* To the best of our knowledge, the structure of **1** represents the shortest peptide sequence giving rise to an isolated α-turn of regular geometry.

In the X-ray diffraction structure of tripeptide **2**, the backbone torsion angles (Table 1) and distances between hydrogen-bond donor and acceptor atoms demonstrate the presence of two consecutive γ-turns5*b* at the level of the Adm(1) and Adm(2) residues (Fig. 1b and ESI†). Two intramolecular hydrogen bonds are observed, namely between the formamide carbonyl oxygen and the NH group of Adm(2), and between the carbonyl oxygen of Adm(1) and the NH group of Adm(3). The former hydrogen bond is significantly elongated [N···O 3.1665(15) Å, H···O 2.572(18) Å, N–H···O angle 129.4(15)°] in comparison to the latter [N···O 2.8804(14) Å, H···O 2.31(2) Å, N–H···O angle 124.5(16)°]. Indeed, the backbone torsion angles of Adm(1) deviate more markedly than those of Adm(2) from the ideal values of a γ-turn structure (*φ*, *ψ* = 75°,–75°).^5^ The shorter intramolecular hydrogen bond may be due in part to London dispersion between the adamantane moieties of Adm residues 1 and 3;^21^ three H···H contacts (the shortest of which separated by 2.34 Å) are observed. The longer intramolecular hydrogen bond is associated with a 7.8° larger *φ*_1_ value, compared to that of *φ*_2_, in contrast to the less than 2° difference in the values of *ψ*_1_ and *ψ*_2_. The distortion of *φ*_1_ weakens the N2–H···O0 intramolecular hydrogen bond, but enables the N1–H group to participate in an intermolecular hydrogen bond with the O2 atom of a symmetry related molecule (ESI†). The torsion angles of Adm(3) fall also in the lower-right quadrant of the Ramachandran map as those of the two preceding residues, but formation of a third γ-turn is not observed. Conversely, the carbonyl oxygen of Adm(2), which could be the potential acceptor of the γ-turn hydrogen bond, is involved in an intermolecular hydrogen bond with the N1–H group of a symmetry-related molecule (ESI†), and the C-terminal amide NH group is not engaged in any hydrogen bond. To avoid intermolecular steric clashes in the crystal, the *ψ* torsion angle of Adm(3) is forced to adopt a value (–113.2°) which prevents the C-terminal amide NH group from approaching the carbonyl oxygen of Adm(2) at a suitable hydrogen-bond distance.

The calculated density for the crystals of **1** (1.254 g cm^–3^) was less than that of **2** (1.302 g cm^–3^). The packing mode of tripeptide **1** features one N–H···O=C hydrogen bond, three C–H···O contacts within the distance of 2.66 Å,^22^ and four H···H contacts within van der Waals distance. In the packing mode of tripeptide **2**, intermolecular interactions include one N–H···O=C hydrogen bond, as well as two C–H···O contacts and nine aliphatic H···H contacts in the range 2.10–2.39 Å. Within the sum of the van der Waals radii, the latter may stabilize the packing and account for the higher density of crystals of **2** through London dispersion.^21^

#### NMR and FT-IR absorption spectroscopy

To study the conformation of peptides **1** and **2** in solution, NMR and FT-IR absorption spectroscopic methods were employed.

Sequence assignments of the NH proton resonances in the NMR spectra of peptides **1** and **2** in CDCl_3_ were achieved by a combination of COSY and HMBC experiments, the latter optimized to identify long-range, through-bond *J* couplings (ESI†).

**Fig. 2:**
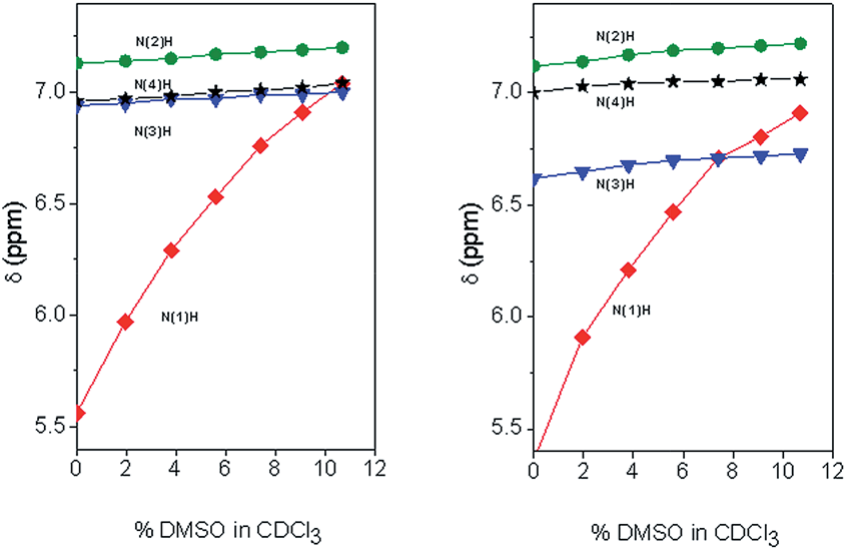
Plot of the chemical shifts of the NH signals in the NMR spectra of HCO-(Adm)_3_-NH*i*Pr **1** (left) and HCO-(Adm)_3_-NH*t*Bu **2** (right) as a function of the addition of increasing percentages (v/v) of DMSO to the CDCl_3_ solution. Peptide concentration: 1.0 mM.

Solvent shielded and exposed NH protons were examined by studying influences of changes in environment on the chemical shifts of their signals (Table 2). Amide protons involved in hydrogen bonds display typically little variation in their chemical shifts upon changes in solvent composition and temperature.^23^ As for peptides **1** and **2**, switching NMR solvent from CDCl_3_ to dimethylsulfoxide (DMSO, *d*_6_) caused significant (2.37 and 2.58 ppm, respectively) downfield shifts for only the formamide NH proton signal, suggesting that this group is exposed to solvent in contrast to the other NH proton signals which exhibited limited variations in chemical shifts (0.02 to 0.22 ppm). This different behavior of the formamide NH proton is already evident at the very beginning (2% DMSO in CDCl_3_) of the solvent titration curves (Fig. 2). Similarly, for both peptides **1** and **2**, changes in temperature in the two solvents influenced significantly (Δ*δ*/Δ*K* = –3.8 to –5.2 ppb K^–1^) the formamide NH signal relative to those of the other amide protons. The NH chemical shifts of the latter protons varied less with temperature in the solvent of lower polarity CDCl_3_ (Δ*δ*/Δ*K* = –0.3 to –1.5 ppb) than in DMSO (Δ*δ*/Δ*K* = –1.0 to –3.3 ppb K^–1^).

**Table 2:** Influence of solvent and temperature on the chemical shifts of the NH signals of HCO-(Adm)_3_NHR peptides **1** and **2** in CDCl_3_ and DMSO

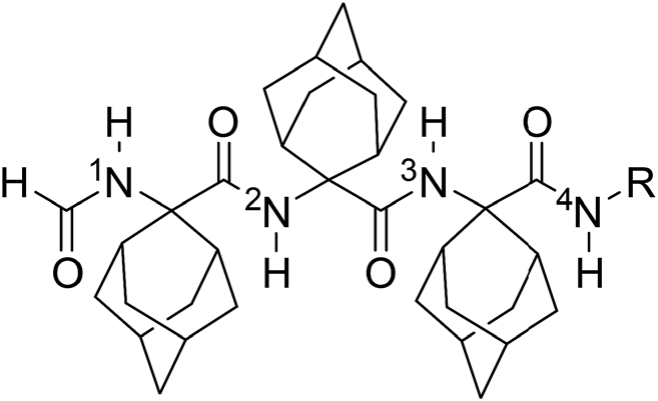					
entry	*δ* CDCl_3_	*δ* DMSO	Δ*δ*/Δsol	Δ*δ*/Δ*K*	Δ*δ*/Δ*K*
				CDCl_3_	DMSO

**1: R = *i*Pr**					
^1^NH	5.67	8.04	2.37	–4.3	–4.2
^2^NH	7.17	7.37	0.20	–0.3	–1.2
^3^NH	7.00	7.02	0.02	–1.4	–1.5
^4^NH	7.02	7.18	0.16	–1.3	–3.3

**2: R = *t*Bu**					
^1^NH	5.46	8.04	2.58	–5.2	–3.8
^2^NH	7.16	7.38	0.22	–0.3	–1.1
^3^NH	6.69	6.91	0.22	–0.45	–1.0
^4^NH	7.07	7.05	0.02	–1.5	–2.5

**Fig. 3:**
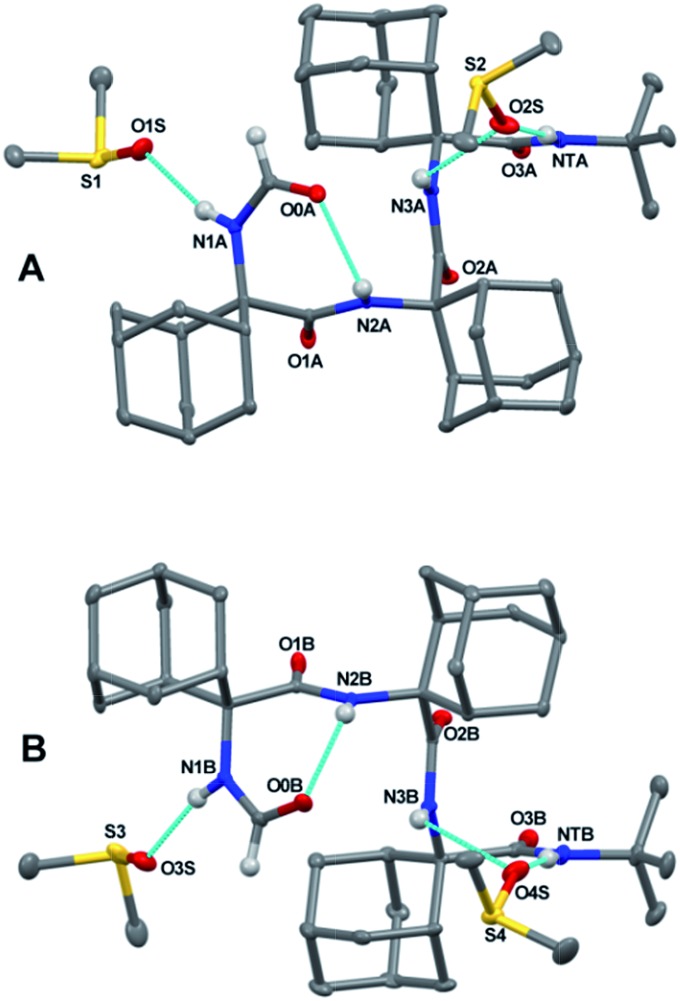
The two independent molecules (**A** and **B**) in the X-ray diffraction crystal structure of peptide **2** bis-DMSO solvate. Most of the H-atoms are omitted for clarity. Intramolecular and peptide-solvent hydrogen bonds are indicated by dashed lines.

Notably, an X-ray diffraction analysis of crystals of peptide **2** grown from DMSO (bis-DMSO solvate) highlighted the presence of only one γ-turn in each of the two independent but similar peptide conformers **A** and **B** in the crystal matrix (Fig. 3 and ESI†). The surviving intramolecular hydrogen bond occurred between the formamide carbonyl oxygen and the NH group of Adm(2). The sets of *φ* and *ψ* torsion angles of Adm(1) in the two peptide conformers were –75.4°, 74.8°, and 76.6°, –69.9°, respectively. Three intermolecular hydrogen bonds were observed between each peptide and two co-crystallized DMSO solvent molecules: one between the Adm(1) NH group and the oxygen atom of one molecule of DMSO, and the other two between the Adm(3) and *tert*-butyl amide NH groups together with the oxygen atom of a second DMSO molecule. In each peptide conformer, Adm(2) and Adm(3) were helical but of opposite screw sense: *φ*_2_, *ψ*_2_, *φ*_3_, *ψ*_3_ = 59.8°. 64.1°, –59.0°, –55.5° (in **A**); –60.0°, –66.9°, 61.1°, 58.5° (in **B**).

**Fig. 4:**
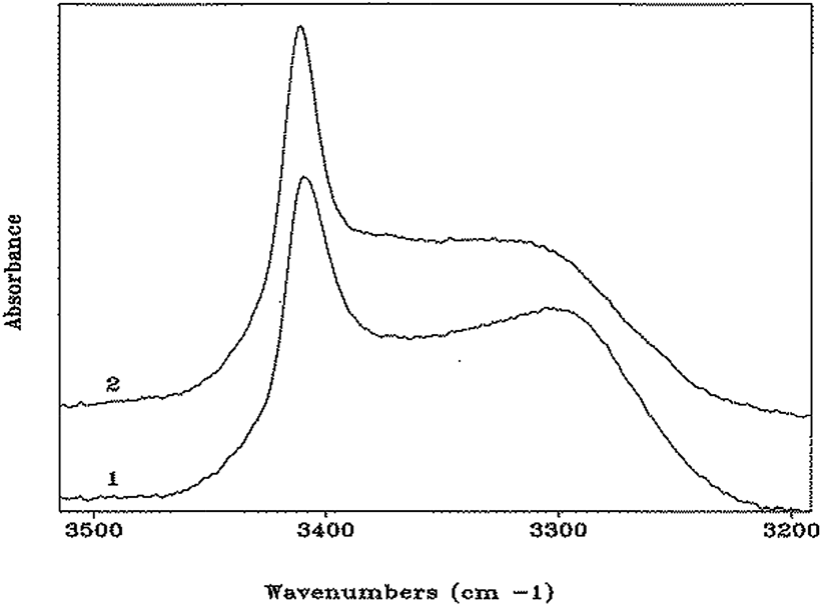
FT-IR absorption spectra (N–H stretching region) of HCO-(Adm)_3_-NH*i*Pr (**1**) and HCO-(Adm)_3_-NH*t*Bu (**2**) in CDCl_3_ solution. Peptide concentration: 1.0 mM.

In the N–H stretching region (3500–3200 cm^–1^), the FT-IR absorption spectra of peptides **1** and **2** in CDCl_3_ solution (Fig. 4) are very similar, and characterized by a band at 3412–3414 cm^–1^ (free, solvated NH groups) accompanied by a much broader band with maximum near 3300 cm^–1^ (hydrogen-bonded NH groups).^24^ The latter band is significantly skewed to higher wavenumbers. For both compounds, no significant differences in spectral shape and in the relative intensity of the hydrogen-bonded *versus* free N–H stretching bands were found over the concentration range 10.0 mM–0.1 mM (ESI, Fig. S3 and S4†), suggesting strongly that the observed hydrogen bonding is essentially intramolecular. In addition, these curves do not provide any evidence for concentration-dependent conformational transitions. The solid-state FT-IR spectra of the crystals of peptides **1** and **2** have also been recorded (ESI, Fig. S5 and S6†). They are clearly different, particularly in the N–H stretching region. However, unambiguous dissection of contributions from inter- and intramolecular hydrogen bonds to the observed bands is problematic (in spite knowledge of hydrogen-bonding and related geometrical parameters from the X-ray crystal structures), thus hampering a meaningful correlation with the spectra in solution. Overall, the FT-IR spectra of both peptides in solution are compatible with the occurrence of multiple, more-or-less strongly hydrogen-bonded, γ-turns. Conversely, a prevailing population of a single α-turn or combinations of two β-turns or a β-turn encompassed within an α-turn, all are unlikely. These conformers, in which the ratio of free to hydrogen bonded NH groups would be respectively 3/1 and 2/2, would exhibit spectral shapes different from those reported in Fig. 4. Moreover, the maximum of the band associated with hydrogen-bonded N–H groups in α- or β-turns would be located at wavenumbers significantly higher than 3300 cm^–1^.^24^ Taken together, the observed NMR and FT-IR absorption data indicate that both peptides assume predominantly γ-helical structures in solution. The capabilities of these spectroscopic techniques are however unable to detect or rule out the presence of a concomitant small population of α-turn conformation.

Considering the marginal role of the crystallization solvent properties to significantly bias the conformations of peptides **1** and **2**, the chemical structure of the C-terminal substituent R (*i*Pr or *t*Bu) is likely responsible for the outcome in the crystal states (α-turn or γ-turn). An example has already been reported of two homo-peptides differing only in their C-terminal group (ethyl ester *vs. tert*-butyl ester) giving rise to two different conformations in the crystal state, but adopting apparently the same conformation in solution.14a,b In such cases, self-assembly processes leading to crystal nucleation may favor a minor conformer present in solution depending on the C-terminal group. Subsequently, crystal growth will be governed by the templating effect of the crystal nuclei. A synthetic and conformational study on a large set of appropriately designed model compounds is planned in our laboratories, aimed at further exploring the effect of the C-terminal substituent on the 3D-structure of Adm-based peptides.

## Conclusions

To summarize, a novel method for assembling sterically congested peptides from C^α,α^-disubstituted glycines bearing bulky side chains using the Ugi multiple component reaction has been achieved and applied in the synthesis of *N*^α^-formyl adamantyl tripeptide amides **1** and **2**. Conformational analysis has revealed the potential for the Adm residue to favor the unprecedented single α-turn with ideal *φ* and *ψ* torsion angles and an incipient γ-helical structure. Notably, the overwhelming majority of C^α^-tetrasubstituted α-amino acids prefer 3_10_-helices, which are generated by consecutive type-III (III') β-turns, and subsequently α-helix conformers.2c,6b Conversely, access to the 3_10_-helix appears to be unavailable to the Adm residue. In the *φ* and *ψ* space, the most significant difference between the two conformations is the value of the *ψ* torsion angle. For the Adm residue, typical *ψ* values for right- and left-handed 3_10_-helices (–35° to –25° and 35° to 25°, respectively) have never been observed, likely due to a destabilizing aliphatic C–H···O interaction between the *pro-R* or *pro-S* γ-CH_2_ groups with the carbonyl oxygen atom, which would require an energetically disfavored H···O separation of the order of 2.10 Å.^22^ This sterically unfavorable situation is relieved in part by increasing the *ψ* values to those observed in the α-helical turn of peptide **1** (in the range –46.5° to –59.6°) which resulted in H···O separations between 2.23 and 2.33 Å, and to a larger extent in a γ-turn conformation (peptide **2**), in which the observed H···O separations are >2.40 Å.

Considering the power of the Ugi multiple component method reported in this work for synthesizing sterically hindered peptides, potential now exists to harness bulky residues for various studies in peptide science. The unique γ-helical folding pattern which was observed both in solution and in the crystal state, as well as the fascinating diamondoid hydrocarbon side chains may similarly lend themselves for novel applications. In this connection, adamantane-functionalized compounds [particularly its –NH- and –C(=O)– derivatives] have already found important applications in medicinal chemistry as drugs to treat parkinsonism and related syndromes, and as antiviral agents to combat type-A influenza virus in humans.^25^ Moreover, growing interest for adamantane and related structures is currently being observed in other areas, including nanotechnology (as organic microelectronic components and coating agents), polymers, molecular recognition, and supramolecular chemistry.^26^

## Conflicts of interest

There are no conflicts to declare.

## Supplementary Material

Supplementary information

Crystal structure data
