# Supplementary material for: Isolated α-turn and incipient γ-helix
Source: Chem Sci. 2019 Jun 10;10(28):6908–14. doi: 10.1039/c9sc01683j (PMC6640192; doi:10.1039/c9sc01683j)
Supplement: Supplementary file 1 [file SC-010-C9SC01683J-s001.pdf]

Electronic Supplementary Material (ESI) for Chemical Science.
This journal is © The Royal Society of Chemistry 2019

Supporting Information

Isolated α-Turn and Incipient γ-Helix

Fatemeh M. Mir,[a] Marco Crisma,[b] Claudio Toniolo,[b] William D. Lubell[a]*

[a] Département de Chimie, Université de Montréal, C. P. 6128, Succursale Centre-Ville, Montréal, Québec, Canada H3C 3J7

[b] Department of Chemistry, University of Padova and Institute of Biomolecular Chemistry, Padova Unit, CNR, 35131 Padova, Italy

E-mail : william.lubell@umontreal.ca

Table of Contents

Experimental section……..……………………………………..…………………………..……..S4

General Methods …..………………………………………………………………………………..S4

Reagents ………………………………………………………………………………………………S5

Synthesis of:

2-*N*-(Formyl)aminoadamantane-2-*N*'-(*iso*-propyl)carboxamide (**4a**)……………………….……..S5

2-*N*-(Formyl)aminoadamantane-2-*N*'-(*tert*-butyl)carboxamide (**4b**) ……………..……….........S5-S6

2-Isocyanoadamantane-2-*N*'-(*iso*-propyl)carboxamide (**5a**) …………………………….…...........S6

2-Isocyanoadamantane-2-*N*'-(*tert*-butyl)carboxamide (**5b**)…………...S6

Formyl-Adm-Adm-NH-*i*-Pr (**6a**) ………………………………………………………………….……..S7

Formyl-Adm-Adm-NH-*t*-Bu (**6b**) S7

'Isocyano'-Adm-Adm-NH-*i*-Pr (**7a**) …………...………………………...…………..……S7-S8

'Isocyano'-Adm-Adm-NH-*t*-Bu (**7b**) ……………………………………….…....................S8

Formyl-Adm-Adm-Adm-NH-*i*-Pr (**1**) ………….………………………………...…………S8-S9

Formyl-Adm-Adm-Adm-NH-*t*-Bu (**2**) ………………………………………………...……..S9

X-Ray diffraction..S9-S10

Crystallographic data for peptide **1** from acetone...S11-S13

Crystallographic data for peptide **1** from acetone / $CHCl_3$ ……………………………….……S14-S16

Crystallographic data for peptide **1** from acetone / EtOAc / $CHCl_3$ ………………………… .S17-S19

Crystallographic data for peptide **2**..S20-S22

Crystallographic data for peptide **2** *bis*- DMSO solvate..S23-S28

NMR Spectra...S29-S43

^{1}H, ^{13}C NMR spectra for amide **4a**...S29

^{1}H, ^{13}C NMR spectra for amide **4b**...S30

^{1}H, ^{13}C, HSQC NMR spectra for isonitrile **5a**...S31-S32

^{1}H, ^{13}C NMR spectra for isonitrile **5b**..S33

^{1}H, ^{13}C, COSY, HSQC NMR spectra for peptide **6a**..S34-S35

^{1}H, ^{13}C NMR spectra for peptide **6b**...S36

^{1}H, ^{13}C NMR spectra for peptide **7a**...S37

^{1}H, ^{13}C NMR spectra for peptide **7b**...S38

^{1}H, ^{13}C, COSY, HMBC NMR spectra for peptide **1**..S39-S40

^{1}H, ^{13}C, COSY, HMBC NMR spectra for peptide **2**..S41-S43

Effect of temperature changes on N-H signal chemical shifts (400 MHz) of **1** in $CDCl_3$..............S44

Effect of temperature changes on N-H signal chemical shifts (400 MHz) of **2** in $CDCl_3$..............S44

Effect of temperature changes on N-H signal chemical shifts (400 MHz) of **1** in DMSO-*d6*….....S45

Effect of temperature changes on N-H signal chemical shifts (400 MHz) of **2** in DMSO-*d6*……S45

Figure S1. Plot of changes of NH signal chemical shifts in the NMR spectra of peptides **1** and **2** as a function of temperature in $CDCl_3$………………………………………………...…S46

Figure S2. Plot of changes of NH signal chemical shifts in the NMR spectra of peptides **1** and **2** as a function of temperature in DMSO-*d6*……………………………….......……S46

Figure S3. Overlay of the FT-IR absorption spectra (N-H stretching region) of peptide **1** in $CDCl_3$ solution at the concentrations 10.0 mM, 1.0 mM, and 0.1 mM……..………………..…...S47

Figure S4. Overlay of the FT-IR absorption spectra (N-H stretching region) of peptide **2** in $CDCl_3$ solution at the concentrations 10.0 mM, 1.0 mM, and 0.1 mM……..……………….....S47

Figure S5. Solid-state FT-IR absorption spectrum of crystals of peptide **1**……………………...S48

Figure S6. Solid-state FT-IR absorption spectrum of crystals of peptide **2**…………………..….S49

Supporting References…………………………………………………………...........…….S49

EXPERIMENTAL SECTION

General Methods: Unless otherwise specified, all non-aqueous reactions were performed under an inert argon atmosphere. Anhydrous DCM was obtained by passage through a solvent filtration system (Glass Contour, Irvine, CA) and transferred by syringe. Reaction mixture solutions (after aqueous workup) were dried over anhydrous Na_2SO_4, filtered, and rotary-evaporated under reduced pressure. Column chromatography was performed on 230-400 mesh silica gel, and thin-layer chromatography was performed on alumina plates coated with silica gel (Merck 60 F_{254} plates). Visualization of the developed chromatogram was performed by UV absorbance or staining with iodine. Melting points were obtained on a Buchi melting point B-540 apparatus and are uncorrected. Accurate mass measurements were performed on an LC-MSD instrument in electrospray ionization (ESI-TOF) mode at the Université de Montréal Mass Spectrometry facility, and are listed as empirical formula confirmations $[M + H]^+$. Nuclear magnetic resonance (NMR ^1H, ^{13}C, COSY, HMBC) spectra were recorded on Bruker 300, 400, 500 and 700 MHz spectrometers. ^1H and ^{13}C NMR spectra were respectively referenced to $CDCl_3$ (7.26 ppm and 77.16 ppm) or DMSO-d_6 (2.50 ppm, and 39.52 ppm). Coupling constant *J* values were measured in Hertz (Hz) and chemical shift values in parts per million (ppm). Infrared absorption spectra for characterization of compounds were recorded on a Bruker Alpha P FT-IR spectrometer equipped with a single reflection ATR sampling module which allows spectral acquisition from neat solid and liquid samples. Band positions are reported in reciprocal centimeters (cm^{-1}). The FT-IR absorption spectra in $CDCl_3$ (99.8% *d*; Merck) solution were recorded at 293 K using a Perkin-Elmer model 1720X FT-IR spectrophotometer, nitrogen flushed, equipped with a sample-shuttle device, at 2 cm^{-1} nominal resolution, averaging 100 scans. Solvent (baseline) spectra were obtained under the same conditions. For spectral elaboration, the software SpectraCalc (Galactic) was employed. Cells were used with CaF_2 windows and path lengths of 0.1 mm, 1.0 mm, and 10.0 mm.

Reagents: Unless specified otherwise, commercially available reagents were purchased from Aldrich, A & C American Chemicals Ltd., Fluka and Advanced Chemtech™ and used without further purification, including adamantan-2-one, ammonium formate, formic acid, $POCl_3$, and Et_3N.

SYNTHESIS

2-*N*-(Formyl)aminoadamantane-2-*N*'-(*iso*-propyl)carboxamide (**4a**)

A solution of *iso*-propyl isocyanide (**3a**, 1 g, 14.5 mmol, 1eq) in MeOH (2M) was treated with adamantan-2-one (2.2 g, 14.5 mmol, 1eq) and ammonium formate (1.1 g, 17.4 mmol, 1.2eq, dissolved in the minimum amount of H_2O). The mixture was stirred for 16h. The volatiles were evaporated. The residue was dissolved in $CHCl_3$, washed with water and brine, and dried over Na_2SO_4. The volatiles were evaporated and the residue was purified by column chromatography using 40% EtOAc in hexanes as eluent. Evaporation of the collected fractions gave Formyl-Adm-NH-*i*-Pr (**4a**, 2.4 g, 62%) as white powder: R_f 0.33 (3:97 MeOH:CH_2Cl_2); m.p. 181-185 °C; ^1H NMR (500 MHz, $CDCl_3$) δ 1.13 (d, J = 6.5, 6H), 1.65-1.74 (m, 6H), 1.80-1.82 (m, 2H), 1.92-1.96 (m, 4H), 2.64 (br, 2H), 4.01-4.07 (m, 1H), 6.07 (s, 1H), 6.96 (d, J = 7.5, 1H), 8.12 (d, J = 2.5, 1H); ^{13}C NMR (500 MHz, $CDCl_3$): δ 22.6, 26.5, 26.6, 32.1, 32.6, 34.0, 37.4, 41.4, 64.9, 161.7, 171.2; IR (neat) ν = 3320, 3286, 2903, 1652, 1518; HRMS (ESI) *m*/*z* calcd for $C_{15}H_{25}N_2O_2$ 265.1911; found $[M + H]^+$ 265.1916.

2-*N*-(Formyl)aminoadamantane-2-*N*'-(*tert*-butyl)carboxamide (**4b**)

Amide **4b** was synthesized from *tert*-butyl isocyanide (**3b**, 3 g, 41.6 mmol) according to the procedure described for the synthesis of formamide **4a** and purified by column chromatography using 30% EtOAc in hexanes as eluent. Evaporation of the collected fractions gave Formyl-Adm-NH-*t*-Bu (**4b**, 8.3g, 72%) as a white powder: R_f 0.2 (7:3 hexanes:EtOAc); m.p. 197-200 °C; ^1H NMR (500 MHz, $CDCl_3$): δ 1.32 (s, 9H), 1.3-1.72 (m, 6H), 1.79-1.84 (m, 2H), 1.92-2.00 (m, 4H), 2.60 (br, 2H), 6.16 (s, 1H), 6.95 (s, 1H), 8.11 (d, J = 2.0 Hz, 1H); ^{13}C NMR (500 MHz, $CDCl_3$) δ 26.4, 26.6, 28.6, 32.1, 32.6, 34.1, 37.3, 51.0, 65.4, 161.7, 171.0; IR (neat) ν: 3264, 2906, 1674,

1649, 1540, 1222, 741; HRMS (ESI) *m*/*z* calcd for $C_{16}H_{27}N_2O_2$ 279.2067; found $[M + H]^+$ 279.2066.

2-Isocyanoadamantane-2-*N*'-(*iso*-propyl)carboxamide (**5a**)

Formamide **4a** (2 g, 7.6 mmol) in CH_2Cl_2 (8 mL) was treated with Et_3N (6.36 g, 45.6 mmol), cooled to –5 °C, and treated dropwise with $POCl_3$ (1.07 g, 11.4 mmol). After stirring at this temperature for 1-2 h, the reaction mixture was vigorously stirred and treated with a solution of saturated $NaHCO_3$ (8mL). The organic phase was separated, washed with brine, and dried over Na_2SO_4. Evaporation of the volatiles under reduced pressure afforded a yellow powder, which was purified by column chromatography using 30% EtOAc in hexanes as eluent. Evaporation of the collected fractions gave isonitrile **5a** (1.70 g, 90%) as white powder: R_f 0.53 (7:3 hexanes:EtOAc); m.p. 130-133 °C; ^1H NMR (500 MHz, $CHCl_3$) δ 1.18 (d, *J* = 7.0, 6H), 1.72 (m, 2H), 1.76-1.78 (m, 4H), 1.81 (m, 1H), 1.89-1.91 (m, 1H), 1.96-1.99 (m, 2H), 2.23-2.26 (m, 2H), 2.31 (m, 2H), 4.07-4.14 (m, 1H), 5.74 (br, 1H); ^{13}C NMR (500 MHz, $CDCl_3$) δ 22.4, 26.2, 26.4, 33.3, 35.0 (2C), 37.3, 42.1, 68.6, 158.2, 166.7; IR (neat): ν = 3315, 2915, 2122, 1647, 1533, 653; HRMS (ESI) *m*/*z* calcd for $C_{15}H_{23}N_2O$ 247.1805; found $[M + H]^+$ 247.1810.

2-Isocyanoadamantane-2-*N*'-(*tert*-butyl)carboxamide (**5b**)

Isonitrile **5b** was synthesized from formamide **4b** (6.75 g, 24.24 mmol) according to the procedure described for the synthesis of isocyanide **5a** and purified by column chromatography using 10% EtOAc in hexanes as eluent. Evaporation of the collected fractions gave isocyanide **5b** (6 g, 94%) as white powder: R_f 0.36 (9:1 hexanes:EtOAc); m.p. 116-120 °C; ^1H NMR (500 MHz, $CHCl_3$) δ 1.37 (s, 9H), 1.71 (m, 2H), 1.75-1.77 (m, 4H), 1.81 (m, 1H), 1.89 (m, 1H), 1.94-1.97 (m, 2H), 2.22-2.24 (m, 2H), 2.28 (m, 2H), 5.69 (s, 1H); ^{13}C NMR (500 MHz, $CDCl_3$) δ 26.2, 26.3, 28.5, 33.3, 35.1, 37.2, 39.4, 51.8, 68.9, 158.0, 166.6; IR (neat) ν: 3363, 2917, 2120, 1660, 1527, 1452, 1214, 583; HRMS (ESI) *m*/*z* calcd for $C_{16}H_{25}N_2O$ 261.19614; found $[M + H]^+$ 261.19712.

Formyl-Adm-Adm-NH-*i*-Pr (6a)

Dipeptide **6a** was synthesized from isonitrile **5a** (1.6 g, 6.47 mmol) according to the procedure described for the synthesis of formamide **4a** and purified by column chromatography using 40% hexanes in EtOAc as eluent. Evaporation of the collected fractions gave HCO-Adm-Adm-NH-*i*-Pr (**6a**, 2.5 g, 87%) as white powder: R_f 0.25 (4:6 hexanes:EtOAc); m.p. 192-198 °C; ^{1}H NMR (500 MHz, $CHCl_3$) δ 1.10 (d, J = 6.6 Hz, 6H), 1.62 (m, 1H), 1.65 (m, 1H), 1.69-1.70 (m, 9H), 1.76 (m, 1H), 1.79-1.83 (m, 4H), 1.93-1.95 (m, 6H), 2.00 (m, 1H), 2.03 (m, 1H), 2.64-2.66 (m, 4H), 3.99-4.07 (m, 1H), 5.74 (s, 1H), 6.81 (d, J = 7.9, 1H), 6.92 (s, 1H), 8.13 (d, J = 1.9, 1H); ^{13}C NMR (500 MHz, $CDCl_3$) δ 22.8, 26.4, 26.6 (2C), 26.9, 32.2, 32.6, 32.7, 32.8, 34.0, 34.2, 37.3, 37.7, 41.1, 64.7, 65.3, 161.3, 171.2, 172.1; IR (neat) ν: 3360, 2904, 1677, 1511, 751. HRMS (ESI) *m*/*z* calcd for $C_{26}H_{40}N_3O_3$ 442.3060; found $[M + H]^+$ 442.3073.

Formyl-Adm-Adm-NH-*t*-Bu (6b)

Dipeptide **6b** was synthesized from isonitrile **5b** (2 g, 7.65 mmol) according to the procedure described for the synthesis of formamide **4a** and purified by column chromatography using 30% EtOAc in hexanes as eluent. Evaporation of the collected fractions gave HCO-Adm-Adm-NH-*t*-Bu (**6b**, 2.96 g, 85%) as white powder: R_f 0.21 (7:3 hexanes:EtOAc); m.p. 228-230 °C; ^{1}H NMR (500 MHz, $CDCl_3$) δ 1.30 (s, 9H), 1.62 (m, 1H), 1.64 (m, 1H), 1.68-1.72 (m, 8H), 1.75 (m, 1H), 1.78-1.82 (m, 5H), 1.94-1.96 (m, 6H), 2.00 (m, 1H), 2.03 (m, 1H), 2.63 (m, 2H), 2.68 (m, 2H), 5.55 (s, 1H), 6.83 (s, 1H), 6.93 (s, 1H), 8.13 (d, J = 1.9 Hz, 1H); ^{13}C NMR (500 MHz, $CDCl_3$): δ 26.3, 26.5, 26.6, 26.9, 28.8, 32.3, 32.6, 32.7, 32.8, 34.1, 34.2, 37.3, 37.6, 50.7, 65.3, 65.4, 161.0, 170.9, 171.9; IR (neat) ν: 3256, 2917, 1684, 1655, 1538, 1499, 1217; HRMS (ESI) *m*/*z* calcd for $C_{27}H_{42}N_3O_3$ 456.3221; found $[M + H]^+$ 456.3242.

'Isocyano'-Adm-Adm-NH-*i*-Pr (7a)

Isonitrile **7a** was synthesized from dipeptide **6a** (2.4 g, 5.4 mmol) according the procedure described for the synthesis of isocyanide **5a**, and purified by column chromatography using 20% EtOAc in hexanes as eluent. Evaporation of the collected fractions gave 'CN'-Adm-Adm-NH-*i*-Pr

(**7a**, 2.23 g, 97%) as white powder: R_f 0.51 (7:3 hexanes:EtOAc); m.p 228-235 °C; ^{1}H NMR (500 MHz, $CHCl_3$) δ 1.12 (d, *J* = 6.5 Hz, 6H), 1.71-1.72 (m, 7H), 1.77 (m, 3H), 1.80 (m, 4H), 1.86-1.92 (m, 6H), 1.97-2.00 (m, 2H), 2.22-2.25 (m, 2H), 2.31 (m, 2H), 2.72 (m, 2H), 4.02-4.09 (m, 1H), 5.73 (s, 1H), 6.84 (d, *J* = 8.0 Hz, 1H); ^{13}C NMR (500 MHz, $CDCl_3$): δ 22.8, 26.1, 26.2, 26.4, 26.7, 32.4, 32.9, 33.2, 33.4, 34.2, 35.0, 37.1, 37.3, 41.3, 64.8, 69.0, 158.8, 167.2, 170.3; IR (neat) ν: 3357, 2856, 2135, 1677, 1632, 1527, 753. HRMS (ESI) *m/z* calcd for $C_{26}H_{38}N_3O_2$ 424.2959, found $[M + H]^+$ 424.2967.

'Isocyano'-Adm-Adm-NH-*t*-Bu (7b)

Isonitrile **7a** was synthesized from dipeptide **6b** (1.16g, 2.54 mmol) according the procedure described for the synthesis of isocyanide **5a**, and purified by column chromatography using 30% EtOAc in hexanes as eluent. Evaporation of the collected fractions gave 'CN'-Adm-Adm-NH-*i*-Pr (**7b**, 0.62g, 56%) as white powder: R_f 0.67 (7:3 Hex:EtOAc); m.p. 207-210 °C; ^{1}H NMR (500 MHz, $CDCl_3$) δ 1.32 (s, 9H), 1.69-1.72 (m, 7H), 1.77 (m, 3H), 1.79-1.82 (m, 4H), 1.87-1.90 (m, 6H), 1.98-2.00 (m, 2H), 2.22-2.25 (m, 2H), 2.33 (m, 2H), 2.68 (m, 2H), 5.30 (s, 1H), 6.91 (s, 1H); ^{13}C NMR (500 MHz, $CDCl_3$): δ 26.1, 26.2, 26.4, 26.7, 28.8, 32.6, 32.9, 33.2, 33.5, 34.2, 35.0, 37.1, 37.3, 51.0, 65.4, 68.9, 158.6, 167.1, 170.2; IR (neat) ν = 3321, 2919, 2123, 1654, 1537, 1509, 1452; HRMS (ESI) *m/z* calcd for $C_{27}H_{40}N_3O_2$ 438.3115; found $[M + H]^+$ 438.3131.

Formyl-Adm-Adm-Adm-NH-*i*-Pr (1)

Peptide **1** was synthesized from isocyanide **7a** (1.18 g, 2.8 mmol) according to the procedure described for the synthesis of formamide **4a**. After 24 h, solid ammonium formate (1 eq) was freshly added to the reaction mixture, which was worked up after 48 h, when complete disappearance of **7a** was observed by TLC. After evaporation of the volatiles, the residue was purified by column chromatography using 30% EtOAc in hexanes as eluent. Evaporation of the collected fractions gave HCO-Adm-Adm-Adm-NH-*i*-Pr (**1**, 1.06 g, 61%) as white powder: R_f 0.24 (7:3 Hex:EtOAc); m.p.> 250 °C; ^{1}H NMR (500 MHz, $CDCl_3$) δ 1.14 (d, *J* = 6.5 Hz, 6H), 1.63-1.72 (m, 16H), 1.76-1.78 (m, 5H), 1.83 (m, 3H), 1.90-1.93 (m, 4H), 1.95-1.97 (m, 6H), 2.08-2.11 (m,

2H), 2.63-2.66 (m, 6H), 3.91-3.98 (m, 1H), 5.57 (s, 1H), 7.00 (s, 1H), 7.01 (s, 1H), 7.17 (s, 1H), 8.10 (d, $J = 2.0$ Hz, 1H); ^{13}C NMR (quantitative analysis, 700 MHz, $CDCl_3$) δ 22.7 (2C), 26.4, 26.7 (3C), 26.9, 27.0, 32.2 (2C), 32.4 (2C), 32.6 (2C), 32.8 (2C), 33.1 (4C), 34.2 (6C), 37.3, 37.5, 37.7, 41.4, 64.3, 65.5, 65.8, 162.3, 170.9, 172.1, 173.3; IR (neat) ν: 3309, 2909, 2860, 1681, 1647, 1525, 1497, 1470; HRMS (ESI) *m*/*z* calcd for $C_{37}H_{55}N_4O_4$ 619.4218; found $[M + H]^+$ 619.4226.

Formyl-Adm-Adm-Adm-NH-*t*-Bu (**2**)

Peptide **2** was synthesized from isonitrile **7b** (0.6 g, 1.37 mmol) according to a modified procedure as that described for the synthesis of formamide **4a**. The mixture was stirred for 48h. Each day freshly made sat. solution of $NH_4^+HCO_2^-$ (1eq) was added to the mixture. The volatiles were removed after completion of the reaction. The white solid was partitioned between H_2O and CH_2Cl_2. The organic layer was washed with brine and dried over Na_2SO_4. Evaporation of the volatiles gave a white solid which was purified by column chromatography using 30% EtOAc in hexanes as eluent. Evaporation of the collected fractions gave HCO-Adm-Adm-Adm-NH-*t*-Bu (**2**, 0.52, 61%) as white powder: R_f 0.32 (7:3 hexanes:EtOAc); m.p. > 250 °C; ^1H NMR (500 MHz, $CDCl_3$) δ 1.31 (s, 9H), 1.58 (m, 1H), 1.61-1.62 (m, 2H), 1.66-1.69 (m, 12H), 1.73 (m, 2H), 1.77-1.82 (m, 6H), 1.89-1.97 (m, 11H), 2.05-2.08 (m, 2H), 2.63-2.65 (m, 6H), 5.59 (s, 1H), 6.69 (s, 1H), 7.07 (s, 1H), 7.16 (s, 1H), 8.10 (d, $J = 2.0$ Hz, 1H); ^{13}C NMR (700 MHz, $CDCl_3$) δ 26.3, 26.6 (3C), 26.9 (2C), 28.7 (3C), 32.2 (2C), 32.5 (2C), 32.6 (2C), 32.7 (2C), 33.1 (4C), 34.1 (4C), 34.2 (2C), 37.3 , 37.5, 37.7, 50.8, 65.0 (2C), 65.7, 162.1, 170.9, 171.4, 172.9; IR (neat) ν: 2907, 2857, 1673, 1513, 749; HRMS (ESI) *m*/*z* calcd for $C_{38}H_{57}N_4O_4$ 633.4374; found $[M + H]^+$ 633.4394.

X-RAY DIFFRACTION

Tripeptide **1** was crystallized by dissolving 3-4 mg of peptide **1** in 1 mL of acetone and equilibrating hexane vapour into the mother liquor in a closed container to provide slow formation of crystals. Crystals of **1** were also obtained from other solvent systems: acetone with drops of $CHCl_3$, large blocks; acetone, needles; EtOAc/Acetone/$CHCl_3$, needles. Crystals of peptide **2** were

similarly grown from a mixture of acetone/EtOAc and from DMSO. X-Ray diffraction data collection was performed at the Laboratoire de Diffraction des Rayons X de l'Université de Montréal with a Bruker Venture Metaljet diffractometer, equipped with a Gallium Liquid Metal Jet Source (Ga Kα radiation, λ = 1.34139 Å), Helios MX Mirror Optics, a kappa goniometer, and a Photon 100 CMOS detector. Data collection, data reduction, and absorption correction were achieved by use of the APEX 2, SAINT, and SADABS software packages (Bruker AXS). Using Olex2 [S1], the structures were solved with the ShelXT [S2] structure solution program using Intrinsic Phasing, and refined by full-matrix least-squares procedures on F^2, using all data, with the XL [S3] refinement package. CCDC 1906513 – 1906517 contain the supplementary crystallographic data for this paper. The data can be obtained free of charge from The Cambridge Crystallographic Data Centre via www.ccdc.cam.ac.uk/structures.

Peptide 1 (LUB118)

from acetone

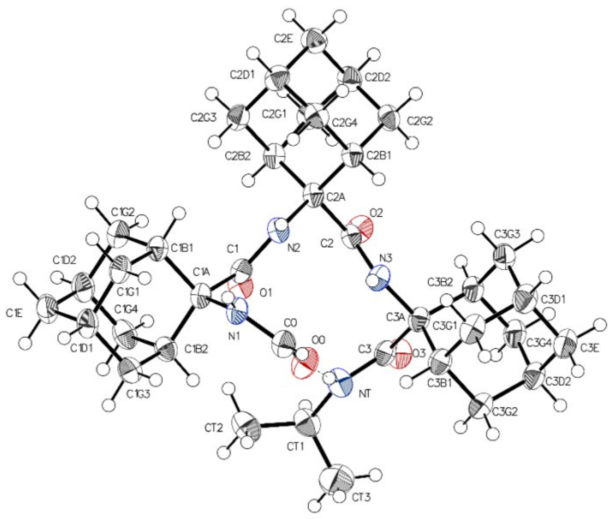

Table 1. Crystal data and structure refinement for lub118.

Identification code	lub118
Empirical formula	$C_{37}H_{54}N_4O_4$
Formula weight	618.84

Temperature/K	150
Crystal system	monoclinic
Space group	$P2_1/c$
a/Å	14.6491(6)
b/Å	14.7140(6)
c/Å	16.1495(7)
α/°	90
β/°	109.618(2)
γ/°	90
Volume/Å^3	3278.9(2)
Z	4
ρ_{calc}g/cm^3	1.254
μ/mm^{-1}	0.413
F(000)	1344.0
Crystal size/mm^3	0.138 × 0.043 × 0.04
Radiation	GaKα (λ = 1.34139)
2Θ range for data collection/°	5.572 to 110.142
Index ranges	$-17 \leq h \leq 17$, $-17 \leq k \leq 17$, $-19 \leq l \leq 19$
Reflections collected	38793
Independent reflections	6204 [R_{int} = 0.0774, R_{sigma} = 0.0613]
Data/restraints/parameters	6204/0/409
Goodness-of-fit on F^2	1.020
Final R indexes [I>=2σ (I)]	R_1 = 0.0577, wR_2 = 0.1226
Final R indexes [all data]	R_1 = 0.1106, wR_2 = 0.1466
Largest diff. peak/hole / e Å^{-3}	0.26/-0.23
CCDC deposition No.	1906513

Table 2. Hydrogen Bonds for lub118.

D H A	d(D-H)/Å	d(H-A)/Å	d(D-A)/Å	D-H-A/°
N1 H1 O2[1]	0.88	1.98	2.745(2)	143.8
N1 H1 O3[1]	0.88	2.61	3.181(3)	123.1
N3 H3 O0	0.88	2.61	3.117(3)	117.4
NT HT O0	0.88	2.22	3.095(3)	177.6

[1] x, 1/2-y, 1/2+z

Table 3. Torsion Angles for lub118.

A	B	C	D	Angle/°	A	B	C	D	Angle/°
C0	N1	C1A	C1B1	-170.2(2)	C2B2	C2A	C2B1	C2G1	60.3(2)
C0	N1	C1A	C1B2	71.7(3)	C2B2	C2A	C2B1	C2G2	-59.4(2)
C0	N1	C1A	C1	-52.4(3)	C2B2	C2A	C2	O2	16.2(3)
O0	C0	N1	C1A	5.2(4)	C2B2	C2A	C2	N3	-169.11(19)
N1	C1A	C1B1	C1G1	-58.6(2)	C2B2	C2G3	C2D1	C2G1	-60.6(2)
N1	C1A	C1B1	C1G2	-178.08(19)	C2B2	C2G3	C2D1	C2E	58.8(3)
N1	C1A	C1B2	C1G3	58.0(2)	C2B2	C2G4	C2D2	C2G2	57.3(3)
N1	C1A	C1B2	C1G4	177.16(19)	C2B2	C2G4	C2D2	C2E	-62.8(3)
N1	C1A	C1	O1	132.8(2)	C2G1	C2B1	C2G2	C2D2	-58.5(2)
N1	C1A	C1	N2	-49.0(3)	C2G1	C2D1	C2E	C2D2	61.5(3)
C1A	C1B1	C1G1	C1D1	-59.0(2)	C2G2	C2B1	C2G1	C2D1	59.7(2)
C1A	C1B1	C1G2	C1D2	62.3(3)	C2G2	C2D2	C2E	C2D1	-60.3(3)
C1A	C1B2	C1G3	C1D1	61.7(2)	C2G3	C2B2	C2G4	C2D2	61.7(2)
C1A	C1B2	C1G4	C1D2	-59.0(3)	C2G3	C2D1	C2E	C2D2	-57.8(3)
C1A	C1	N2	C2A	-169.76(19)	C2G4	C2B2	C2G3	C2D1	-59.6(2)
C1B1	C1A	C1B2	C1G3	-61.0(2)	C2G4	C2D2	C2E	C2D1	59.8(3)
C1B1	C1A	C1B2	C1G4	58.2(2)	C2	C2A	C2B1	C2G1	-176.76(19)
C1B1	C1A	C1	O1	-109.3(3)	C2	C2A	C2B1	C2G2	63.6(2)
C1B1	C1A	C1	N2	68.9(2)	C2	C2A	C2B2	C2G3	179.30(19)
C1B1	C1G1	C1D1	C1G3	57.7(3)	C2	C2A	C2B2	C2G4	-62.4(2)
C1B1	C1G1	C1D1	C1E	-62.4(3)	C2	N3	C3A	C3B1	-178.1(2)
C1B1	C1G2	C1D2	C1G4	-61.9(3)	C2	N3	C3A	C3B2	64.2(3)
C1B1	C1G2	C1D2	C1E	58.3(3)	C2	N3	C3A	C3	-59.7(3)
C1B2	C1A	C1B1	C1G1	60.7(2)	O2	C2	N3	C3A	-2.3(3)
C1B2	C1A	C1B1	C1G2	-58.8(2)	N3	C3A	C3B1	C3G1	-61.7(2)
C1B2	C1A	C1	O1	11.5(3)	N3	C3A	C3B1	C3G2	178.84(19)
C1B2	C1A	C1	N2	-170.23(19)	N3	C3A	C3B2	C3G3	58.4(3)
C1B2	C1G3	C1D1	C1G1	-60.1(3)	N3	C3A	C3B2	C3G4	177.59(18)
C1B2	C1G3	C1D1	C1E	59.7(3)	N3	C3A	C3	O3	121.5(2)
C1B2	C1G4	C1D2	C1G2	59.2(3)	N3	C3A	C3	NT	-59.6(3)
C1B2	C1G4	C1D2	C1E	-61.3(3)	C3A	C3B1	C3G1	C3D1	-60.5(3)
C1G1	C1B1	C1G2	C1D2	-58.5(3)	C3A	C3B1	C3G2	C3D2	62.0(3)
C1G1	C1D1	C1E	C1D2	61.0(3)	C3A	C3B2	C3G3	C3D1	62.3(3)
C1G2	C1B1	C1G1	C1D1	60.7(3)	C3A	C3B2	C3G4	C3D2	-59.8(3)
C1G2	C1D2	C1E	C1D1	-59.0(3)	C3A	C3	NT	CT1	-172.4(2)
C1G3	C1B2	C1G4	C1D2	60.1(3)	C3B1	C3A	C3B2	C3G3	-59.7(2)
C1G3	C1D1	C1E	C1D2	-59.3(3)	C3B1	C3A	C3B2	C3G4	59.5(2)
C1G4	C1B2	C1G3	C1D1	-59.6(3)	C3B1	C3A	C3	O3	-121.4(2)

C1G4	C1D2	C1E	C1D1	60.4(3)	C3B1	C3A	C3	NT	57.5(3)
C1	C1A	C1B1	C1G1	-174.75(18)	C3B1	C3G1	C3D1	C3G3	59.7(3)
C1	C1A	C1B1	C1G2	65.7(2)	C3B1	C3G1	C3D1	C3E	-60.8(3)
C1	C1A	C1B2	C1G3	177.53(19)	C3B1	C3G2	C3D2	C3G4	-60.4(3)
C1	C1A	C1B2	C1G4	-63.3(3)	C3B1	C3G2	C3D2	C3E	59.7(3)
C1	N2	C2A	C2B1	-177.24(19)	C3B2	C3A	C3B1	C3G1	59.2(2)
C1	N2	C2A	C2B2	64.8(3)	C3B2	C3A	C3B1	C3G2	-60.3(2)
C1	N2	C2A	C2	-59.3(3)	C3B2	C3A	C3	O3	-2.4(3)
O1	C1	N2	C2A	8.6(3)	C3B2	C3A	C3	NT	176.5(2)
N2	C2A	C2B1	C2G1	-59.6(2)	C3B2	C3G3	C3D1	C3G1	-60.8(3)
N2	C2A	C2B1	C2G2	-179.28(18)	C3B2	C3G3	C3D1	C3E	58.6(3)
N2	C2A	C2B2	C2G3	58.3(2)	C3B2	C3G4	C3D2	C3G2	58.6(3)
N2	C2A	C2B2	C2G4	176.62(18)	C3B2	C3G4	C3D2	C3E	-61.5(3)
N2	C2A	C2	O2	138.8(2)	C3G1	C3B1	C3G2	C3D2	-58.6(3)
N2	C2A	C2	N3	-46.5(2)	C3G1	C3D1	C3E	C3D2	60.7(3)
C2A	C2B1	C2G1	C2D1	-60.2(3)	C3G2	C3B1	C3G1	C3D1	59.4(3)
C2A	C2B1	C2G2	C2D2	62.4(2)	C3G2	C3D2	C3E	C3D1	-60.2(3)
C2A	C2B2	C2G3	C2D1	61.9(2)	C3G3	C3B2	C3G4	C3D2	60.3(3)
C2A	C2B2	C2G4	C2D2	-57.5(3)	C3G3	C3D1	C3E	C3D2	-58.2(3)
C2A	C2	N3	C3A	-177.04(19)	C3G4	C3B2	C3G3	C3D1	-58.5(3)
C2B1	C2A	C2B2	C2G3	-60.3(2)	C3G4	C3D2	C3E	C3D1	59.4(3)
C2B1	C2A	C2B2	C2G4	58.0(2)	C3	C3A	C3B1	C3G1	-178.82(19)
C2B1	C2A	C2	O2	-103.4(2)	C3	C3A	C3B1	C3G2	61.7(2)
C2B1	C2A	C2	N3	71.3(2)	C3	C3A	C3B2	C3G3	179.8(2)
C2B1	C2G1	C2D1	C2G3	58.7(3)	C3	C3A	C3B2	C3G4	-61.0(3)
C2B1	C2G1	C2D1	C2E	-61.4(3)	C3	NT	CT1	CT2	-153.7(2)
C2B1	C2G2	C2D2	C2G4	-60.8(2)	C3	NT	CT1	CT3	82.0(3)
C2B1	C2G2	C2D2	C2E	59.1(3)	O3	C3	NT	CT1	6.6(4)

Peptide 1 from acetone / $CHCl_3$

(LUB133)

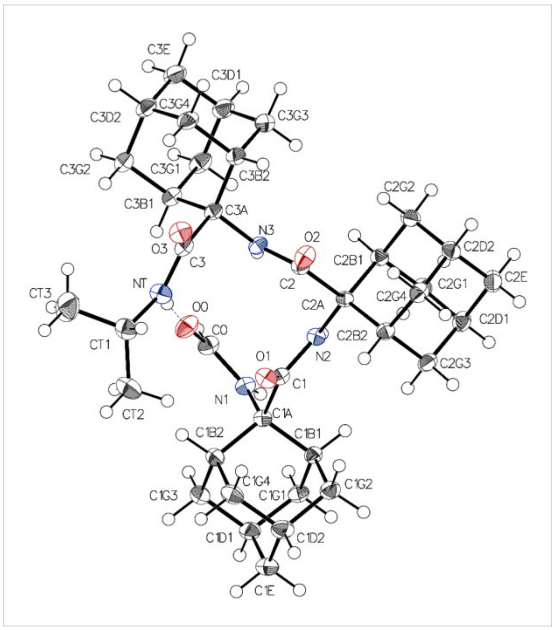

Table 4. Crystal data and structure refinement for lub133.

Identification code	lub133
Empirical formula	$C_{37}H_{54}N_4O_4$
Formula weight	618.84
Temperature/K	150
Crystal system	monoclinic
Space group	$P2_1/c$
a/Å	14.6489(4)
b/Å	14.7284(4)
c/Å	16.1631(4)
α/°	90
β/°	109.7380(10)
γ/°	90
Volume/Å^3	3282.38(15)
Z	4
ρ_{calc}g/cm^3	1.252
μ/mm^{-1}	0.410
F(000)	1344.0
Crystal size/mm^3	0.19 × 0.19 × 0.11
Radiation	GaKα (λ = 1.34139)
2Θ range for data collection/°	5.576 to 121.43
Index ranges	$-19 \le h \le 19$, $-19 \le k \le 19$, $-21 \le l \le 20$

Reflections collected	49304
Independent reflections	7528 [R_{int} = 0.0317, R_{sigma} = 0.0191]
Data/restraints/parameters	7528/0/409
Goodness-of-fit on F^2	1.043
Final R indexes [I>=2σ (I)]	R_1 = 0.0454, wR_2 = 0.1159
Final R indexes [all data]	R_1 = 0.0499, wR_2 = 0.1205
Largest diff. peak/hole / e Å^{-3}	0.38/-0.36
CCDC deposition No.	1906514

Table 5. Hydrogen Bonds for lub133.

D	H	A	d(D-H)/Å	d(H-A)/Å	d(D-A)/Å	D-H-A/°
N1	H1	O2[1]	0.88	1.99	2.7452(12)	143.1
N1	H1	O3[1]	0.88	2.60	3.1790(13)	123.8
N3	H3	O0	0.88	2.61	3.1150(13)	117.6
NT	HT	O0	0.88	2.22	3.0958(13)	177.0

[1] x, 1/2-y, 1/2+z

Table 6. Torsion Angles for lub133.

A	B	C	D	Angle/˚	A	B	C	D	Angle/˚
C0	N1	C1A	C1B1	-170.36(10)	C2B2	C2A	C2B1	C2G1	60.50(11)
C0	N1	C1A	C1B2	71.46(13)	C2B2	C2A	C2B1	C2G2	-59.03(10)
C0	N1	C1A	C1	-52.75(13)	C2B2	C2A	C2	O2	16.22(14)
O0	C0	N1	C1A	5.58(19)	C2B2	C2A	C2	N3	-168.87(9)
N1	C1A	C1B1	C1G1	-58.62(11)	C2B2	C2G3	C2D1	C2G1	-60.09(12)
N1	C1A	C1B1	C1G2	-178.16(9)	C2B2	C2G3	C2D1	C2E	59.09(12)
N1	C1A	C1B2	C1G3	57.91(11)	C2B2	C2G4	C2D2	C2G2	57.37(11)
N1	C1A	C1B2	C1G4	176.93(9)	C2B2	C2G4	C2D2	C2E	-62.94(12)
N1	C1A	C1	O1	133.05(11)	C2G1	C2B1	C2G2	C2D2	-58.30(11)
N1	C1A	C1	N2	-48.55(11)	C2G1	C2D1	C2E	C2D2	61.53(12)
C1A	C1B1	C1G1	C1D1	-59.17(11)	C2G2	C2B1	C2G1	C2D1	59.84(11)
C1A	C1B1	C1G2	C1D2	62.31(12)	C2G2	C2D2	C2E	C2D1	-60.33(12)
C1A	C1B2	C1G3	C1D1	61.66(12)	C2G3	C2B2	C2G4	C2D2	61.82(11)
C1A	C1B2	C1G4	C1D2	-58.83(12)	C2G3	C2D1	C2E	C2D2	-58.02(12)
C1A	C1	N2	C2A	-169.77(9)	C2G4	C2B2	C2G3	C2D1	-59.91(11)
C1B1	C1A	C1B2	C1G3	-61.06(11)	C2G4	C2D2	C2E	C2D1	59.92(12)
C1B1	C1A	C1B2	C1G4	57.95(11)	C2	C2A	C2B1	C2G1	-176.81(9)
C1B1	C1A	C1	O1	-109.16(12)	C2	C2A	C2B1	C2G2	63.66(11)
C1B1	C1A	C1	N2	69.24(11)	C2	C2A	C2B2	C2G3	179.47(9)
C1B1	C1G1	C1D1	C1G3	57.83(12)	C2	C2A	C2B2	C2G4	-62.26(11)
C1B1	C1G1	C1D1	C1E	-62.06(12)	C2	N3	C3A	C3B1	-178.27(9)
C1B1	C1G2	C1D2	C1G4	-61.88(12)	C2	N3	C3A	C3B2	63.83(13)
C1B1	C1G2	C1D2	C1E	58.10(12)	C2	N3	C3A	C3	-59.51(13)

C1B2	C1A	C1B1	C1G1	60.78(11)	O2	C2	N3	C3A	-2.41(16)
C1B2	C1A	C1B1	C1G2	-58.75(11)	N3	C3A	C3B1	C3G1	-61.43(11)
C1B2	C1A	C1	O1	11.47(15)	N3	C3A	C3B1	C3G2	178.74(9)
C1B2	C1A	C1	N2	-170.13(9)	N3	C3A	C3B2	C3G3	58.64(12)
C1B2	C1G3	C1D1	C1G1	-59.85(12)	N3	C3A	C3B2	C3G4	177.52(9)
C1B2	C1G3	C1D1	C1E	59.81(12)	N3	C3A	C3	O3	121.85(11)
C1B2	C1G4	C1D2	C1G2	58.94(12)	N3	C3A	C3	NT	-60.26(12)
C1B2	C1G4	C1D2	C1E	-61.47(12)	C3A	C3B1	C3G1	C3D1	-60.33(12)
C1G1	C1B1	C1G2	C1D2	-58.29(12)	C3A	C3B1	C3G2	C3D2	62.14(12)
C1G1	C1D1	C1E	C1D2	60.70(13)	C3A	C3B2	C3G3	C3D1	61.54(12)
C1G2	C1B1	C1G1	C1D1	60.55(12)	C3A	C3B2	C3G4	C3D2	-59.29(12)
C1G2	C1D2	C1E	C1D1	-58.88(13)	C3A	C3	NT	CT1	-172.63(10)
C1G3	C1B2	C1G4	C1D2	60.32(12)	C3B1	C3A	C3B2	C3G3	-59.54(11)
C1G3	C1D1	C1E	C1D2	-59.43(13)	C3B1	C3A	C3B2	C3G4	59.33(12)
C1G4	C1B2	C1G3	C1D1	-59.76(12)	C3B1	C3A	C3	O3	-120.78(11)
C1G4	C1D2	C1E	C1D1	60.51(13)	C3B1	C3A	C3	NT	57.10(12)
C1	C1A	C1B1	C1G1	-174.85(9)	C3B1	C3G1	C3D1	C3G3	59.35(12)
C1	C1A	C1B1	C1G2	65.61(11)	C3B1	C3G1	C3D1	C3E	-61.22(13)
C1	C1A	C1B2	C1G3	177.74(9)	C3B1	C3G2	C3D2	C3G4	-60.19(12)
C1	C1A	C1B2	C1G4	-63.24(12)	C3B1	C3G2	C3D2	C3E	59.72(12)
C1	N2	C2A	C2B1	-177.23(9)	C3B2	C3A	C3B1	C3G1	59.29(11)
C1	N2	C2A	C2B2	64.61(12)	C3B2	C3A	C3B1	C3G2	-60.54(11)
C1	N2	C2A	C2	-59.59(12)	C3B2	C3A	C3	O3	-1.46(15)
O1	C1	N2	C2A	8.68(16)	C3B2	C3A	C3	NT	176.43(9)
N2	C2A	C2B1	C2G1	-59.67(11)	C3B2	C3G3	C3D1	C3G1	-60.32(12)
N2	C2A	C2B1	C2G2	-179.20(8)	C3B2	C3G3	C3D1	C3E	59.28(13)
N2	C2A	C2B2	C2G3	58.25(11)	C3B2	C3G4	C3D2	C3G2	58.37(12)
N2	C2A	C2B2	C2G4	176.52(9)	C3B2	C3G4	C3D2	C3E	-61.76(12)
N2	C2A	C2	O2	139.12(10)	C3G1	C3B1	C3G2	C3D2	-58.86(12)
N2	C2A	C2	N3	-45.96(12)	C3G1	C3D1	C3E	C3D2	60.84(13)
C2A	C2B1	C2G1	C2D1	-59.96(12)	C3G2	C3B1	C3G1	C3D1	59.92(12)
C2A	C2B1	C2G2	C2D2	62.30(11)	C3G2	C3D2	C3E	C3D1	-60.29(13)
C2A	C2B2	C2G3	C2D1	61.62(11)	C3G3	C3B2	C3G4	C3D2	60.79(12)
C2A	C2B2	C2G4	C2D2	-57.39(12)	C3G3	C3D1	C3E	C3D2	-58.52(13)
C2A	C2	N3	C3A	-177.35(9)	C3G4	C3B2	C3G3	C3D1	-59.13(12)
C2B1	C2A	C2B2	C2G3	-60.56(10)	C3G4	C3D2	C3E	C3D1	59.44(13)
C2B1	C2A	C2B2	C2G4	57.71(11)	C3	C3A	C3B1	C3G1	-178.68(9)
C2B1	C2A	C2	O2	-103.17(11)	C3	C3A	C3B1	C3G2	61.48(11)
C2B1	C2A	C2	N3	71.74(11)	C3	C3A	C3B2	C3G3	179.59(9)
C2B1	C2G1	C2D1	C2G3	58.46(11)	C3	C3A	C3B2	C3G4	-61.53(12)
C2B1	C2G1	C2D1	C2E	-61.66(12)	C3	NT	CT1	CT2	-153.50(12)
C2B1	C2G2	C2D2	C2G4	-61.01(12)	C3	NT	CT1	CT3	82.71(16)
C2B1	C2G2	C2D2	C2E	59.10(12)	O3	C3	NT	CT1	5.27(18)

Peptide 1 from acetone / EtOAc / $CHCl_3$

[LUB133(3)]

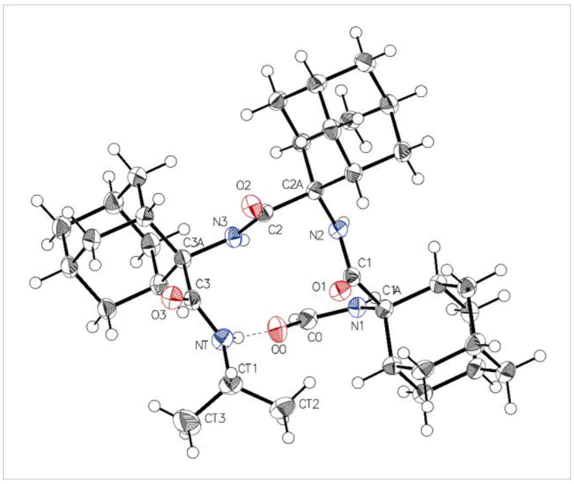

Table 7. Crystal data and structure refinement for lub133(3).

Identification code	lub133(3)
Empirical formula	$C_{37}H_{54}N_4O_4$
Formula weight	618.84
Temperature/K	150
Crystal system	monoclinic
Space group	$P2_1/c$
a/Å	14.6463(6)
b/Å	14.7404(6)
c/Å	16.1538(7)
α/°	90
β/°	109.735(2)
γ/°	90
Volume/Å^3	3282.6(2)
Z	4
ρ_{calc}g/cm^3	1.252
μ/mm^{-1}	0.410
F(000)	1344.0
Crystal size/mm^3	0.35 × 0.06 × 0.03
Radiation	GaKα (λ = 1.34139)
2Θ range for data collection/°	5.578 to 114.496
Index ranges	$-17 \le h \le 18$, $-18 \le k \le 18$, $-18 \le l \le 18$
Reflections collected	31865
Independent reflections	6452 [R_{int} = 0.0454, R_{sigma} = 0.0341]
Data/restraints/parameters	6452/0/409
Goodness-of-fit on F^2	1.057

Final R indexes [I>=2σ (I)]	R_1 = 0.0469, wR_2 = 0.1017
Final R indexes [all data]	R_1 = 0.0655, wR_2 = 0.1134
Largest diff. peak/hole / e Å^{-3}	0.27/-0.33
CCDC deposition No.	1906515

Table 8. Hydrogen Bonds for lub133(3).

D	H	A	d(D-H)/Å	d(H-A)/Å	d(D-A)/Å	D-H-A/°
N1	H1	O2[1]	0.88	1.99	2.7448(18)	143.2
N1	H1	O3[1]	0.88	2.61	3.1839(19)	123.8
N3	H3	O0	0.88	2.61	3.1147(19)	117.7
NT	HT	O0	0.88	2.21	3.094(2)	177.2

[1] x, 1/2-y, 1/2+z

Table 9. Torsion Angles for lub133(3).

A	B	C	D	Angle/°	A	B	C	D	Angle/°
C0	N1	C1A	C1B1	-170.35(15)	C2B2	C2A	C2B1	C2G1	60.56(17)
C0	N1	C1A	C1B2	71.64(19)	C2B2	C2A	C2B1	C2G2	-59.24(16)
C0	N1	C1A	C1	-52.77(19)	C2B2	C2A	C2	O2	16.2(2)
O0	C0	N1	C1A	5.4(3)	C2B2	C2A	C2	N3	-168.90(13)
N1	C1A	C1B1	C1G1	-58.55(17)	C2B2	C2G3	C2D1	C2G1	-60.18(18)
N1	C1A	C1B1	C1G2	-178.10(13)	C2B2	C2G3	C2D1	C2E	59.32(18)
N1	C1A	C1B2	C1G3	57.78(17)	C2B2	C2G4	C2D2	C2G2	57.25(18)
N1	C1A	C1B2	C1G4	177.14(13)	C2B2	C2G4	C2D2	C2E	-63.01(17)
N1	C1A	C1	O1	133.00(16)	C2G1	C2B1	C2G2	C2D2	-58.42(17)
N1	C1A	C1	N2	-48.77(17)	C2G1	C2D1	C2E	C2D2	61.49(18)
C1A	C1B1	C1G1	C1D1	-59.25(17)	C2G2	C2B1	C2G1	C2D1	59.79(17)
C1A	C1B1	C1G2	C1D2	62.23(18)	C2G2	C2D2	C2E	C2D1	-60.32(18)
C1A	C1B2	C1G3	C1D1	61.87(17)	C2G3	C2B2	C2G4	C2D2	61.98(17)
C1A	C1B2	C1G4	C1D2	-58.84(18)	C2G3	C2D1	C2E	C2D2	-58.17(18)
C1A	C1	N2	C2A	-169.85(13)	C2G4	C2B2	C2G3	C2D1	-59.95(17)
C1B1	C1A	C1B2	C1G3	-61.28(17)	C2G4	C2D2	C2E	C2D1	59.94(18)
C1B1	C1A	C1B2	C1G4	58.08(18)	C2	C2A	C2B1	C2G1	-176.72(13)
C1B1	C1A	C1	O1	-109.08(17)	C2	C2A	C2B1	C2G2	63.48(16)
C1B1	C1A	C1	N2	69.14(17)	C2	C2A	C2B2	C2G3	179.43(13)
C1B1	C1G1	C1D1	C1G3	57.94(18)	C2	C2A	C2B2	C2G4	-62.28(17)
C1B1	C1G1	C1D1	C1E	-62.17(17)	C2	N3	C3A	C3B1	-178.39(14)
C1B1	C1G2	C1D2	C1G4	-61.66(19)	C2	N3	C3A	C3B2	63.84(19)
C1B1	C1G2	C1D2	C1E	58.28(18)	C2	N3	C3A	C3	-59.58(19)
C1B2	C1A	C1B1	C1G1	60.79(17)	O2	C2	N3	C3A	-2.4(2)
C1B2	C1A	C1B1	C1G2	-58.76(17)	N3	C3A	C3B1	C3G1	-61.40(17)

C1B2	C1A	C1	O1	11.4(2)
C1B2	C1A	C1	N2	-170.33(13)
C1B2	C1G3	C1D1	C1G1	-59.91(19)
C1B2	C1G3	C1D1	C1E	59.81(18)
C1B2	C1G4	C1D2	C1G2	58.78(19)
C1B2	C1G4	C1D2	C1E	-61.62(18)
C1G1	C1B1	C1G2	C1D2	-58.41(18)
C1G1	C1D1	C1E	C1D2	60.89(19)
C1G2	C1B1	C1G1	C1D1	60.56(17)
C1G2	C1D2	C1E	C1D1	-59.15(19)
C1G3	C1B2	C1G4	C1D2	60.76(18)
C1G3	C1D1	C1E	C1D2	-59.37(19)
C1G4	C1B2	C1G3	C1D1	-59.91(18)
C1G4	C1D2	C1E	C1D1	60.39(19)
C1	C1A	C1B1	C1G1	-174.64(13)
C1	C1A	C1B1	C1G2	65.81(16)
C1	C1A	C1B2	C1G3	177.51(13)
C1	C1A	C1B2	C1G4	-63.13(18)
C1	N2	C2A	C2B1	-177.32(13)
C1	N2	C2A	C2B2	64.82(18)
C1	N2	C2A	C2	-59.46(18)
O1	C1	N2	C2A	8.4(2)
N2	C2A	C2B1	C2G1	-59.47(17)
N2	C2A	C2B1	C2G2	-179.26(12)
N2	C2A	C2B2	C2G3	58.18(17)
N2	C2A	C2B2	C2G4	176.47(13)
N2	C2A	C2	O2	139.09(15)
N2	C2A	C2	N3	-45.97(17)
C2A	C2B1	C2G1	C2D1	-60.23(18)
C2A	C2B1	C2G2	C2D2	62.47(17)
C2A	C2B2	C2G3	C2D1	61.71(17)
C2A	C2B2	C2G4	C2D2	-57.36(18)
C2A	C2	N3	C3A	-177.35(13)
C2B1	C2A	C2B2	C2G3	-60.53(16)
C2B1	C2A	C2B2	C2G4	57.76(17)
C2B1	C2A	C2	O2	-103.06(17)
C2B1	C2A	C2	N3	71.87(17)
C2B1	C2G1	C2D1	C2G3	58.64(18)
C2B1	C2G1	C2D1	C2E	-61.67(17)
C2B1	C2G2	C2D2	C2G4	-60.90(18)
C2B1	C2G2	C2D2	C2E	59.11(18)

N3	C3A	C3B1	C3G2	178.76(13)
N3	C3A	C3B2	C3G3	58.53(17)
N3	C3A	C3B2	C3G4	177.54(14)
N3	C3A	C3	O3	121.89(16)
N3	C3A	C3	NT	-60.22(18)
C3A	C3B1	C3G1	C3D1	-60.35(18)
C3A	C3B1	C3G2	C3D2	62.25(18)
C3A	C3B2	C3G3	C3D1	61.88(18)
C3A	C3B2	C3G4	C3D2	-59.36(18)
C3A	C3	NT	CT1	-172.47(15)
C3B1	C3A	C3B2	C3G3	-59.67(17)
C3B1	C3A	C3B2	C3G4	59.34(18)
C3B1	C3A	C3	O3	-120.65(17)
C3B1	C3A	C3	NT	57.24(18)
C3B1	C3G1	C3D1	C3G3	59.42(19)
C3B1	C3G1	C3D1	C3E	-61.25(19)
C3B1	C3G2	C3D2	C3G4	-60.22(18)
C3B1	C3G2	C3D2	C3E	59.69(18)
C3B2	C3A	C3B1	C3G1	59.26(17)
C3B2	C3A	C3B1	C3G2	-60.58(17)
C3B2	C3A	C3	O3	-1.4(2)
C3B2	C3A	C3	NT	176.48(14)
C3B2	C3G3	C3D1	C3G1	-60.51(19)
C3B2	C3G3	C3D1	C3E	58.96(19)
C3B2	C3G4	C3D2	C3G2	58.42(18)
C3B2	C3G4	C3D2	C3E	-61.78(18)
C3G1	C3B1	C3G2	C3D2	-58.80(18)
C3G1	C3D1	C3E	C3D2	60.9(2)
C3G2	C3B1	C3G1	C3D1	59.95(18)
C3G2	C3D2	C3E	C3D1	-60.37(19)
C3G3	C3B2	C3G4	C3D2	60.79(18)
C3G3	C3D1	C3E	C3D2	-58.4(2)
C3G4	C3B2	C3G3	C3D1	-58.87(18)
C3G4	C3D2	C3E	C3D1	59.35(19)
C3	C3A	C3B1	C3G1	-178.65(13)
C3	C3A	C3B1	C3G2	61.51(17)
C3	C3A	C3B2	C3G3	179.45(13)
C3	C3A	C3B2	C3G4	-61.55(18)
C3	NT	CT1	CT2	-153.65(17)
C3	NT	CT1	CT3	82.7(2)
O3	C3	NT	CT1	5.4(3)

Peptide 2 (lub132)

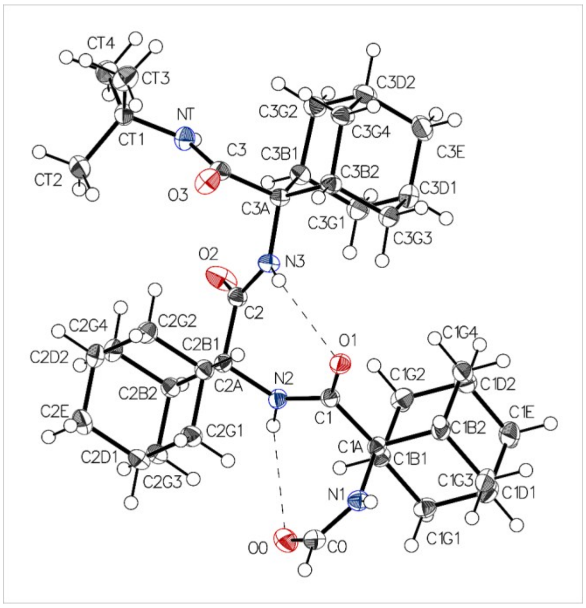

Table 10. Crystal data and structure refinement for lub132.

Identification code	lub132
Empirical formula	$C_{38}H_{56}N_4O_4$
Formula weight	632.86
Temperature/K	150
Crystal system	Triclinic
Space group	P-1
a/Å	7.7427(3)
b/Å	10.1000(3)
c/Å	20.7549(7)
α/°	90.0300(10)
β/°	95.7130(10)
γ/°	90.8690(10)
Volume/Å^3	1614.81(10)
Z	2
ρ_{calc}g/cm^3	1.302
μ/mm^{-1}	0.427
F(000)	688.0
Crystal size/mm^3	0.15 × 0.12 × 0.08
Radiation	GaKα (λ = 1.34139)
2Θ range for data collection/°	3.722 to 121.502
Index ranges	$-10 \le h \le 10$, $-13 \le k \le 13$, $-27 \le l \le 26$
Reflections collected	42140
Independent reflections	7412 [R_{int} = 0.0354, R_{sigma} = 0.0242]
Data/restraints/parameters	7412/0/435
Goodness-of-fit on F^2	1.071
Final R indexes [I>=2σ (I)]	R_1 = 0.0495, wR_2 = 0.1279
Final R indexes [all data]	R_1 = 0.0543, wR_2 = 0.1319

Largest diff. peak/hole / e Å^{-3} 0.42/-0.41
CCDC deposition No. 1906516

Table 11. Hydrogen Bonds for lub132.

D	H	A	d(D-H)/Å	d(H-A)/Å	d(D-A)/Å	D-H-A/°
N1	H1	O2[1]	0.95(2)	1.99(2)	2.9161(15)	164.6(17)
C1B1	H1B1	O0	1.00	2.37	2.9486(16)	116.2
C1B2	H1B2	O2[1]	1.00	2.66	3.4311(18)	134.4
N2	H2	O0	0.833(19)	2.572(18)	3.1665(15)	129.4(15)
C2B1	H2B1	O1	1.00	2.41	3.0250(15)	119.2
N3	H3	O1	0.86(2)	2.31(2)	2.8804(14)	124.5(16)

[1] -1+x, y, z

Table 12. Torsion Angles for lub132.

A	B	C	D	Angle/˚	A	B	C	D	Angle/˚
C0	N1	C1A	C1B1	-42.33(17)	C2B2	C2A	C2B1	C2G2	-60.77(13)
C0	N1	C1A	C1B2	-161.01(12)	C2B2	C2A	C2	O2	-20.05(16)
C0	N1	C1A	C1	82.12(15)	C2B2	C2A	C2	N3	161.76(11)
O0	C0	N1	C1A	6.7(2)	C2B2	C2G3	C2D1	C2G1	-59.47(14)
N1	C1A	C1B1	C1G1	-58.16(14)	C2B2	C2G3	C2D1	C2E	60.49(14)
N1	C1A	C1B1	C1G2	-178.06(10)	C2B2	C2G4	C2D2	C2G2	59.44(14)
N1	C1A	C1B2	C1G3	62.11(13)	C2B2	C2G4	C2D2	C2E	-60.74(14)
N1	C1A	C1B2	C1G4	-177.93(10)	C2G1	C2B1	C2G2	C2D2	-59.14(14)
N1	C1A	C1	O1	92.17(13)	C2G1	C2D1	C2E	C2D2	59.71(16)
N1	C1A	C1	N2	-84.06(12)	C2G2	C2B1	C2G1	C2D1	58.78(14)
C1A	C1B1	C1G1	C1D1	-60.55(14)	C2G2	C2D2	C2E	C2D1	-60.15(15)
C1A	C1B1	C1G2	C1D2	61.63(14)	C2G3	C2B2	C2G4	C2D2	60.30(14)
C1A	C1B2	C1G3	C1D1	60.84(14)	C2G3	C2D1	C2E	C2D2	-59.62(15)
C1A	C1B2	C1G4	C1D2	-60.64(14)	C2G4	C2B2	C2G3	C2D1	-60.20(14)
C1A	C1	N2	C2A	171.96(10)	C2G4	C2D2	C2E	C2D1	59.67(16)
C1B1	C1A	C1B2	C1G3	-60.20(13)	C2	C2A	C2B1	C2G1	179.85(10)
C1B1	C1A	C1B2	C1G4	59.76(13)	C2	C2A	C2B1	C2G2	60.07(13)
C1B1	C1A	C1	O1	-143.84(12)	C2	C2A	C2B2	C2G3	176.77(10)
C1B1	C1A	C1	N2	39.93(15)	C2	C2A	C2B2	C2G4	-63.67(13)
C1B1	C1G1	C1D1	C1G3	59.56(14)	C2	N3	C3A	C3B1	-47.42(17)
C1B1	C1G1	C1D1	C1E	-60.61(14)	C2	N3	C3A	C3B2	-166.18(12)
C1B1	C1G2	C1D2	C1G4	-60.39(14)	C2	N3	C3A	C3	80.34(15)
C1B1	C1G2	C1D2	C1E	59.26(14)	O2	C2	N3	C3A	13.3(2)
C1B2	C1A	C1B1	C1G1	59.89(13)	N3	C3A	C3B1	C3G1	-57.68(13)
C1B2	C1A	C1B1	C1G2	-60.01(13)	N3	C3A	C3B1	C3G2	-176.40(10)

C1B2	C1A	C1	O1	-21.92(16)	N3	C3A	C3B2	C3G3	61.30(13)
C1B2	C1A	C1	N2	161.85(11)	N3	C3A	C3B2	C3G4	-178.56(10)
C1B2	C1G3	C1D1	C1G1	-59.56(15)	N3	C3A	C3	O3	65.25(14)
C1B2	C1G3	C1D1	C1E	60.04(14)	N3	C3A	C3	NT	-113.17(12)
C1B2	C1G4	C1D2	C1G2	59.82(14)	C3A	C3B1	C3G1	C3D1	-61.27(14)
C1B2	C1G4	C1D2	C1E	-59.67(15)	C3A	C3B1	C3G2	C3D2	58.85(14)
C1G1	C1B1	C1G2	C1D2	-58.74(14)	C3A	C3B2	C3G3	C3D1	59.64(14)
C1G1	C1D1	C1E	C1D2	60.46(15)	C3A	C3B2	C3G4	C3D2	-63.13(14)
C1G2	C1B1	C1G1	C1D1	59.58(14)	C3A	C3	NT	CT1	174.19(11)
C1G2	C1D2	C1E	C1D1	-60.03(15)	C3B1	C3A	C3B2	C3G3	-60.70(13)
C1G3	C1B2	C1G4	C1D2	59.93(14)	C3B1	C3A	C3B2	C3G4	59.44(13)
C1G3	C1D1	C1E	C1D2	-59.14(15)	C3B1	C3A	C3	O3	-169.83(12)
C1G4	C1B2	C1G3	C1D1	-59.93(14)	C3B1	C3A	C3	NT	11.74(16)
C1G4	C1D2	C1E	C1D1	58.72(15)	C3B1	C3G1	C3D1	C3G3	59.53(14)
C1	C1A	C1B1	C1G1	-176.66(10)	C3B1	C3G1	C3D1	C3E	-59.59(15)
C1	C1A	C1B1	C1G2	63.45(13)	C3B1	C3G2	C3D2	C3G4	-59.11(14)
C1	C1A	C1B2	C1G3	174.20(10)	C3B1	C3G2	C3D2	C3E	61.21(14)
C1	C1A	C1B2	C1G4	-65.84(13)	C3B2	C3A	C3B1	C3G1	60.68(13)
C1	N2	C2A	C2B1	-51.10(16)	C3B2	C3A	C3B1	C3G2	-58.05(13)
C1	N2	C2A	C2B2	-169.21(11)	C3B2	C3A	C3	O3	-47.88(15)
C1	N2	C2A	C2	74.34(14)	C3B2	C3A	C3	NT	133.69(12)
O1	C1	N2	C2A	-4.1(2)	C3B2	C3G3	C3D1	C3G1	-57.95(14)
N2	C2A	C2B1	C2G1	-59.39(13)	C3B2	C3G3	C3D1	C3E	61.96(14)
N2	C2A	C2B1	C2G2	-179.17(10)	C3B2	C3G4	C3D2	C3G2	61.99(14)
N2	C2A	C2B2	C2G3	61.96(13)	C3B2	C3G4	C3D2	C3E	-57.73(15)
N2	C2A	C2B2	C2G4	-178.48(10)	C3G1	C3B1	C3G2	C3D2	-60.33(14)
N2	C2A	C2	O2	95.94(14)	C3G1	C3D1	C3E	C3D2	58.92(15)
N2	C2A	C2	N3	-82.25(13)	C3G2	C3B1	C3G1	C3D1	59.45(14)
C2A	C2B1	C2G1	C2D1	-61.33(14)	C3G2	C3D2	C3E	C3D1	-59.72(15)
C2A	C2B1	C2G2	C2D2	61.22(14)	C3G3	C3B2	C3G4	C3D2	57.92(14)
C2A	C2B2	C2G3	C2D1	60.20(14)	C3G3	C3D1	C3E	C3D2	-60.73(15)
C2A	C2B2	C2G4	C2D2	-60.92(14)	C3G4	C3B2	C3G3	C3D1	-60.41(14)
C2A	C2	N3	C3A	-168.55(11)	C3G4	C3D2	C3E	C3D1	59.09(15)
C2B1	C2A	C2B2	C2G3	-58.84(13)	C3	C3A	C3B1	C3G1	-177.49(10)
C2B1	C2A	C2B2	C2G4	60.73(13)	C3	C3A	C3B1	C3G2	63.78(14)
C2B1	C2A	C2	O2	-140.25(13)	C3	C3A	C3B2	C3G3	171.63(10)
C2B1	C2A	C2	N3	41.56(15)	C3	C3A	C3B2	C3G4	-68.23(13)
C2B1	C2G1	C2D1	C2G3	60.56(14)	C3	NT	CT1	CT2	-70.49(17)
C2B1	C2G1	C2D1	C2E	-59.54(14)	C3	NT	CT1	CT3	52.15(17)
C2B1	C2G2	C2D2	C2G4	-59.80(15)	C3	NT	CT1	CT4	170.11(13)
C2B1	C2G2	C2D2	C2E	60.40(14)	O3	C3	NT	CT1	-4.1(2)
C2B2	C2A	C2B1	C2G1	59.01(13)					

Peptide 2 *bis*-DMSO solvate (LUB125)

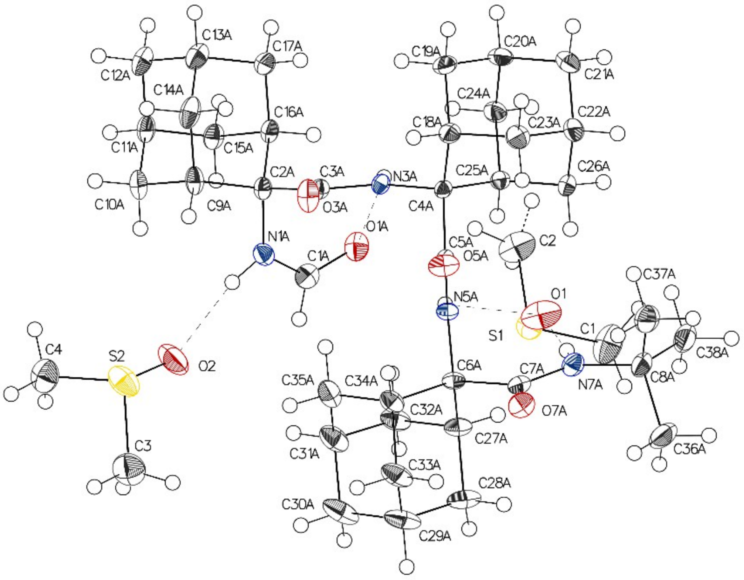

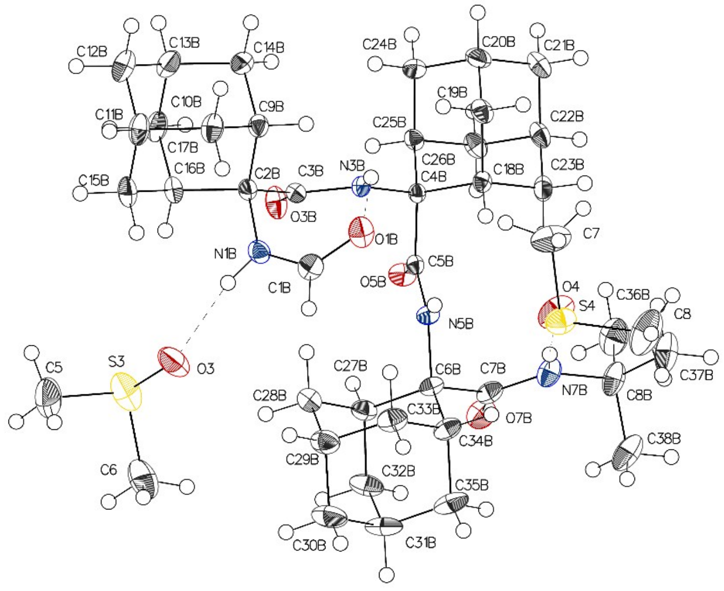

Table 13. Crystal data and structure refinement for LUB125.

Identification code	LUB125
Empirical formula	$C_{42}H_{68}N_4O_6S_2$
Formula weight	789.12
Temperature/K	150
Crystal system	Orthorhombic
Space group	Pbcn
a/Å	9.8603(3)
b/Å	43.0350(14)
c/Å	38.9591(12)
α/°	90
β/°	90
γ/°	90

Volume/Å^3	16531.8(9)
Z	16
ρ_{calc}g/cm^3	1.268
μ/mm^{-1}	1.022
F(000)	6848.0
Crystal size/mm^3	0.38 × 0.13 × 0.06
Radiation	GaKα (λ = 1.34139)
2Θ range for data collection/°	3.572 to 121.596
Index ranges	-12 ≤ h ≤ 12, -55 ≤ k ≤ 55, -50 ≤ l ≤ 50
Reflections collected	305415
Independent reflections	19053 [R_{int} = 0.0575, R_{sigma} = 0.0229]
Data/restraints/parameters	19053/0/993
Goodness-of-fit on F^2	1.110
Final R indexes [I>=2σ (I)]	R_1 = 0.0625, wR_2 = 0.1585
Final R indexes [all data]	R_1 = 0.0715, wR_2 = 0.1645
Largest diff. peak/hole / e Å^{-3}	0.81/-0.79
CCDC deposition No.	1906517

Table 14. Hydrogen Bonds for LUB125.

D	H	A	d(D-H)/Å	d(H-A)/Å	d(D-A)/Å	D-H-A/°
N1B	H1B	O3	0.88	1.93	2.787(2)	165.8
N3B	H3B	O1B	0.88	2.12	2.797(2)	133.4
N5B	H5B	O4	0.88	2.64	3.171(2)	119.5
N7B	H7B	O4	0.88	2.19	3.072(2)	176.6
N1A	H1A	O2	0.88	1.96	2.812(2)	162.4
N3A	H3A	O1A	0.88	2.22	2.846(2)	127.5
N5A	H5A	O1	0.88	2.45	3.016(2)	122.5
N7A	H7A	O1	0.88	2.30	3.183(2)	175.5

Table 15. Torsion Angles for LUB125.

A	B	C	D	Angle/˚	A	B	C	D	Angle/˚
N1B	C2B	C3B	O3B	108.3(2)	N1A	C2A	C3A	O3A	-103.1(2)
N1B	C2B	C3B	N3B	-69.87(19)	N1A	C2A	C3A	N3A	74.81(18)
N1B	C2B	C9B	C10B	-57.2(2)	N1A	C2A	C9A	C10A	-60.5(2)
N1B	C2B	C9B	C14B	-176.91(15)	N1A	C2A	C9A	C14A	-179.54(15)
N1B	C2B	C16B	C15B	60.6(2)	N1A	C2A	C16A	C15A	57.22(19)
N1B	C2B	C16B	C17B	179.88(16)	N1A	C2A	C16A	C17A	177.12(14)

N3B	C4B	C5B	O5B	112.48(19)	N3A	C4A	C5A	O5A	-114.77(19)
N3B	C4B	C5B	N5B	-66.89(19)	N3A	C4A	C5A	N5A	64.06(19)
N3B	C4B	C18B	C19B	-60.43(18)	N3A	C4A	C18A	C19A	-57.2(2)
N3B	C4B	C18B	C23B	179.72(14)	N3A	C4A	C18A	C23A	-175.57(15)
N3B	C4B	C25B	C24B	57.72(19)	N3A	C4A	C25A	C24A	60.45(18)
N3B	C4B	C25B	C26B	176.25(14)	N3A	C4A	C25A	C26A	-179.66(14)
N5B	C6B	C7B	O7B	-122.2(2)	N5A	C6A	C7A	O7A	125.23(19)
N5B	C6B	C7B	N7B	58.5(2)	N5A	C6A	C7A	N7A	-55.5(2)
N5B	C6B	C27B	C28B	-57.8(2)	N5A	C6A	C27A	C28A	-179.72(17)
N5B	C6B	C27B	C32B	-176.74(16)	N5A	C6A	C27A	C32A	-59.8(2)
N5B	C6B	C34B	C33B	60.1(2)	N5A	C6A	C34A	C33A	176.57(15)
N5B	C6B	C34B	C35B	179.77(17)	N5A	C6A	C34A	C35A	58.0(2)
C1B	N1B	C2B	C3B	74.6(2)	C1A	N1A	C2A	C3A	-75.4(2)
C1B	N1B	C2B	C9B	-48.8(3)	C1A	N1A	C2A	C9A	166.47(18)
C1B	N1B	C2B	C16B	-166.51(19)	C1A	N1A	C2A	C16A	48.6(2)
C2B	N1B	C1B	O1B	-3.1(3)	C2A	N1A	C1A	O1A	4.0(3)
C2B	C9B	C10B	C11B	-61.2(2)	C2A	C9A	C10A	C11A	-60.7(2)
C2B	C9B	C14B	C13B	61.8(2)	C2A	C9A	C14A	C13A	60.7(2)
C2B	C16B	C17B	C13B	-60.0(2)	C2A	C16A	C17A	C13A	-61.43(19)
C3B	N3B	C4B	C5B	-60.0(2)	C3A	N3A	C4A	C5A	59.8(2)
C3B	N3B	C4B	C18B	-179.87(16)	C3A	N3A	C4A	C18A	-62.5(2)
C3B	N3B	C4B	C25B	62.1(2)	C3A	N3A	C4A	C25A	179.93(16)
C3B	C2B	C9B	C10B	-176.44(15)	C3A	C2A	C9A	C10A	-176.02(16)
C3B	C2B	C9B	C14B	63.80(19)	C3A	C2A	C9A	C14A	65.0(2)
C3B	C2B	C16B	C15B	175.74(16)	C3A	C2A	C16A	C15A	177.27(15)
C3B	C2B	C16B	C17B	-65.0(2)	C3A	C2A	C16A	C17A	-62.83(19)
C4B	N3B	C3B	O3B	-3.2(3)	C4A	N3A	C3A	O3A	3.3(3)
C4B	N3B	C3B	C2B	175.00(15)	C4A	N3A	C3A	C2A	-174.54(15)
C4B	C18B	C19B	C20B	-60.8(2)	C4A	C18A	C19A	C20A	-60.5(2)
C4B	C18B	C23B	C22B	61.76(19)	C4A	C18A	C23A	C22A	59.3(2)
C4B	C25B	C26B	C22B	-59.8(2)	C4A	C25A	C26A	C22A	-62.1(2)
C5B	N5B	C6B	C7B	61.1(2)	C5A	N5A	C6A	C7A	-59.0(2)
C5B	N5B	C6B	C27B	-62.2(2)	C5A	N5A	C6A	C27A	-177.35(16)
C5B	N5B	C6B	C34B	179.80(16)	C5A	N5A	C6A	C34A	64.5(2)
C5B	C4B	C18B	C19B	-178.61(14)	C5A	C4A	C18A	C19A	-177.89(15)
C5B	C4B	C18B	C23B	61.54(19)	C5A	C4A	C18A	C23A	63.7(2)
C5B	C4B	C25B	C24B	178.67(14)	C5A	C4A	C25A	C24A	178.07(15)
C5B	C4B	C25B	C26B	-62.80(18)	C5A	C4A	C25A	C26A	-62.04(18)
C6B	N5B	C5B	O5B	3.8(3)	C6A	N5A	C5A	O5A	-5.0(3)
C6B	N5B	C5B	C4B	-176.88(15)	C6A	N5A	C5A	C4A	176.22(15)
C6B	C27B	C28B	C29B	-61.3(2)	C6A	C27A	C28A	C29A	62.2(2)
C6B	C27B	C32B	C31B	58.7(2)	C6A	C27A	C32A	C31A	-60.0(2)
C6B	C34B	C35B	C31B	-62.2(2)	C6A	C34A	C35A	C31A	61.3(2)
C7B	N7B	C8B	C36B	63.6(3)	C7A	N7A	C8A	C36A	61.0(3)
C7B	N7B	C8B	C37B	-177.1(2)	C7A	N7A	C8A	C37A	-61.2(2)

C7B	N7B	C8B	C38B	-58.9(3)	C7A	N7A	C8A	C38A	179.81(18)
C7B	C6B	C27B	C28B	-178.05(16)	C7A	C6A	C27A	C28A	63.7(2)
C7B	C6B	C27B	C32B	63.0(2)	C7A	C6A	C27A	C32A	-176.44(15)
C7B	C6B	C34B	C33B	176.99(17)	C7A	C6A	C34A	C33A	-63.2(2)
C7B	C6B	C34B	C35B	-63.4(2)	C7A	C6A	C34A	C35A	178.22(16)
C8B	N7B	C7B	O7B	-5.4(3)	C8A	N7A	C7A	O7A	0.7(3)
C8B	N7B	C7B	C6B	173.91(19)	C8A	N7A	C7A	C6A	-178.57(17)
C9B	C2B	C3B	O3B	-128.8(2)	C9A	C2A	C3A	O3A	13.2(3)
C9B	C2B	C3B	N3B	53.0(2)	C9A	C2A	C3A	N3A	-168.94(16)
C9B	C2B	C16B	C15B	-60.0(2)	C9A	C2A	C16A	C15A	-60.59(19)
C9B	C2B	C16B	C17B	59.3(2)	C9A	C2A	C16A	C17A	59.32(18)
C9B	C10B	C11B	C12B	-60.2(2)	C9A	C10A	C11A	C12A	-60.3(2)
C9B	C10B	C11B	C15B	59.8(2)	C9A	C10A	C11A	C15A	59.7(2)
C10B	C9B	C14B	C13B	-58.4(2)	C10A	C9A	C14A	C13A	-59.4(2)
C10B	C11B	C12B	C13B	60.3(2)	C10A	C11A	C12A	C13A	58.8(2)
C10B	C11B	C15B	C16B	-59.3(2)	C10A	C11A	C15A	C16A	-60.7(2)
C11B	C12B	C13B	C14B	-59.8(2)	C11A	C12A	C13A	C14A	-58.3(2)
C11B	C12B	C13B	C17B	59.0(2)	C11A	C12A	C13A	C17A	60.3(2)
C11B	C15B	C16B	C2B	60.7(2)	C11A	C15A	C16A	C2A	62.1(2)
C11B	C15B	C16B	C17B	-60.4(2)	C11A	C15A	C16A	C17A	-58.1(2)
C12B	C11B	C15B	C16B	60.4(2)	C12A	C11A	C15A	C16A	59.4(2)
C12B	C13B	C14B	C9B	59.3(2)	C12A	C13A	C14A	C9A	59.7(2)
C12B	C13B	C17B	C16B	-60.6(2)	C12A	C13A	C17A	C16A	-59.8(2)
C14B	C9B	C10B	C11B	58.7(2)	C14A	C9A	C10A	C11A	59.6(2)
C14B	C13B	C17B	C16B	58.9(2)	C14A	C13A	C17A	C16A	60.2(2)
C15B	C11B	C12B	C13B	-58.9(2)	C15A	C11A	C12A	C13A	-60.3(2)
C15B	C16B	C17B	C13B	60.5(2)	C15A	C16A	C17A	C13A	58.4(2)
C16B	C2B	C3B	O3B	-7.6(3)	C16A	C2A	C3A	O3A	133.76(19)
C16B	C2B	C3B	N3B	174.19(16)	C16A	C2A	C3A	N3A	-48.4(2)
C16B	C2B	C9B	C10B	60.2(2)	C16A	C2A	C9A	C10A	60.1(2)
C16B	C2B	C9B	C14B	-59.52(19)	C16A	C2A	C9A	C14A	-58.94(19)
C17B	C13B	C14B	C9B	-60.4(2)	C17A	C13A	C14A	C9A	-59.8(2)
C18B	C4B	C5B	O5B	-130.31(18)	C18A	C4A	C5A	O5A	8.3(2)
C18B	C4B	C5B	N5B	50.3(2)	C18A	C4A	C5A	N5A	-172.85(15)
C18B	C4B	C25B	C24B	-59.68(18)	C18A	C4A	C25A	C24A	-59.90(18)
C18B	C4B	C25B	C26B	58.86(19)	C18A	C4A	C25A	C26A	59.99(18)
C18B	C19B	C20B	C21B	-60.2(2)	C18A	C19A	C20A	C21A	-59.9(2)
C18B	C19B	C20B	C24B	59.3(2)	C18A	C19A	C20A	C24A	59.5(2)
C19B	C18B	C23B	C22B	-58.5(2)	C19A	C18A	C23A	C22A	-60.2(2)
C19B	C20B	C21B	C22B	59.7(2)	C19A	C20A	C21A	C22A	58.9(2)
C19B	C20B	C24B	C25B	-59.2(2)	C19A	C20A	C24A	C25A	-59.1(2)
C20B	C21B	C22B	C23B	-59.7(2)	C20A	C21A	C22A	C23A	-59.3(2)
C20B	C21B	C22B	C26B	59.7(2)	C20A	C21A	C22A	C26A	59.9(2)
C20B	C24B	C25B	C4B	60.4(2)	C20A	C24A	C25A	C4A	60.4(2)
C20B	C24B	C25B	C26B	-59.6(2)	C20A	C24A	C25A	C26A	-59.8(2)

C21B	C20B	C24B	C25B	60.1(2)	C21A	C20A	C24A	C25A	61.1(2)
C21B	C22B	C23B	C18B	59.2(2)	C21A	C22A	C23A	C18A	61.0(2)
C21B	C22B	C26B	C25B	-60.6(2)	C21A	C22A	C26A	C25A	-59.1(2)
C23B	C18B	C19B	C20B	59.4(2)	C23A	C18A	C19A	C20A	59.5(2)
C23B	C22B	C26B	C25B	59.7(2)	C23A	C22A	C26A	C25A	60.6(2)
C24B	C20B	C21B	C22B	-59.4(2)	C24A	C20A	C21A	C22A	-60.7(2)
C24B	C25B	C26B	C22B	59.7(2)	C24A	C25A	C26A	C22A	58.6(2)
C25B	C4B	C5B	O5B	-10.5(2)	C25A	C4A	C5A	O5A	128.59(18)
C25B	C4B	C5B	N5B	170.13(15)	C25A	C4A	C5A	N5A	-52.6(2)
C25B	C4B	C18B	C19B	60.16(18)	C25A	C4A	C18A	C19A	59.50(18)
C25B	C4B	C18B	C23B	-59.69(19)	C25A	C4A	C18A	C23A	-58.88(19)
C26B	C22B	C23B	C18B	-60.7(2)	C26A	C22A	C23A	C18A	-58.8(2)
C27B	C6B	C7B	O7B	0.1(3)	C27A	C6A	C7A	O7A	-117.5(2)
C27B	C6B	C7B	N7B	-179.27(17)	C27A	C6A	C7A	N7A	61.7(2)
C27B	C6B	C34B	C33B	-59.8(2)	C27A	C6A	C34A	C33A	58.2(2)
C27B	C6B	C34B	C35B	59.8(2)	C27A	C6A	C34A	C35A	-60.4(2)
C27B	C28B	C29B	C30B	-59.4(2)	C27A	C28A	C29A	C30A	58.9(2)
C27B	C28B	C29B	C33B	59.7(2)	C27A	C28A	C29A	C33A	-61.2(2)
C28B	C27B	C32B	C31B	-61.1(2)	C28A	C27A	C32A	C31A	60.0(2)
C28B	C29B	C30B	C31B	58.6(3)	C28A	C29A	C30A	C31A	-60.3(3)
C28B	C29B	C33B	C34B	-58.6(2)	C28A	C29A	C33A	C34A	59.0(2)
C29B	C30B	C31B	C32B	-59.6(3)	C29A	C30A	C31A	C32A	61.0(3)
C29B	C30B	C31B	C35B	60.1(3)	C29A	C30A	C31A	C35A	-58.4(3)
C29B	C33B	C34B	C6B	59.6(2)	C29A	C33A	C34A	C6A	-59.0(2)
C29B	C33B	C34B	C35B	-59.9(2)	C29A	C33A	C34A	C35A	60.2(2)
C30B	C29B	C33B	C34B	61.3(2)	C30A	C29A	C33A	C34A	-61.2(2)
C30B	C31B	C32B	C27B	61.7(3)	C30A	C31A	C32A	C27A	-61.5(2)
C30B	C31B	C35B	C34B	-58.9(2)	C30A	C31A	C35A	C34A	59.2(2)
C32B	C27B	C28B	C29B	59.6(2)	C32A	C27A	C28A	C29A	-58.4(2)
C32B	C31B	C35B	C34B	60.9(2)	C32A	C31A	C35A	C34A	-59.7(2)
C33B	C29B	C30B	C31B	-60.9(3)	C33A	C29A	C30A	C31A	59.3(3)
C33B	C34B	C35B	C31B	58.3(2)	C33A	C34A	C35A	C31A	-59.1(2)
C34B	C6B	C7B	O7B	120.5(2)	C34A	C6A	C7A	O7A	2.6(3)
C34B	C6B	C7B	N7B	-58.8(2)	C34A	C6A	C7A	N7A	-178.08(16)
C34B	C6B	C27B	C28B	60.3(2)	C34A	C6A	C27A	C28A	-59.4(2)
C34B	C6B	C27B	C32B	-58.7(2)	C34A	C6A	C27A	C32A	60.45(19)
C35B	C31B	C32B	C27B	-58.4(3)	C35A	C31A	C32A	C27A	58.7(2)

Table 16. Atomic Occupancy for LUB125.

**Atom**	***Occupancy***	**Atom**	***Occupancy***	**Atom**	***Occupancy***
S1	0.894(2)	S1B	0.106(2)	H2AA	0.894(2)
H2AB	0.894(2)	H2AC	0.894(2)	H2BD	0.106(2)
H2BE	0.106(2)	H2BF	0.106(2)		

NMR

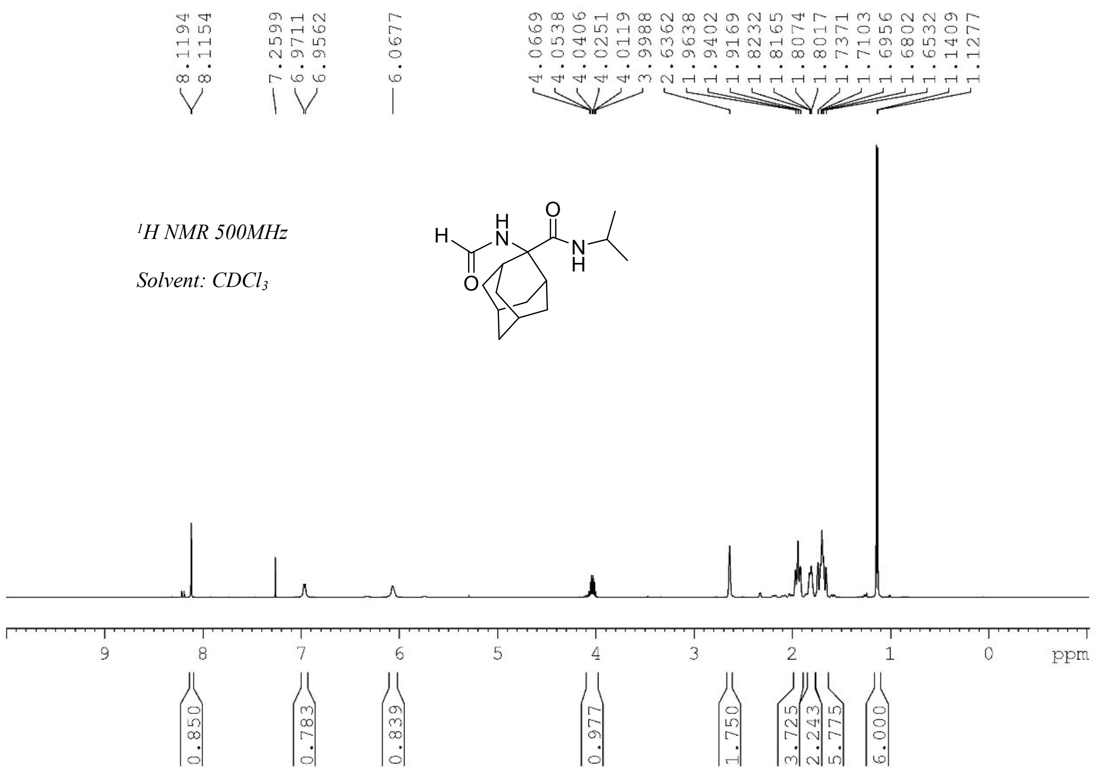
8.1194
8.1154
7.2599
6.9711
6.9562
6.0677
4.0669
4.0538
4.0406
4.0251
4.0119
3.9988
2.6362
1.9638
1.9402
1.9169
1.8232
1.8165
1.8074
1.8017
1.7371
1.7103
1.6956
1.6802
1.6532
1.1409
1.1277
1H NMR 500MHz
Solvent: CDCl3
9
8
7
6
5
4
3
2
1
0
ppm
0.850
0.783
0.839
0.977
1.750
3.725
2.243
5.775
6.000

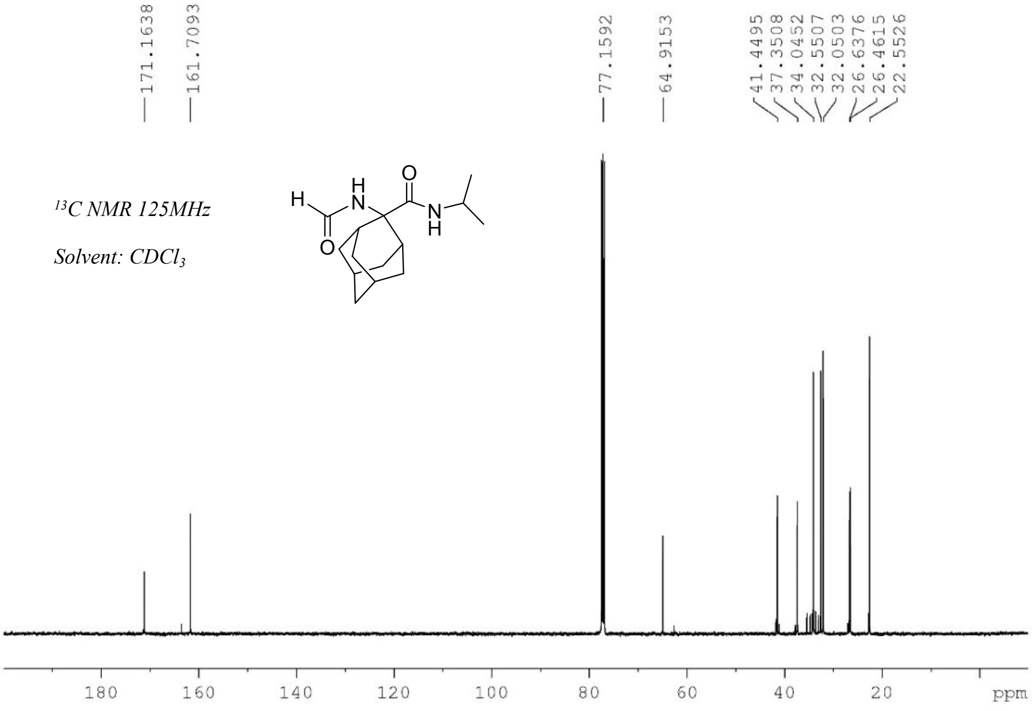
171.1638
161.7093
77.1592
64.9153
41.4495
37.3508
34.0452
32.5507
32.0503
26.6376
26.4615
22.5526
13C NMR 125MHz
Solvent: CDCl3
180
160
140
120
100
80
60
40
20
ppm

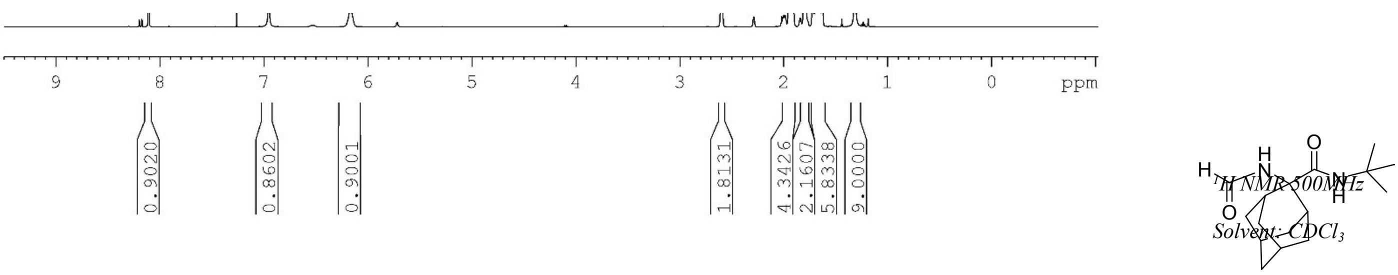
9
8
7
6
5
4
3
2
1
0
ppm
0.9020
0.8602
0.9001
1.8131
4.3426
2.1607
5.8338
9.0000
Solvent: CDCl3

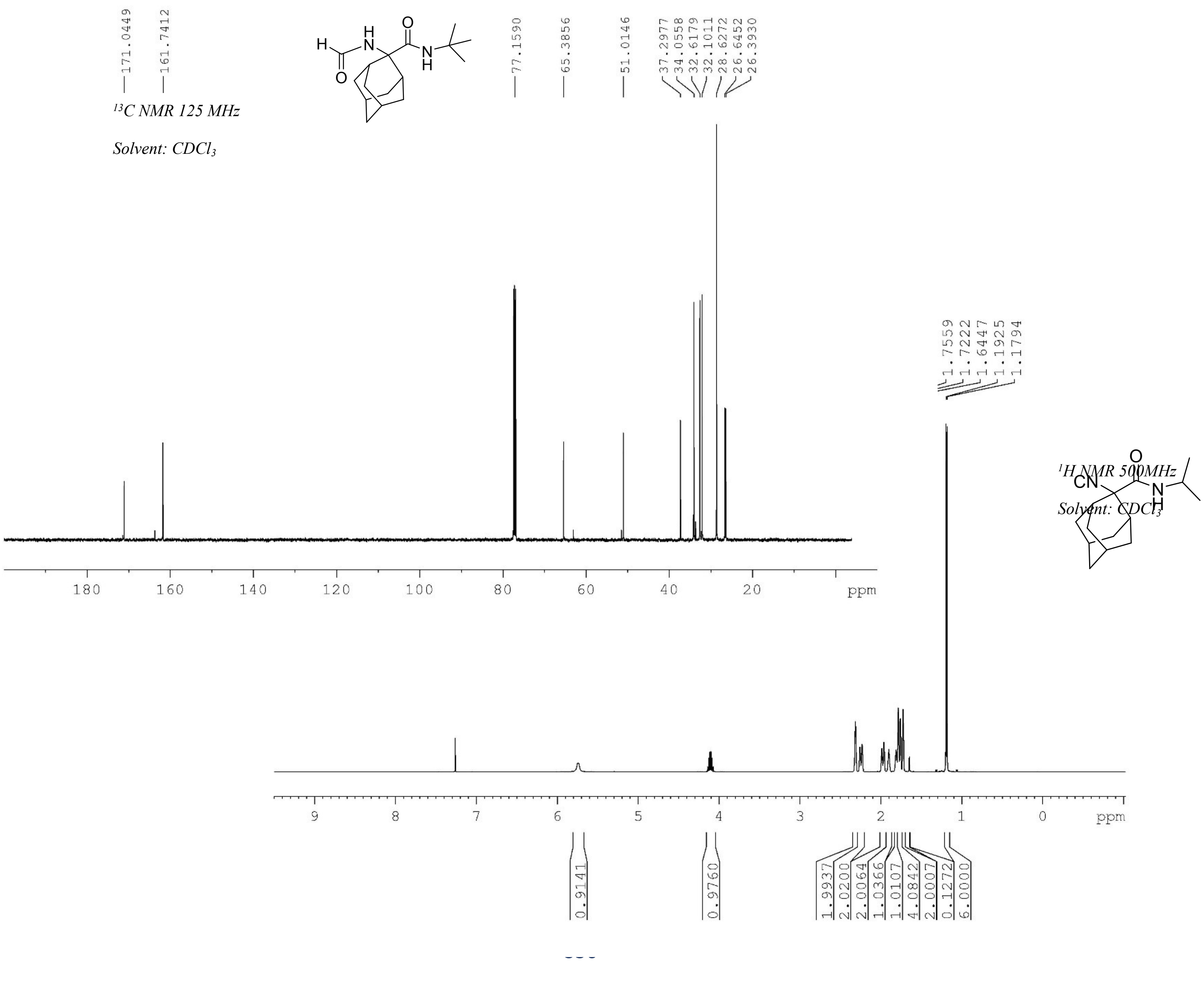

171.0449
161.7412
^{13}C NMR 125 MHz
Solvent: $CDCl_3$
77.1590
65.3856
51.0146
37.2977
34.0558
32.6179
32.1011
28.6272
26.6452
26.3930
180 160 140 120 100 80 60 40 20 ppm
1.7559
1.7222
1.6447
1.1925
1.1794
^{1}H NMR 500MHz
Solvent: $CDCl_3$
CN
9 8 7 6 5 4 3 2 1 0 ppm
0.9141
0.9760
1.9937
2.0200
2.0064
1.0366
1.0107
4.0842
2.0007
0.1272
6.0000

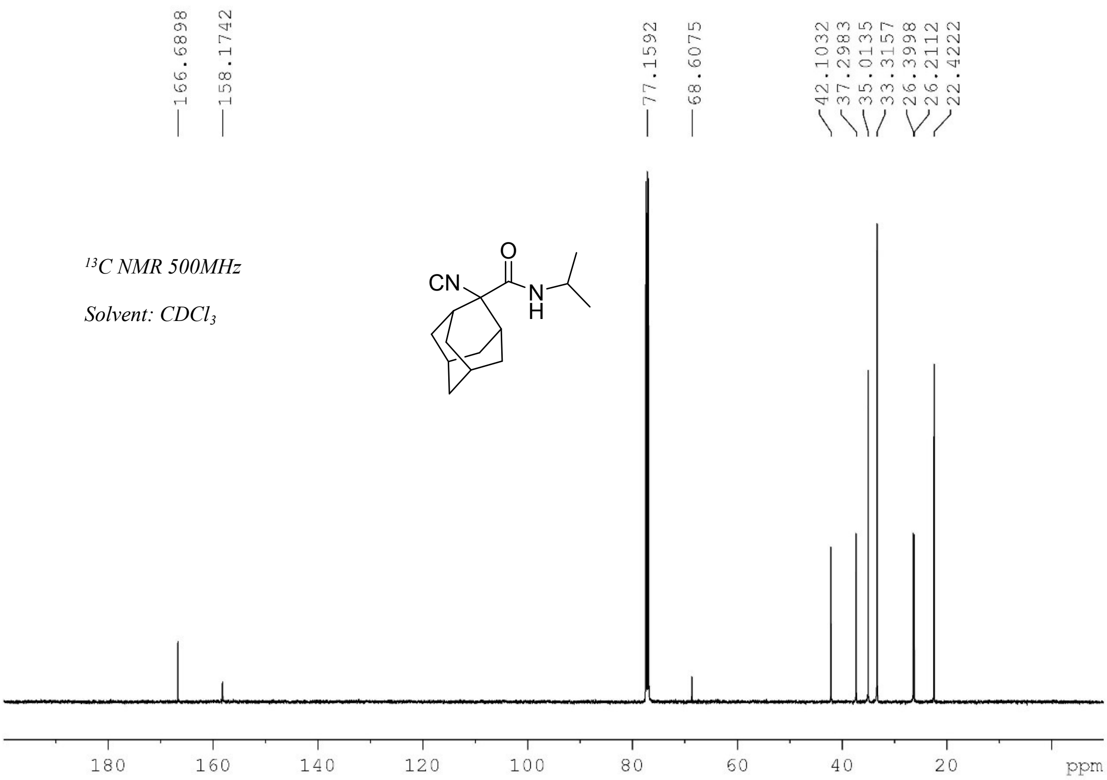
166.6898
158.1742
77.1592
68.6075
42.1032
37.2983
35.0135
33.3157
26.3998
26.2112
22.4222
13C NMR 500MHz
Solvent: CDCl3
CN
O
N
H
180
160
140
120
100
80
60
40
20
ppm

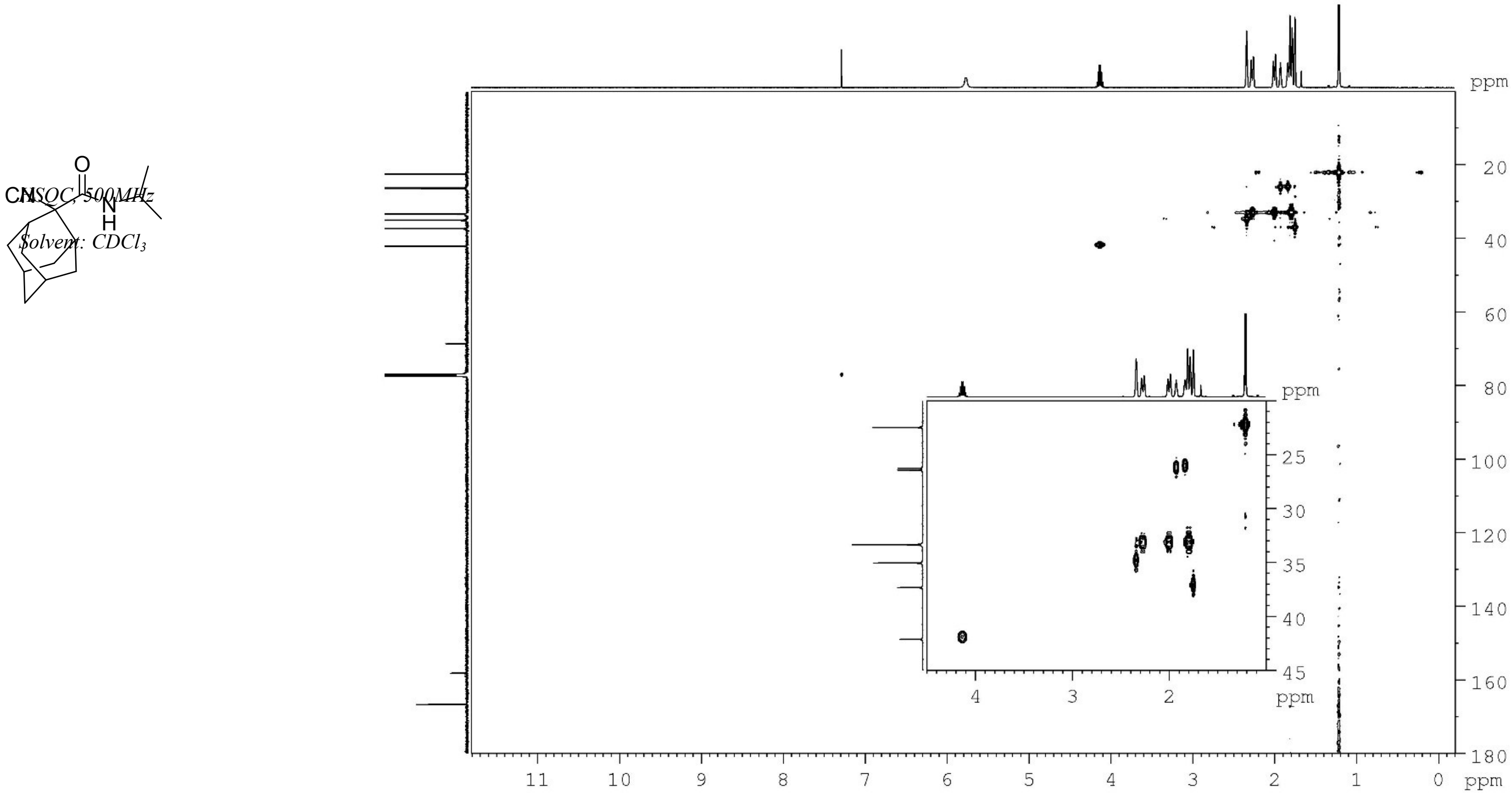
HSQC, 500MHz
Solvent: CDCl3
CN
O
N
H
ppm
20
40
60
80
100
120
140
160
180
11
10
9
8
7
6
5
4
3
2
1
0
ppm
25
30
35
40
45
4
3
2
ppm

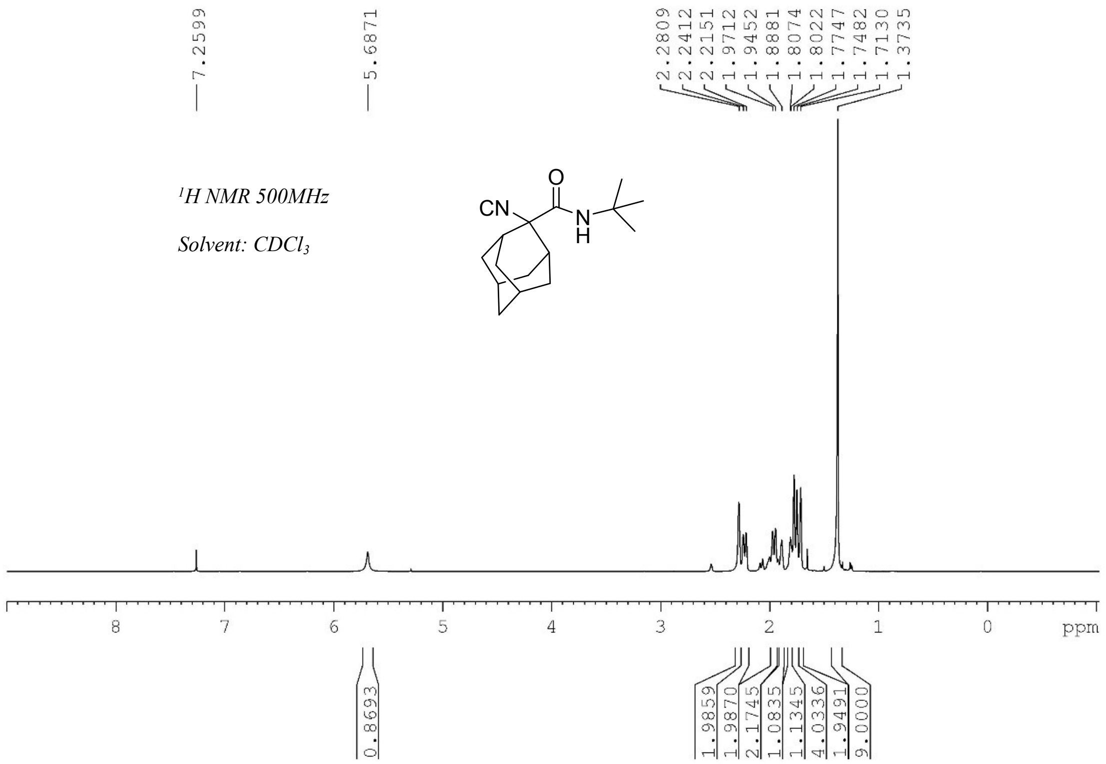
7.2599
5.6871
2.2809
2.2412
2.2151
1.9712
1.9452
1.8881
1.8074
1.8022
1.7747
1.7482
1.7130
1.3735
1H NMR 500MHz
Solvent: CDCl3
CN
O
N
H
8
7
6
5
4
3
2
1
0
ppm
0.8693
1.9859
1.9870
2.1745
1.0835
1.1345
4.0336
1.9491
9.0000

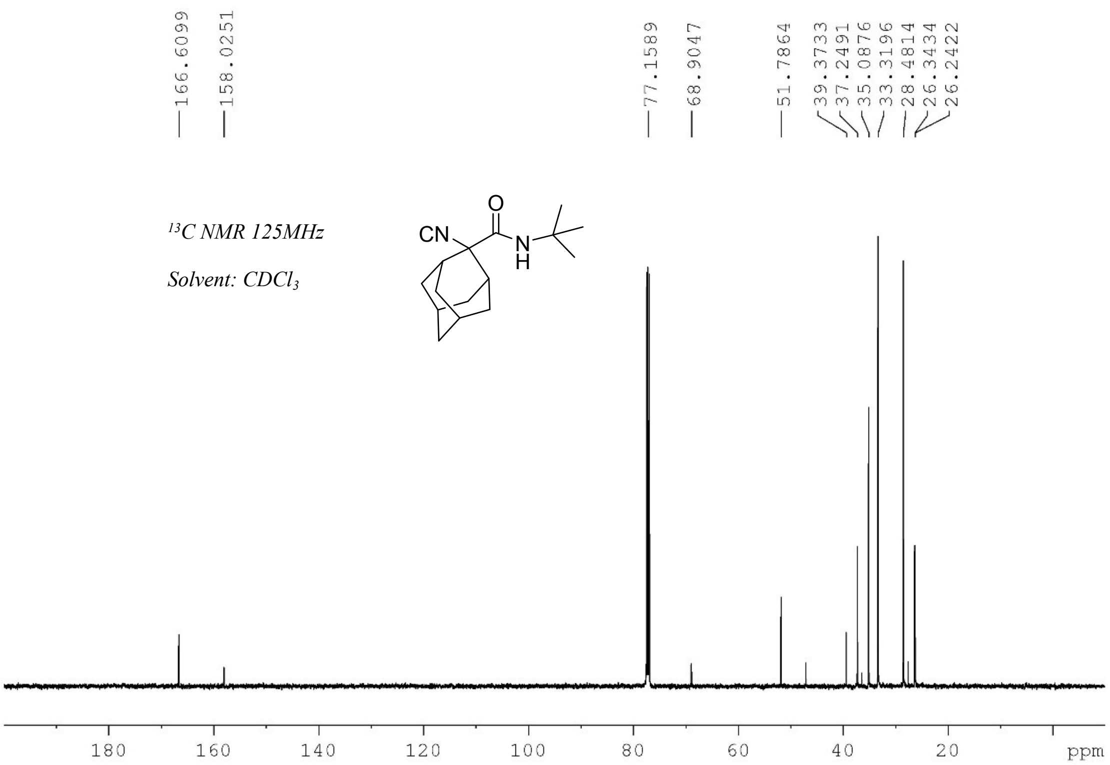
166.6099
158.0251
77.1589
68.9047
51.7864
39.3733
37.2491
35.0876
33.3196
28.4814
26.3434
26.2422
13C NMR 125MHz
Solvent: CDCl3
CN
O
N
H
180
160
140
120
100
80
60
40
20
ppm

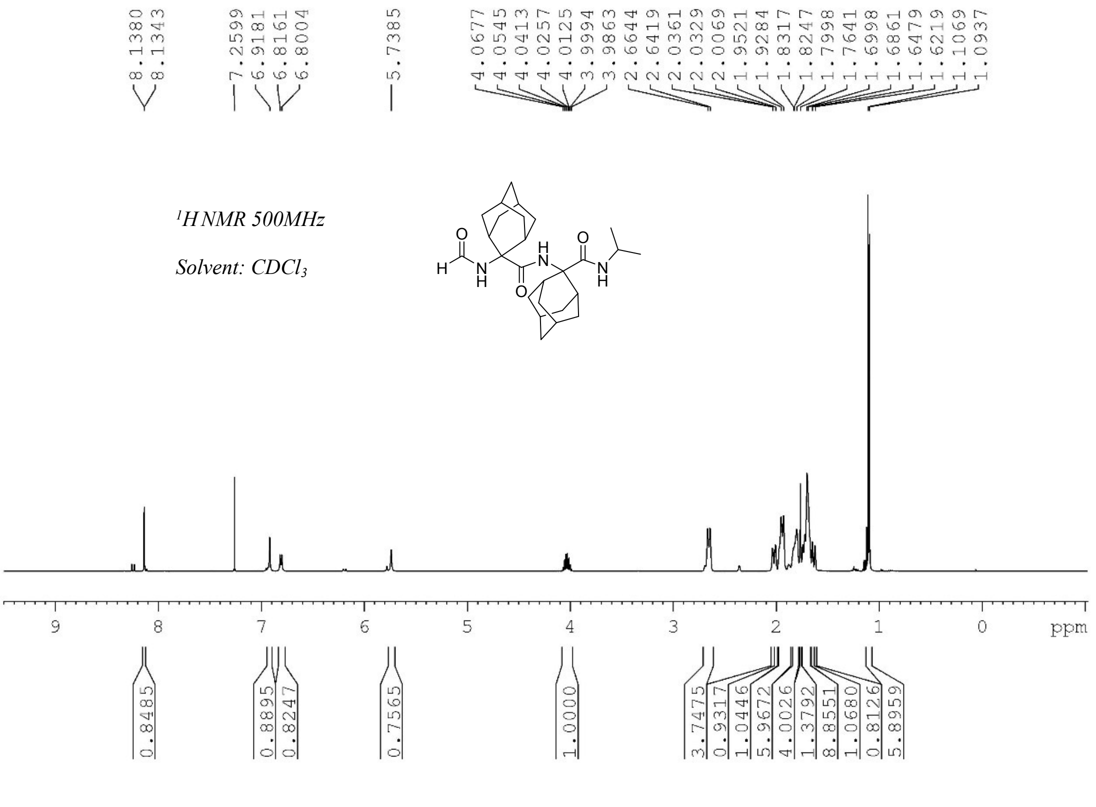
8.1380
8.1343
7.2599
6.9181
6.8161
6.8004
5.7385
4.0677
4.0545
4.0413
4.0257
4.0125
3.9994
3.9863
2.6644
2.6419
2.0361
2.0329
2.0069
1.9521
1.9284
1.8317
1.8247
1.7998
1.7641
1.6998
1.6861
1.6479
1.6219
1.1069
1.0937
1H NMR 500MHz
Solvent: CDCl3
O
H
N
H
O
H
N
O
N
H
9
8
7
6
5
4
3
2
1
0
ppm
0.8485
0.8895
0.8247
0.7565
1.0000
3.7475
0.9317
1.0446
5.9672
4.0026
1.3792
8.8551
1.0680
0.8126
5.8959

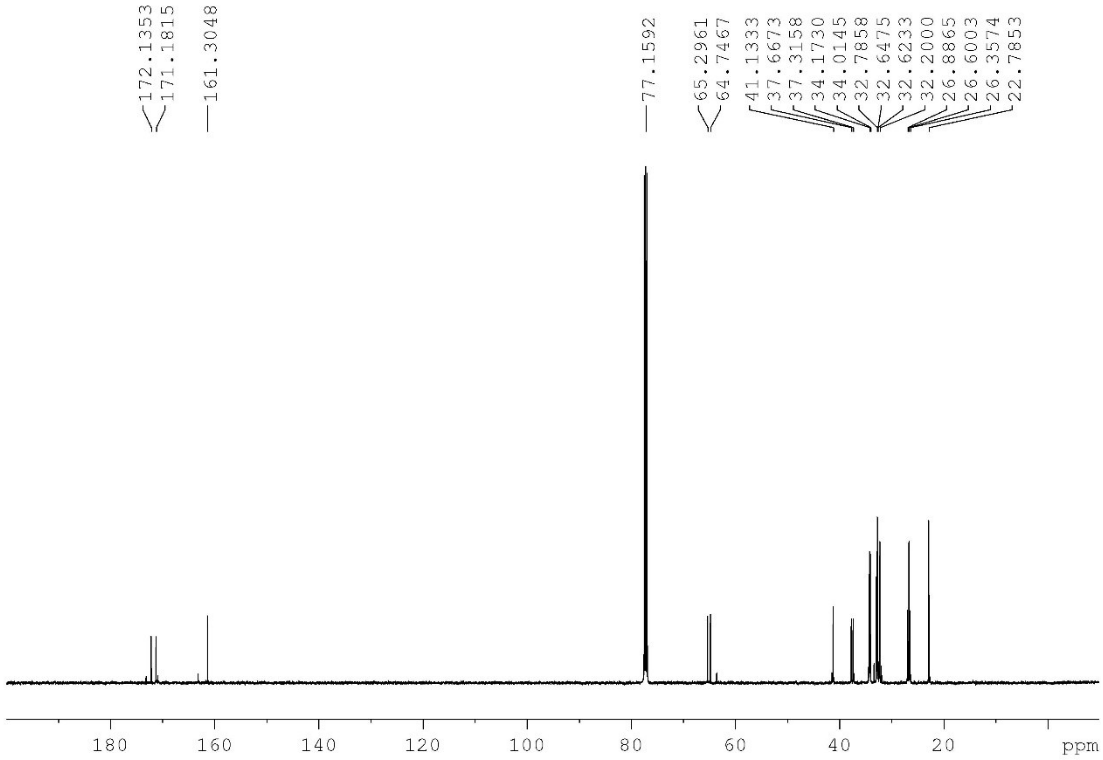
172.1353
171.1815
161.3048
77.1592
65.2961
64.7467
41.1333
37.6673
37.3158
34.1730
34.0145
32.7858
32.6475
32.6233
32.2000
26.8865
26.6003
26.3574
22.7853
180 160 140 120 100 80 60 40 20 ppm

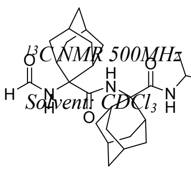
13C NMR 500MHz
Solvent: CDCl3

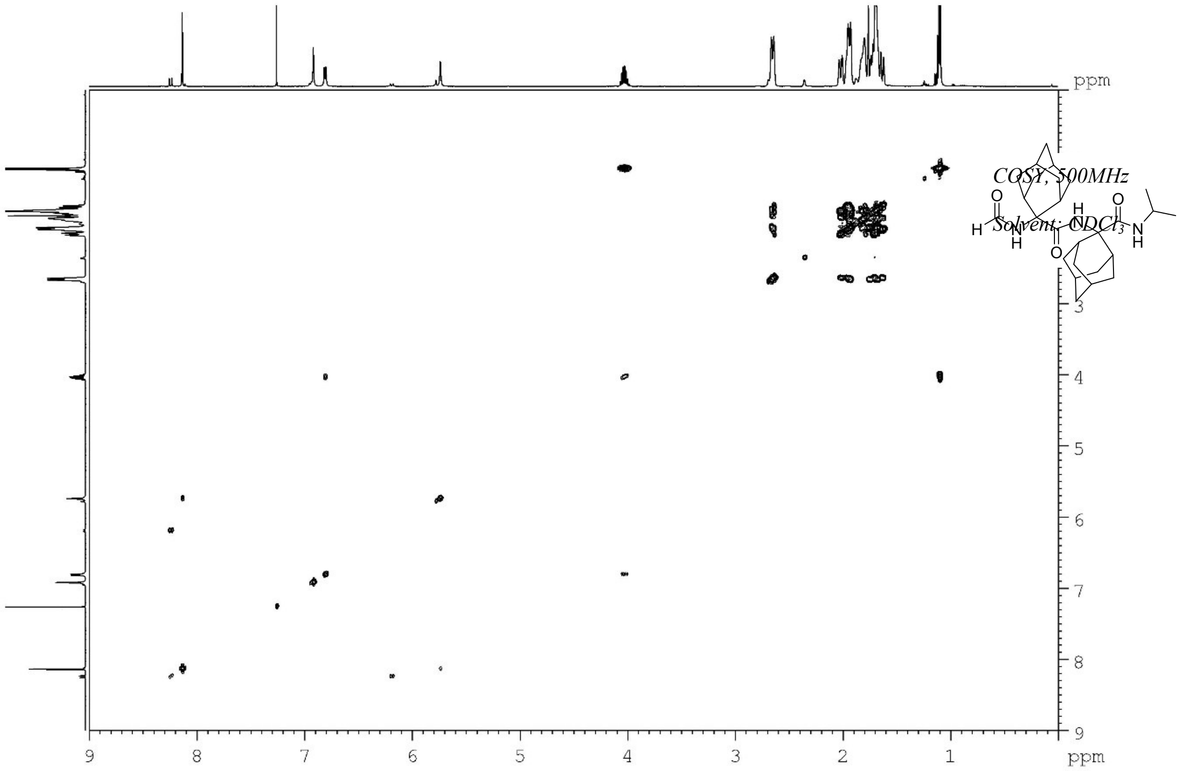
COSY, 500MHz
Solvent: CDCl3
9 8 7 6 5 4 3 2 1 ppm

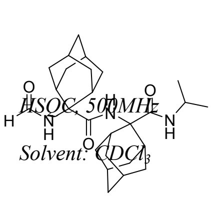
HSQC, 500MHz
Solvent: CDCl3

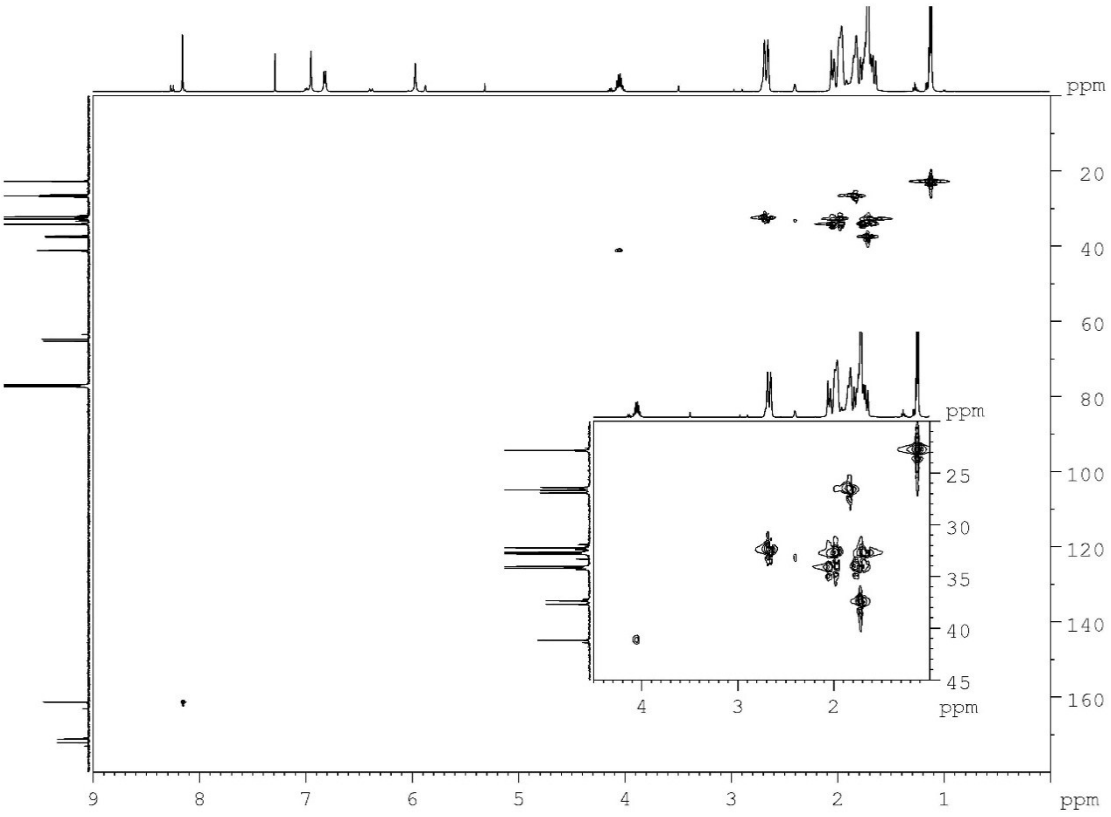

ppm
20
40
60
80
100
120
140
160
9
8
7
6
5
4
3
2
1
ppm
25
30
35
40
45
4
3
2
ppm
ppm

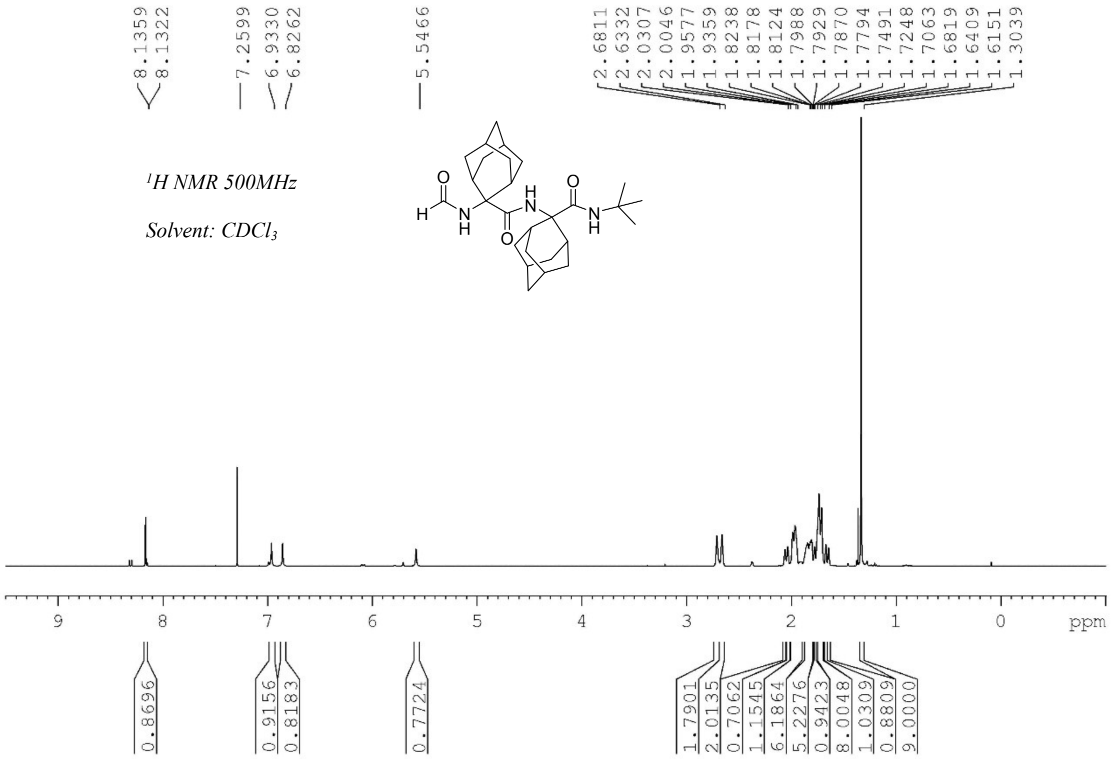
8.1359
8.1322
7.2599
6.9330
6.8262
5.5466
2.6811
2.6332
2.0307
2.0046
1.9577
1.9359
1.8238
1.8178
1.8124
1.7988
1.7929
1.7870
1.7794
1.7491
1.7248
1.7063
1.6819
1.6409
1.6151
1.3039
^{1}H NMR 500MHz
Solvent: $CDCl_3$
9
8
7
6
5
4
3
2
1
0
ppm
0.8696
0.9156
0.8183
0.7724
1.7901
2.0135
0.7062
1.1545
6.1864
5.2276
0.9423
8.0048
1.0309
0.8809
9.0000

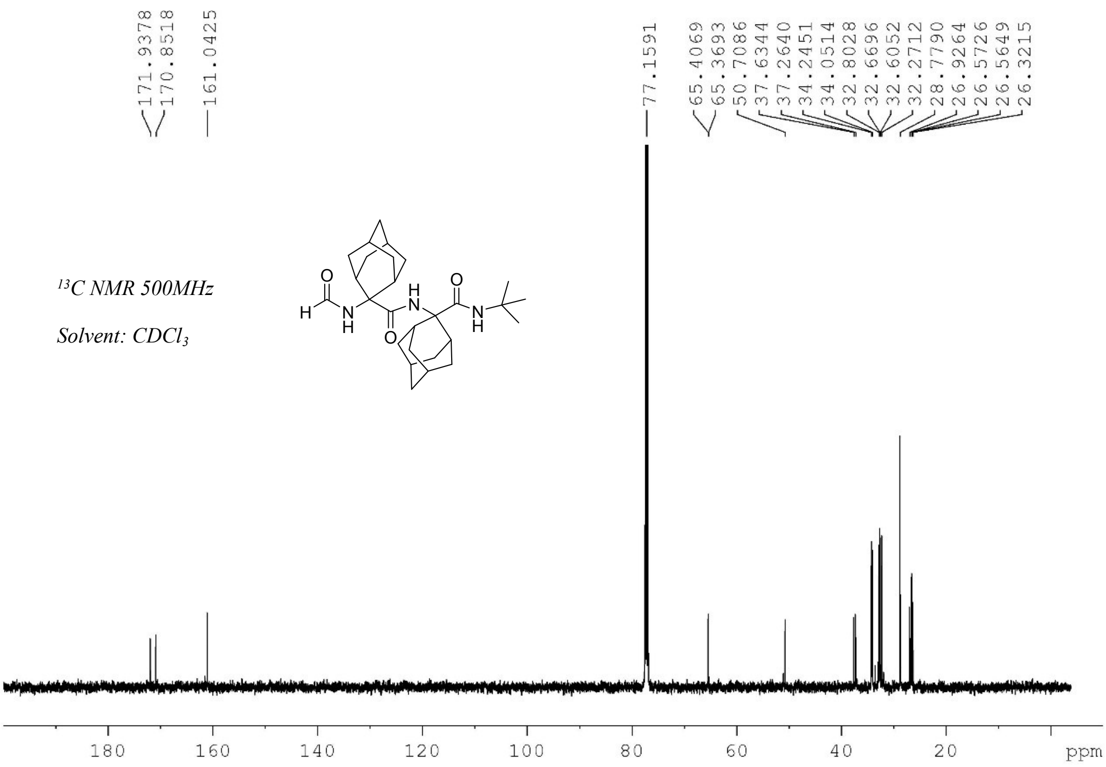
171.9378
170.8518
161.0425
77.1591
65.4069
65.3693
50.7086
37.6344
37.2640
34.2451
34.0514
32.8028
32.6696
32.6052
32.2712
28.7790
26.9264
26.5726
26.5649
26.3215
^{13}C NMR 500MHz
Solvent: $CDCl_3$
180
160
140
120
100
80
60
40
20
ppm

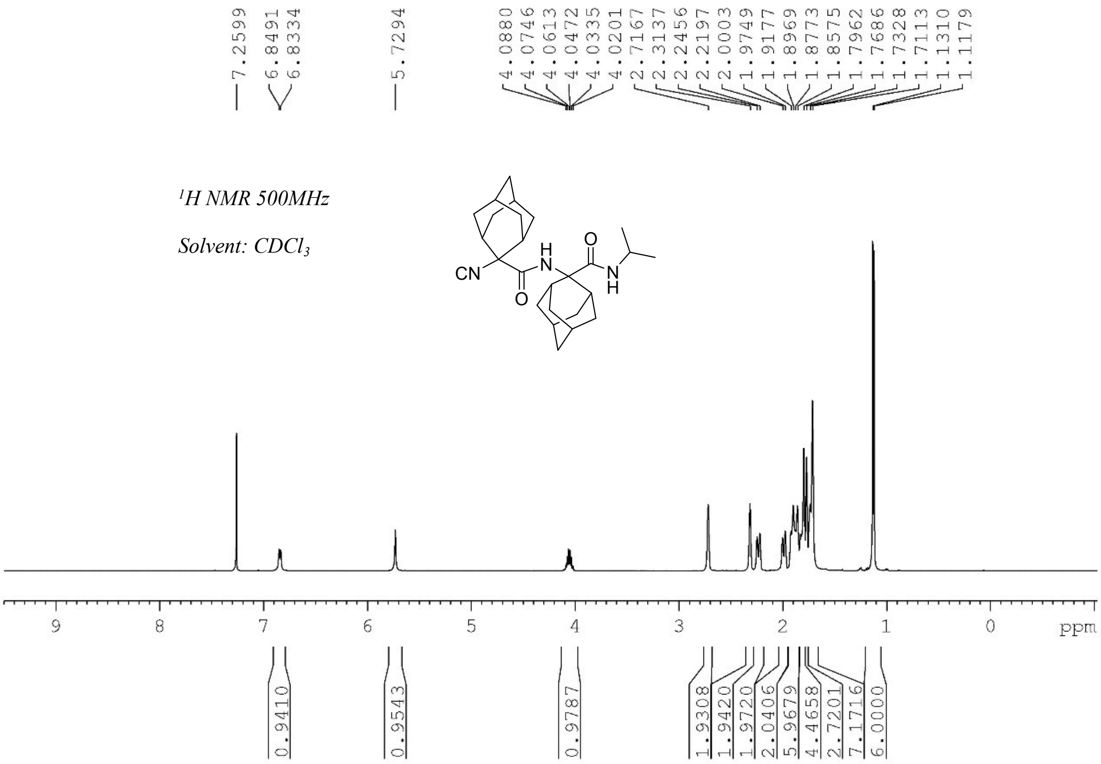

^{1}H NMR 500MHz
Solvent: $CDCl_3$
CN
NH
O
N
H
7.2599
6.8491
6.8334
5.7294
4.0880
4.0746
4.0613
4.0472
4.0335
4.0201
2.7167
2.3137
2.2456
2.2197
2.0003
1.9749
1.9177
1.8969
1.8773
1.8575
1.7962
1.7686
1.7328
1.7113
1.1310
1.1179
9
8
7
6
5
4
3
2
1
0
ppm
0.9410
0.9543
0.9787
1.9308
1.9420
1.9720
2.0406
5.9679
4.4658
2.7201
7.1716
6.0000

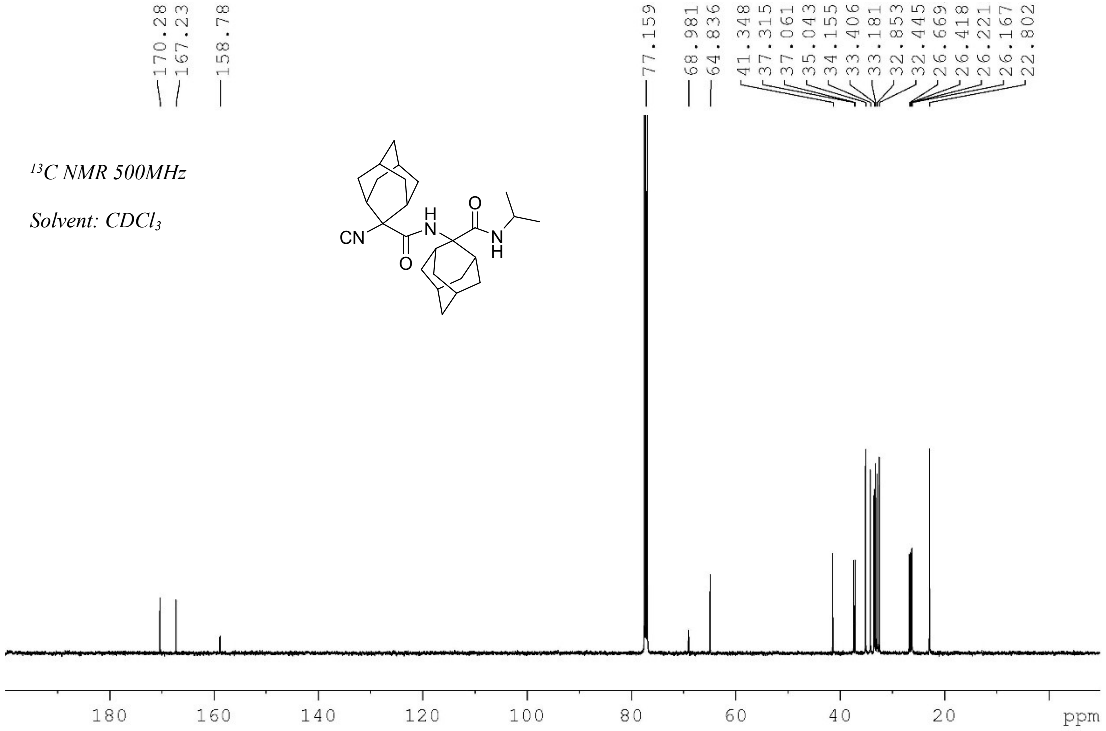

^{13}C NMR 500MHz
Solvent: $CDCl_3$
CN
NH
O
N
H
170.28
167.23
158.78
77.159
68.981
64.836
41.348
37.315
37.061
35.043
34.155
33.406
33.181
32.853
32.445
26.669
26.418
26.221
26.167
22.802
180
160
140
120
100
80
60
40
20
ppm

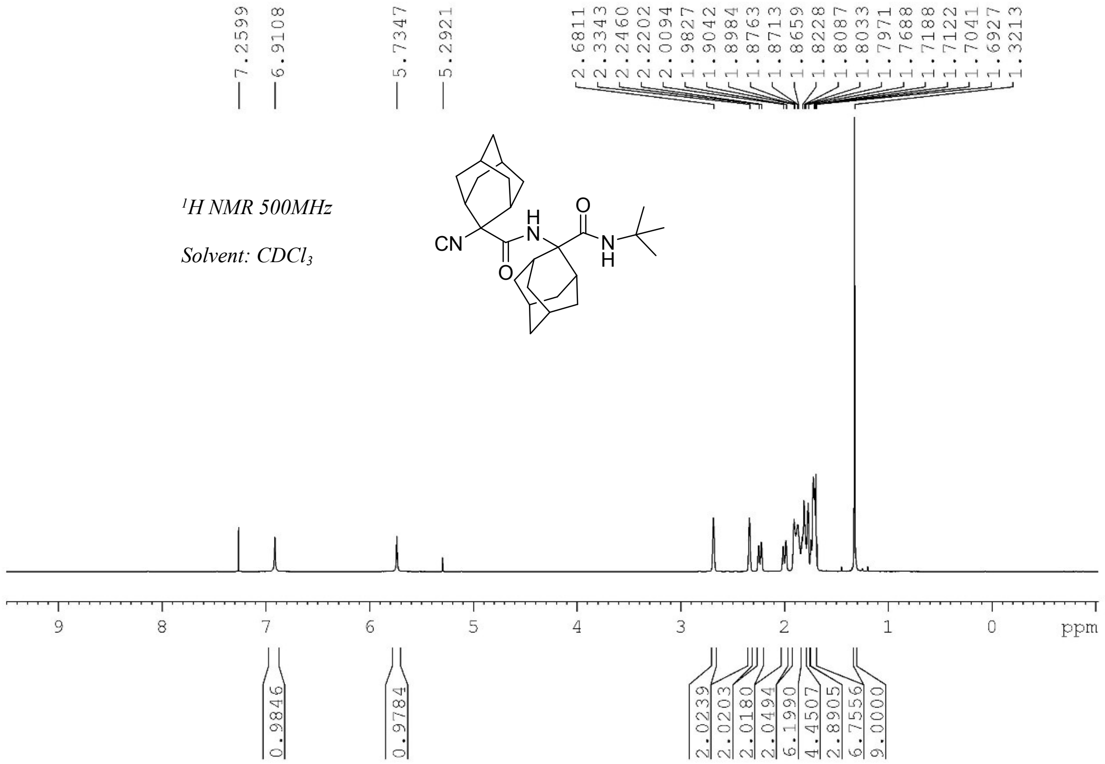

7.2599
6.9108
5.7347
5.2921
2.6811
2.3343
2.2460
2.2202
2.0094
1.9827
1.9042
1.8984
1.8763
1.8713
1.8659
1.8228
1.8087
1.8033
1.7971
1.7688
1.7188
1.7122
1.7041
1.6927
1.3213
1H NMR 500MHz
Solvent: CDCl3
CN
H
N
O
O
N
H
9
8
7
6
5
4
3
2
1
0
ppm
0.9846
0.9784
2.0239
2.0203
2.0180
2.0494
6.1990
4.4507
2.8905
6.7556
9.0000

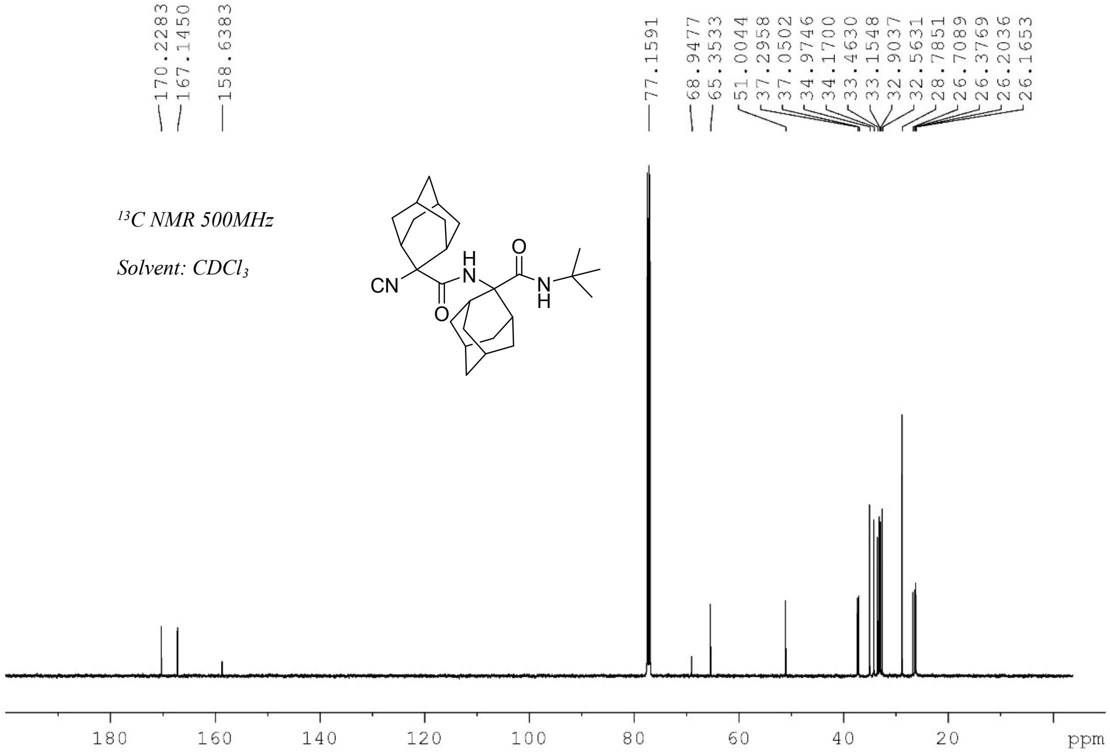

170.2283
167.1450
158.6383
77.1591
68.9477
65.3533
51.0044
37.2958
37.0502
34.9746
34.1700
33.4630
33.1548
32.9037
32.5631
28.7851
26.7089
26.3769
26.2036
26.1653
13C NMR 500MHz
Solvent: CDCl3
CN
H
N
O
O
N
H
180
160
140
120
100
80
60
40
20
ppm

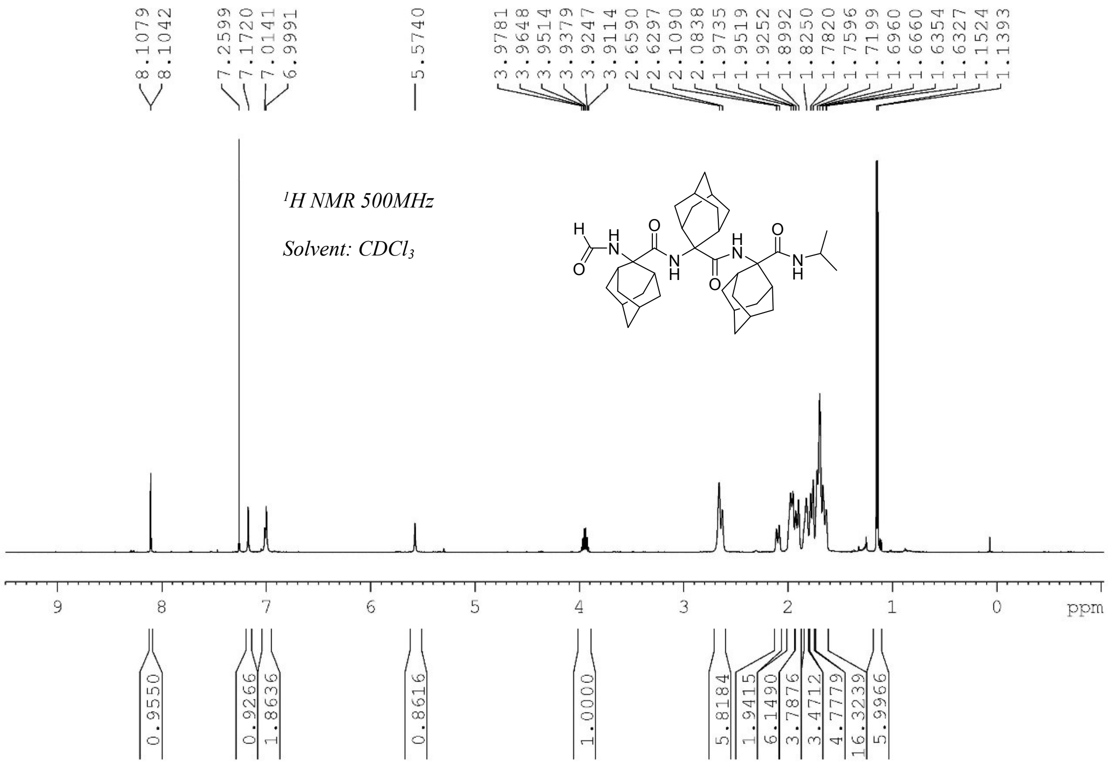

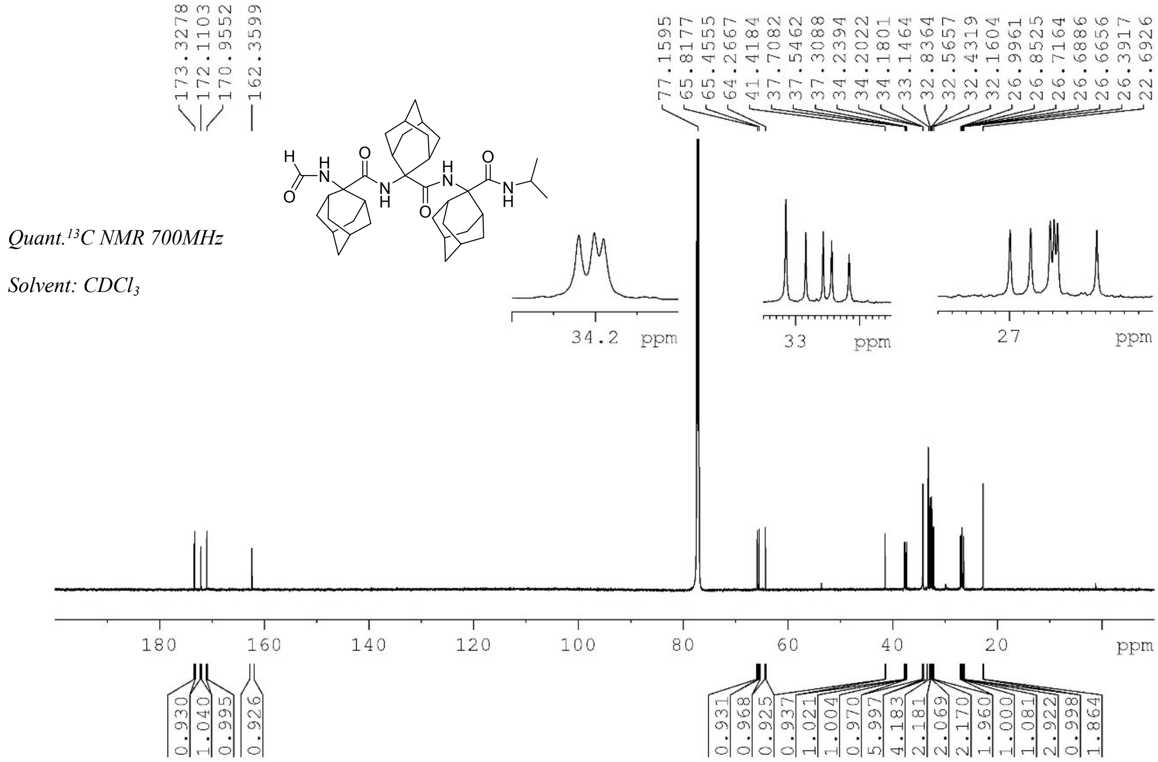

COSY 500MHz

Solvent: $CDCl_3$

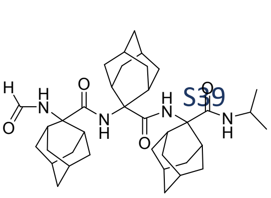

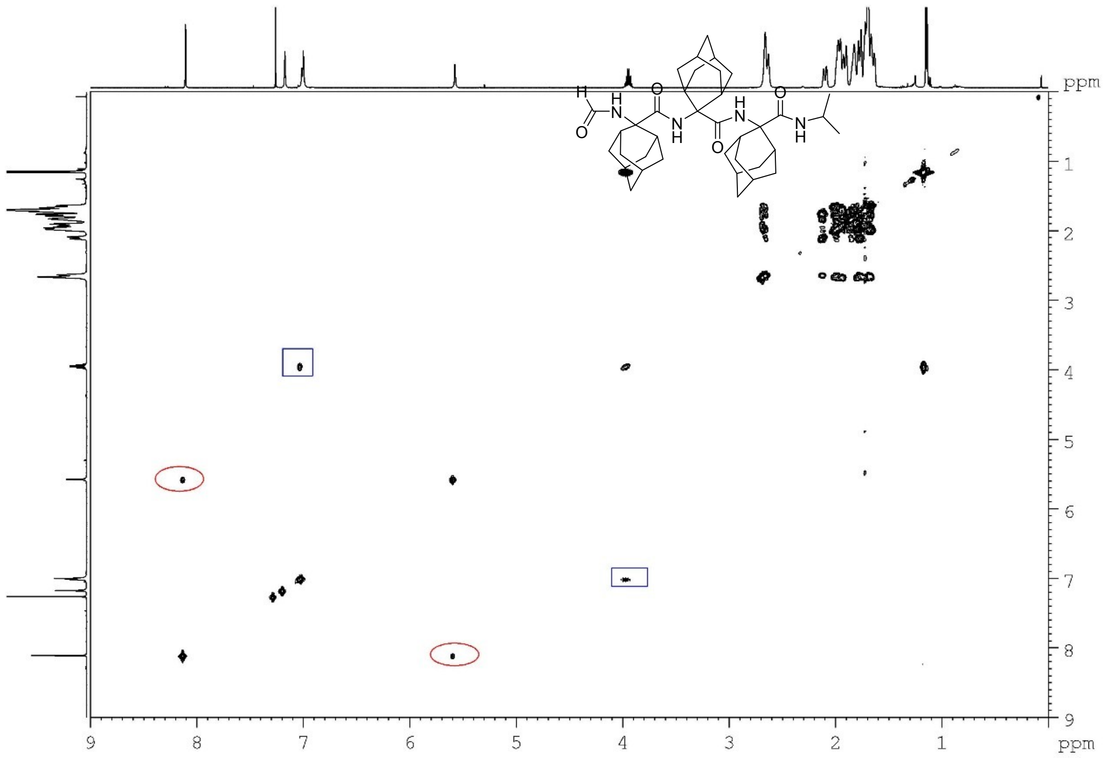
ppm
1
2
3
4
5
6
7
8
9
ppm
9
8
7
6
5
4
3
2
1

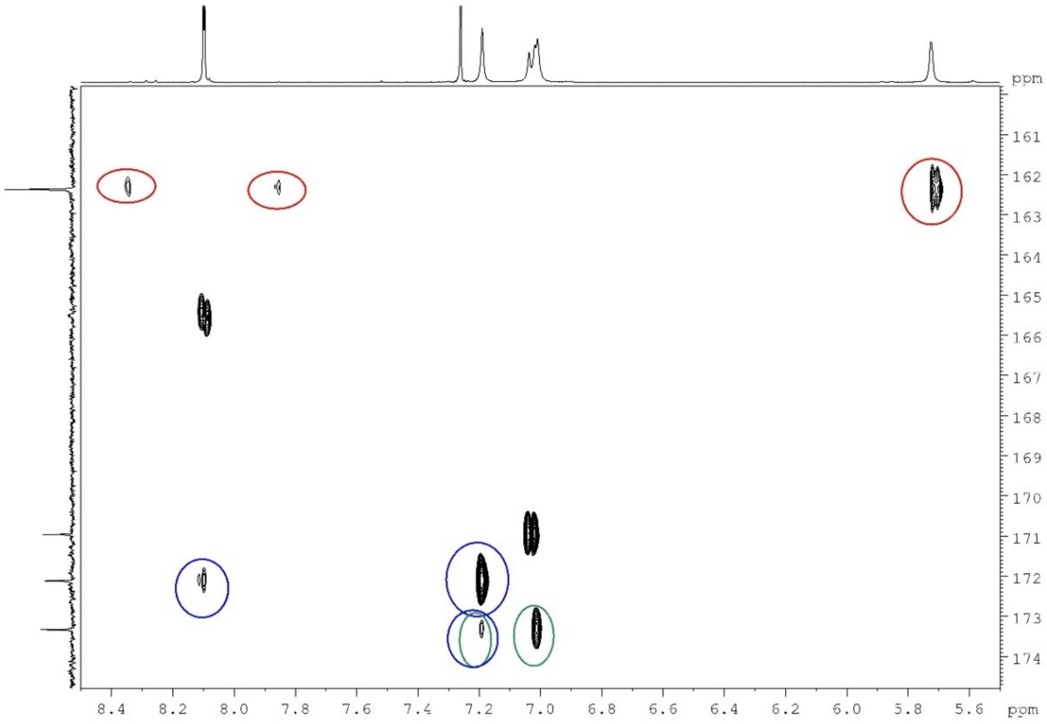
ppm
161
162
163
164
165
166
167
168
169
170
171
172
173
174
8.4
8.2
8.0
7.8
7.6
7.4
7.2
7.0
6.8
6.6
6.4
6.2
6.0
5.8
5.6
ppm

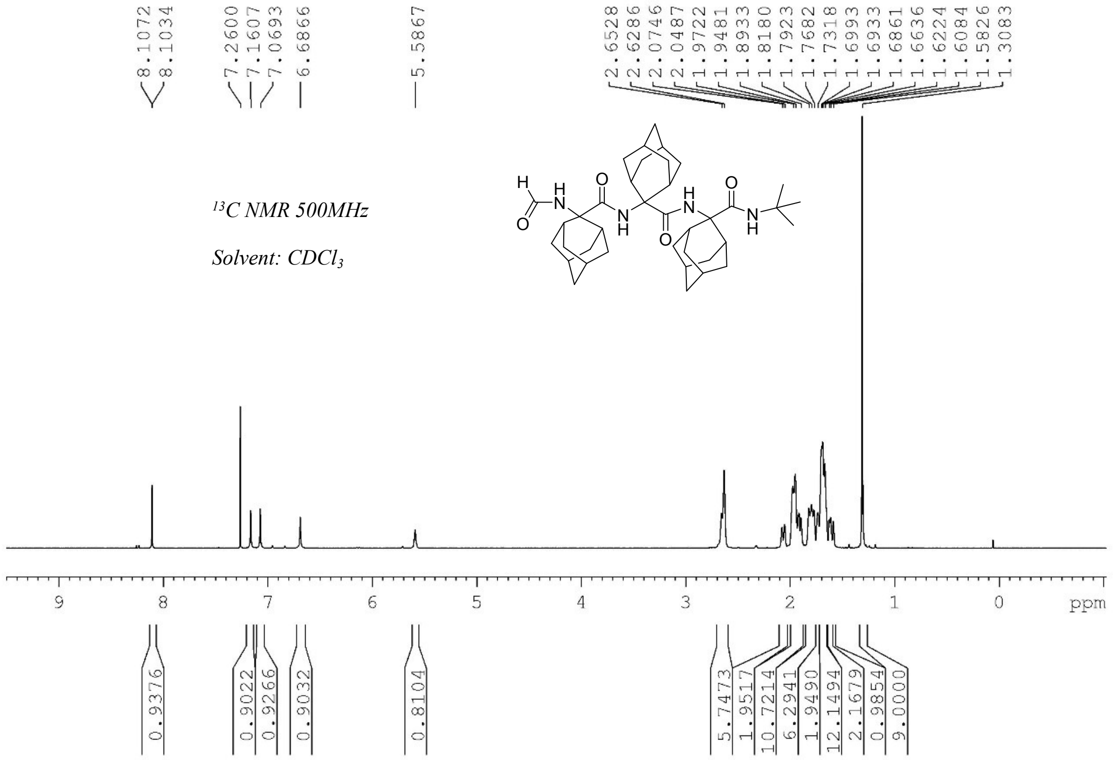
8.1072
8.1034
7.2600
7.1607
7.0693
6.6866
5.5867
2.6528
2.6286
2.0746
2.0487
1.9722
1.9481
1.8933
1.8180
1.7923
1.7682
1.7318
1.6993
1.6933
1.6861
1.6636
1.6224
1.6084
1.5826
1.3083
13C NMR 500MHz
Solvent: CDCl3
H
H
N
O
O
N
H
O
N
H
O
N
H
9
8
7
6
5
4
3
2
1
0
ppm
0.9376
0.9022
0.9266
0.9032
0.8104
5.7473
1.9517
10.7214
6.2941
1.9490
12.1494
2.1679
0.9854
9.0000

Quant.^{13}C NMR 700MHz

Solvent: $CDCl_3$

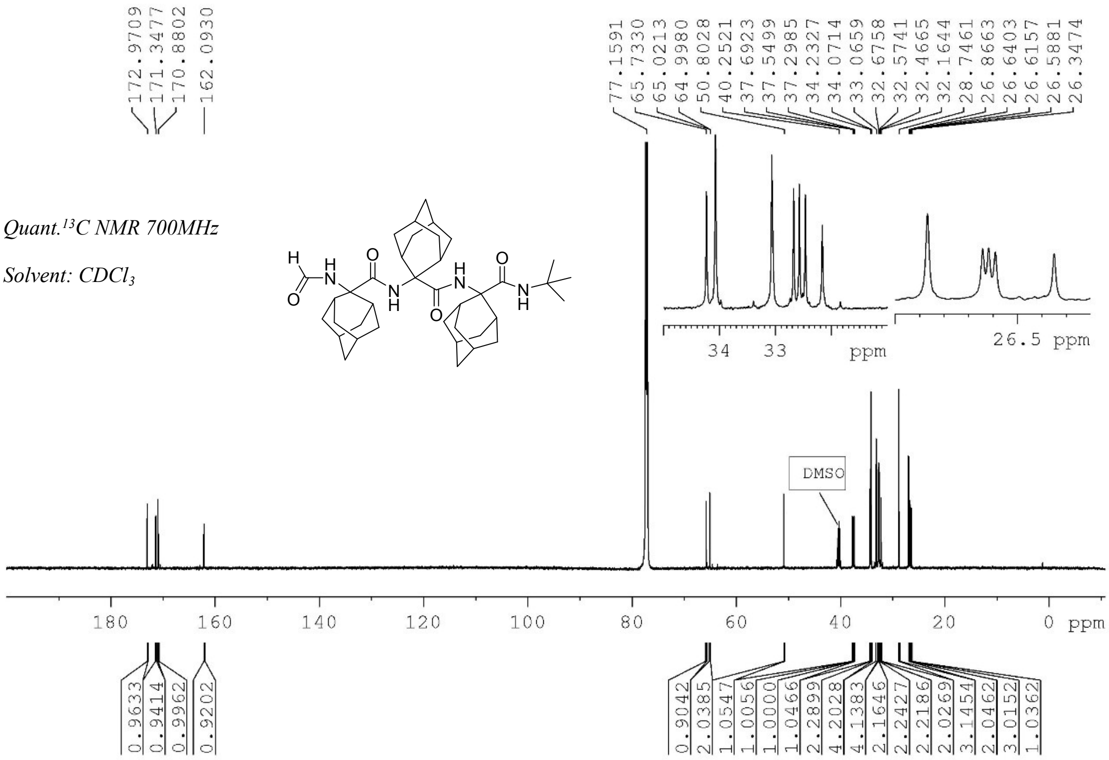

COSY 500MHz (the aliphatic part removed for more clearilty)

Solvent: $CDCl_3$

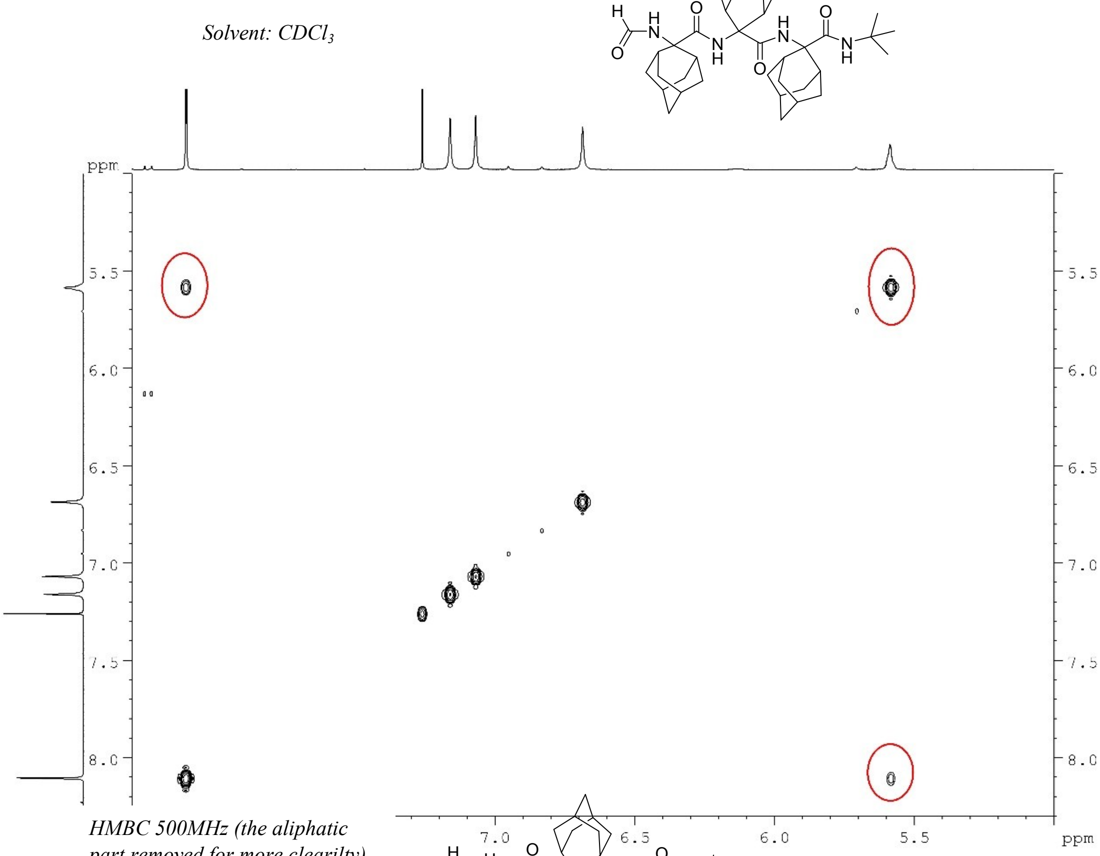

HMBC 500MHz (the aliphatic part removed for more clearilty)

Solvent: $CDCl_3$

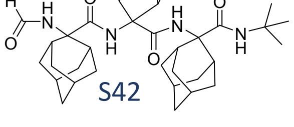

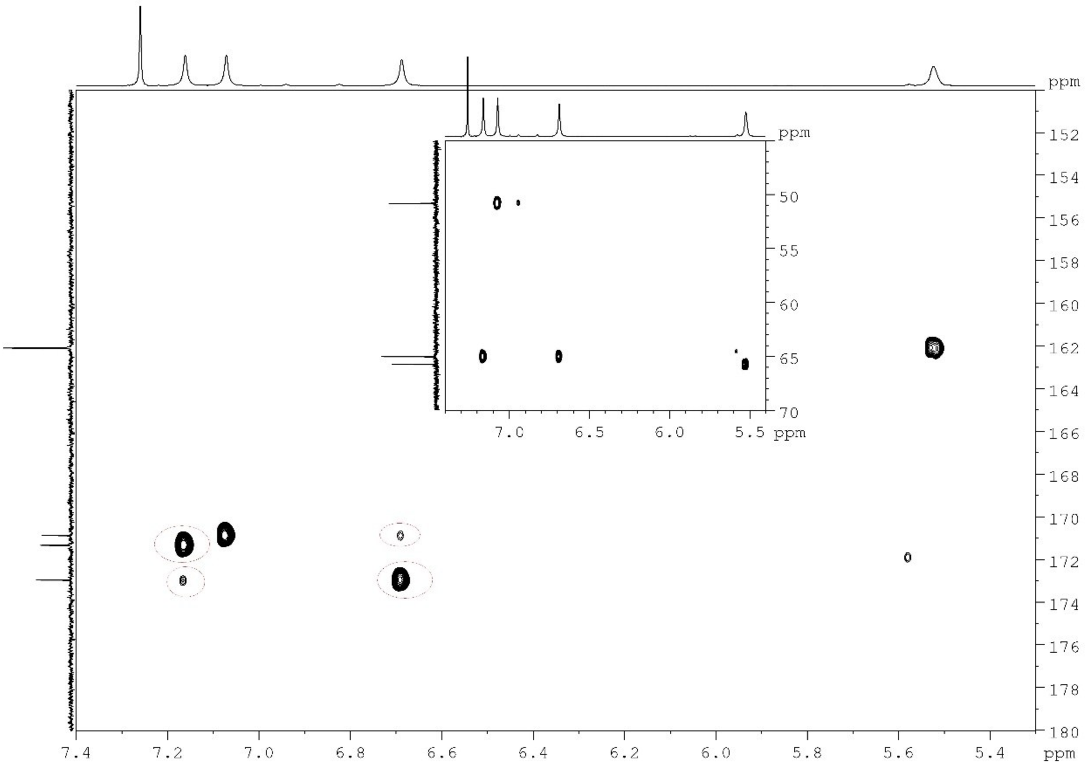
ppm
152
154
156
158
160
162
164
166
168
170
172
174
176
178
180
7.4
7.2
7.0
6.8
6.6
6.4
6.2
6.0
5.8
5.6
5.4
50
55
60
65
70
7.0
6.5
6.0
5.5

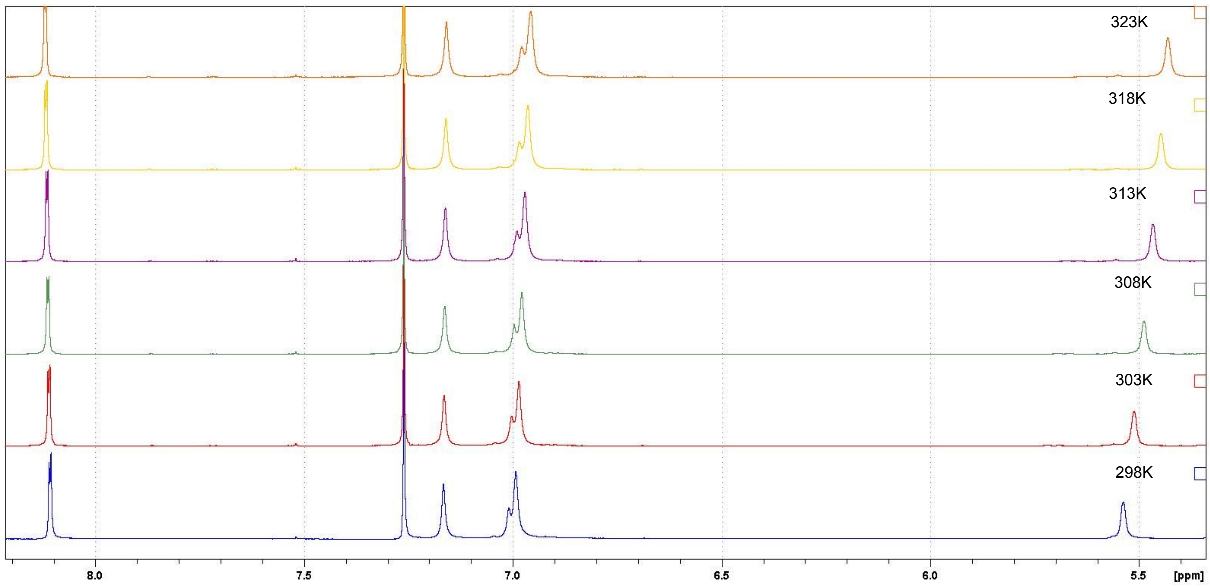

Effect of temperature changes on N-H signal chemical shifts (400 MHz) of **1** in $CDCl_3$.

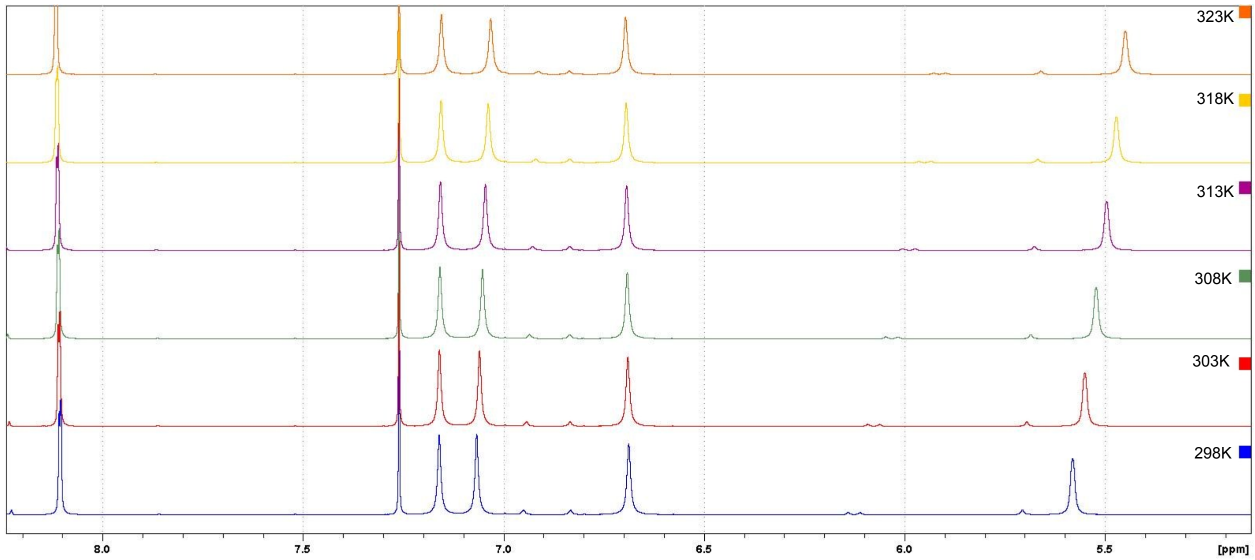

Effect of temperature changes on N-H signal chemical shifts (400 MHz) of **2** in $CDCl_3$.

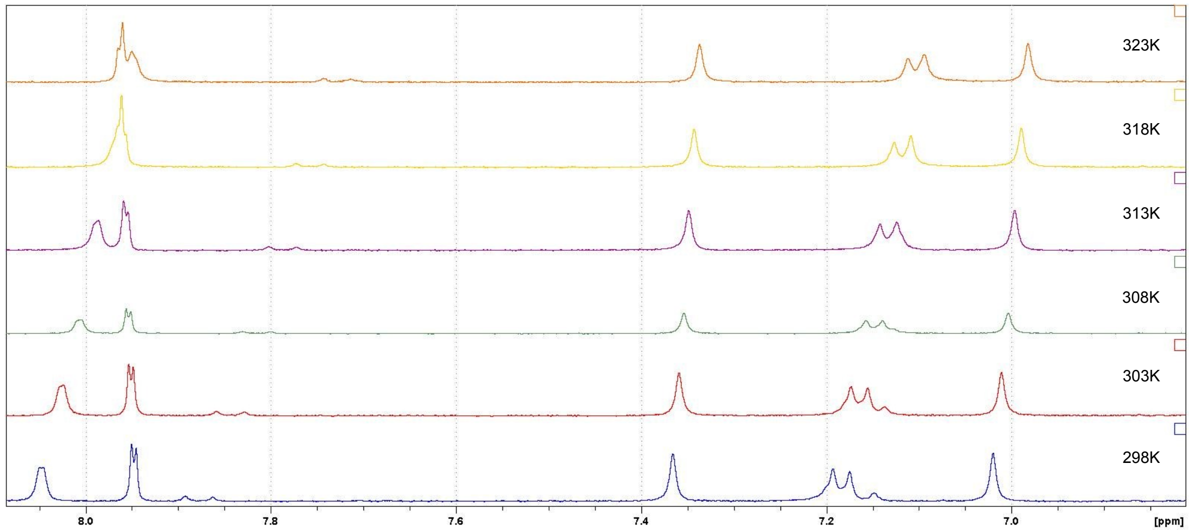

Effect of temperature changes on N-H signal chemical shifts (400 MHz) of **1** in DMSO-*d6*.

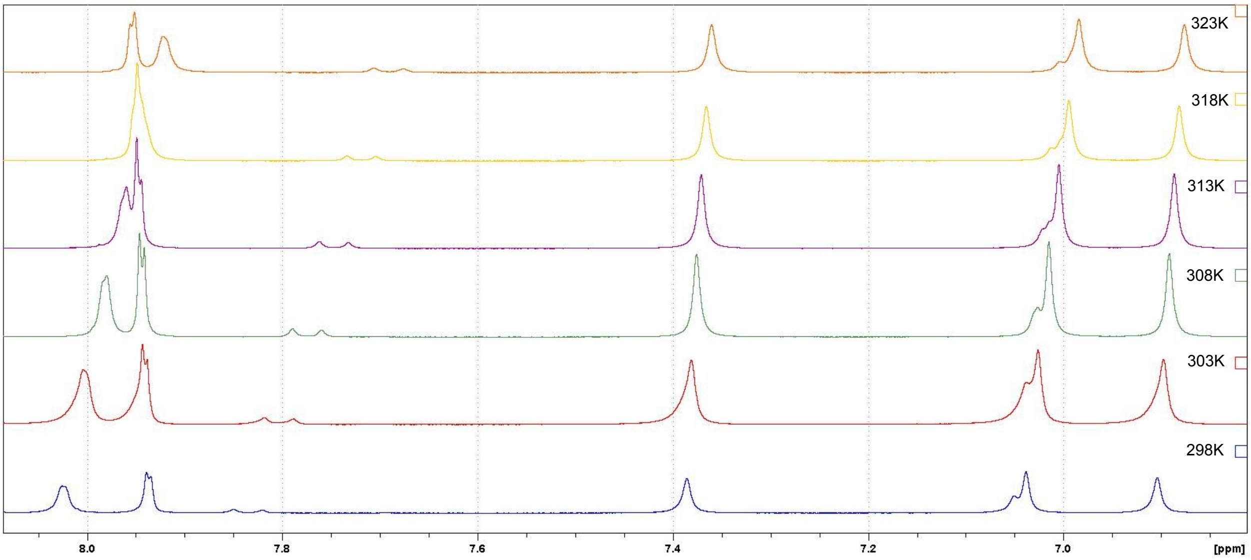

Effect of temperature changes on N-H signal chemical shifts (400 MHz) of **2** in DMSO-*d6*.

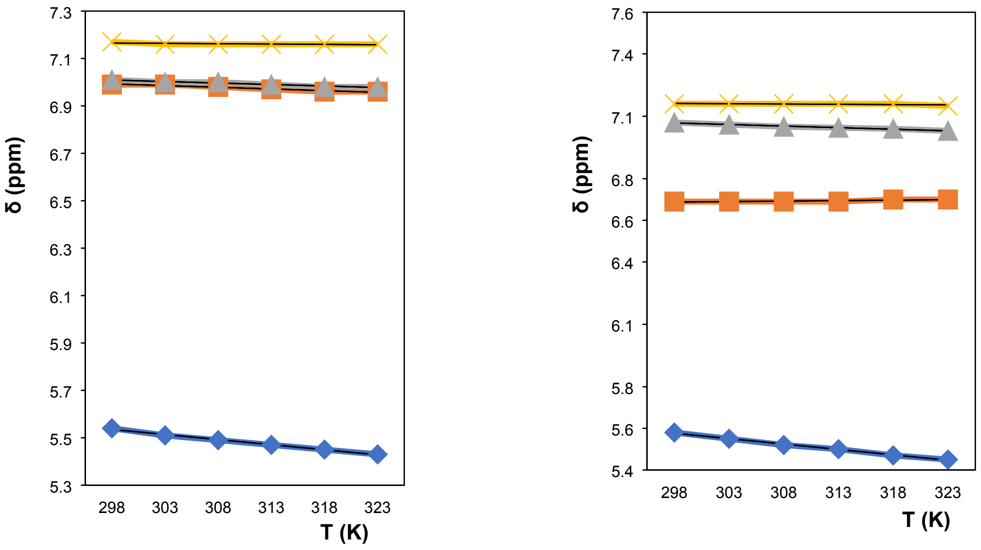

Figure S(1). Plot of changes of NH signal chemical shifts in the NMR spectra of peptides **1** (left) and **2** (right) as a function of temperature in $CDCl_3$.

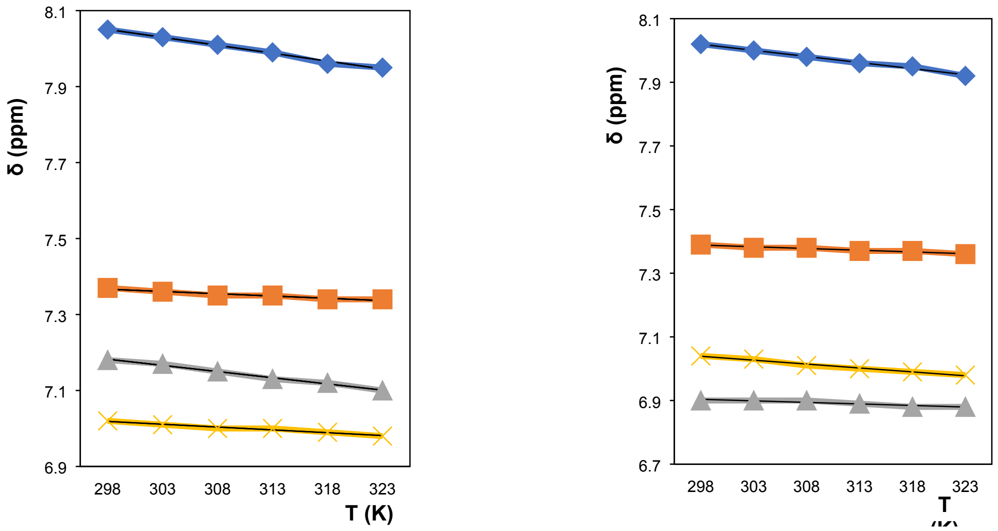

Figure S(2). Plot of changes of NH signal chemical shifts in the NMR spectra of peptides **1** (left) and **2** (left) as a function of temperature in DMSO-*d6*

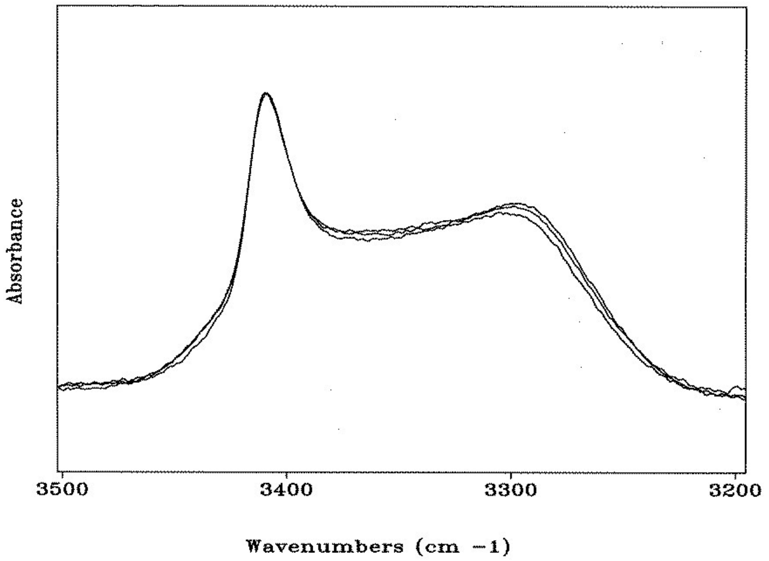

Figure S(3). Overlay of the FT-IR absorption spectra (N-H stretching region) of peptide **1** in $CDCl_3$ solution at the concentrations 10.0 mM, 1.0 mM, and 0.1 mM.

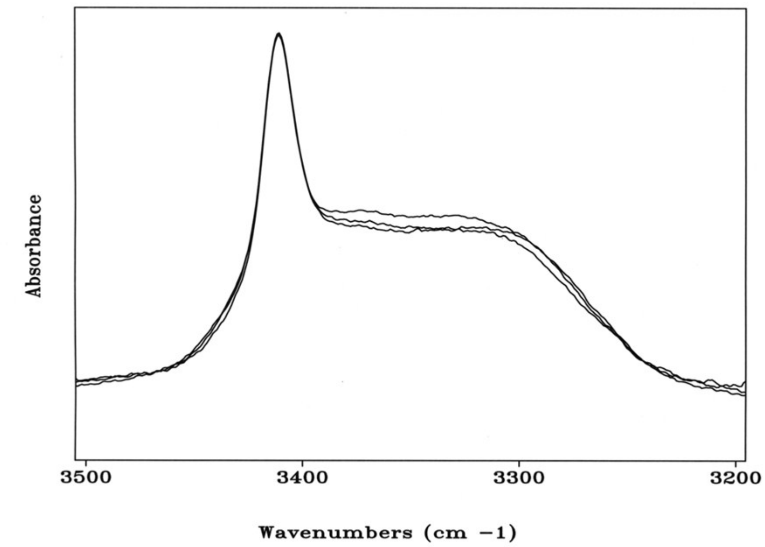

Figure S(4). Overlay of the FT-IR absorption spectra (N-H stretching region) of peptide **2** in $CDCl_3$ solution at the concentrations 10.0 mM, 1.0 mM, and 0.1 mM.

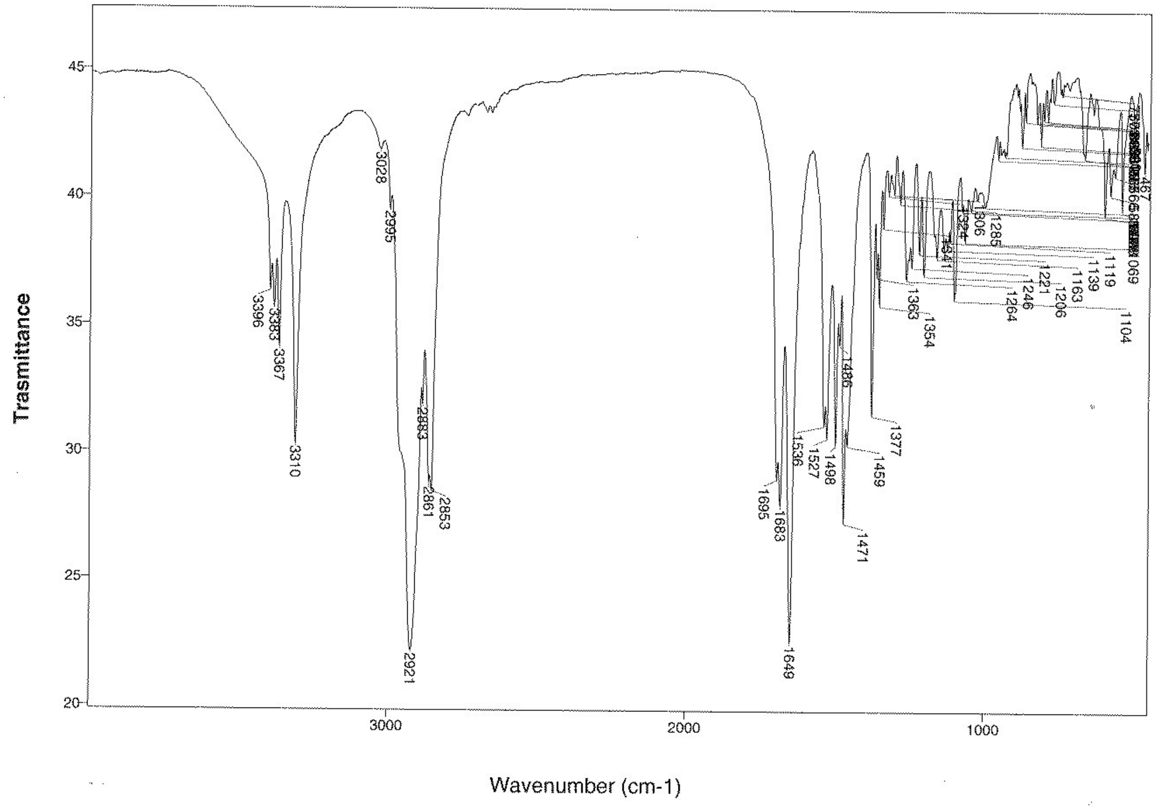

Figure S(5). Solid-state FT-IR absorption spectrum (transmittance mode; KBr disk technique) of crystals of peptide **1** grown from acetone.

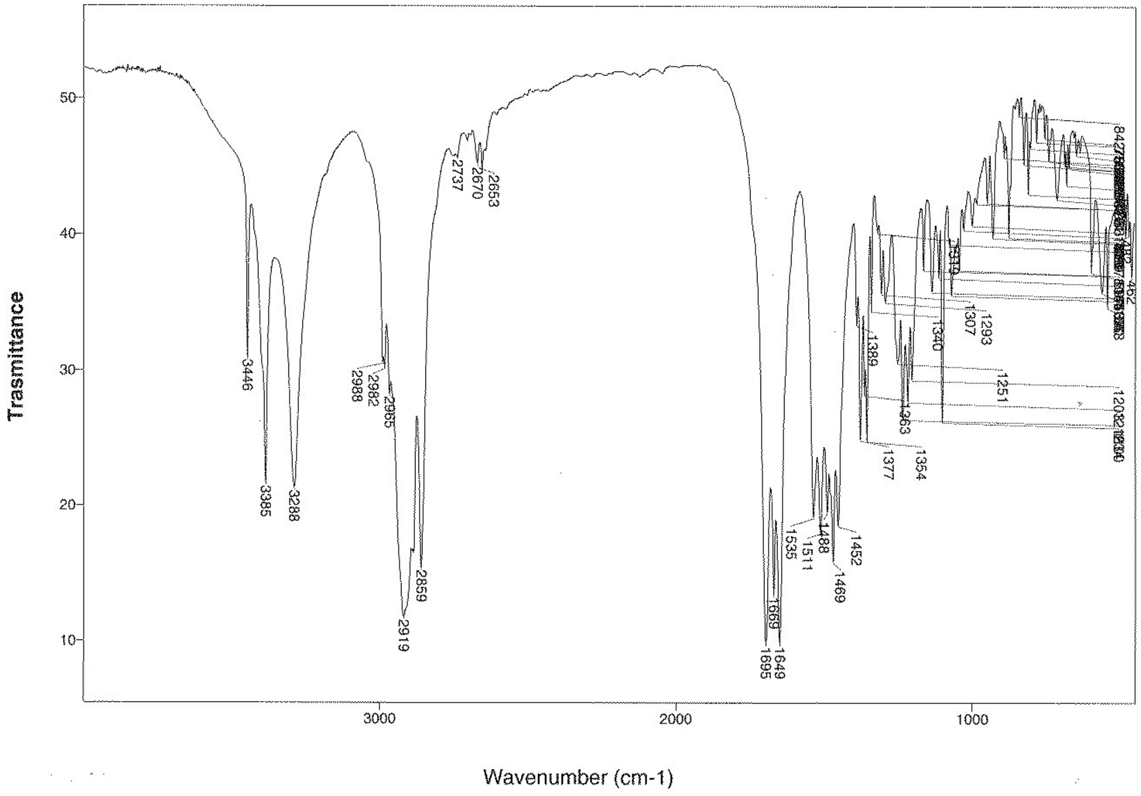

Figure S(6). Solid-state FT-IR absorption spectrum (transmittance mode; KBr disk technique) of crystals of peptide **2** grown from acetone / EtOAc.

SUPPORTING REFERENCES

S1. Dolomanov, O.V., Bourhis, L.J., Gildea, R.J, Howard, J.A.K. & Puschmann, H. (2009), J. Appl. Crystallogr. 42, 339-341.

S2. Sheldrick, G.M. (2015). Acta Crystallogr. A71, 3-8.

S3. Sheldrick, G.M. (2015). Acta Crystallogr. C71, 3-8.
